# Isotope Techniques in Chemical Wastewater Treatment: Opportunities and Uncertainties

**DOI:** 10.1002/anie.202422892

**Published:** 2025-03-18

**Authors:** Hongyu Zhou, Xiaoguang Duan, Bingkun Huang, Shuang Zhong, Cheng Cheng, Virender K. Sharma, Shaobin Wang, Bo Lai

**Affiliations:** ^1^ State Key Laboratory of Hydraulics and Mountain River Engineering College of Architecture and Environment, Sichuan University Chengdu 610065 China; ^2^ School of Chemical Engineering The University of Adelaide Adelaide SA 5005 Australia; ^3^ Department of Chemical, Environmental and Materials University of Miami 1251 Memorial Drive Coral Gables Florida 33146 USA

**Keywords:** Chemical wastewater treatment, Element tracing, Isotope technologies, Kinetic isotope effects, Mechanism investigation

## Abstract

A comprehensive and in‐depth analysis of reaction mechanisms is essential for advancing chemical water treatment technologies. However, due to the limitations of conventional experimental and analytical methods, the types of reactive species and their generation pathways are commonly debatable in many aqueous systems. As highly sensitive diagnostic tools, isotope techniques offer deeper insights with minimal interference from reaction conditions. Nevertheless, precise interpretations of isotope results remain a significant challenge. Herein, we first scrutinized the fundamentals of isotope chemistry and highlighted key changes induced by the isotope substitution. Next, we discussed the application of isotope techniques in kinetic isotope effects, presenting a roadmap for interpreting KIE in sophisticated systems. Furthermore, we summarized the applications of isotope techniques in elemental tracing to pinpoint reaction sites and identify dominant reactive species. Lastly, we propose future research directions, highlighting critical considerations for the rational design and interpretation of isotope experiments in environmental chemistry and related fields.

## Graphical abstract

In this review, we would first introduce basic knowledge about isotopic compounds and then scrutinize the physical and chemical influence induced by isotope changes. Special attention will be paid to the uncertainties in explaining the isotope results. Next, the implications in kinetic isotope effects (KIE, including solvent and nonsolvent KIE) and element tracing will be individually discussed. Notably, the combination of isotope techniques with EPR, infrared, Raman, and DFT calculation will be summarized. Finally, the limitations and outlooks will be proposed.

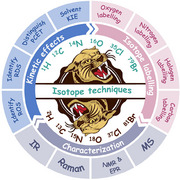

## 1 Introduction

Water contamination caused by rapid urbanization and industrial development has posed a significant threat to water safety and human health. To address this issue, various chemical treatment processes have been explored and developed in recent years, including advanced oxidation processes (AOPs, like Fenton oxidation,^[^ ^1^, ^2^ ^]^ activated persulfate oxidation,^[^ ^3^, ^4^ ^]^ catalytic ozonation,^[^ ^5^ ^]^ electrochemical oxidation,^[^ ^6^ ^]^ and photochemical oxidation^[^ ^7^ ^]^) and advanced reduction processes (ARPs, like ultraviolet/sulfite reduction (UV/sulfite)^[^ ^8^ ^]^). All these technologies leverage reactive oxygen species (ROS) (e.g., hydroxyl radicals (^•^OH), sulfate radicals (SO_4_^•−^), singlet oxygen (^1^O_2_), and high‐valent metal‐oxo species (HMOS)) or reactive reduction species (RRS) (e.g., hydrated electrons (e_aq_^−^)), to complete the targeted redox reactions. Therefore, gaining a deeper understanding of the types, formation mechanisms, and reaction pathways of reactive species (RS) is crucial for developing and optimizing these treatment processes.

Conventional techniques for mechanistic studies include quenching experiments, probe experiments, and spectroscopy characterization. However, these techniques are limited by their intrinsic drawbacks, leading to uncertainties in mechanism explanation. For example, many works have pointed out that the presence of some chemical quenchers can substantially consume the oxidant, interfere with ROS production, or block the catalyst's active sites, thereby exaggerating the contribution of RS in contaminant degradation.^[^ ^9^, ^10^, ^11^, ^12^, ^13^ ^]^ The application of other important techniques, i.e., electron paramagnetic resonance (EPR), is also impeded by the inaccurate spectrum analysis and overlapping signals of spin‐trapped adducts generated through different generation pathways.^[^ ^14^, ^15^ ^]^ For example, the appearance of 5,5‐dimethyl‐1‐pyrroline N‐oxide‐OH (DMPO‐OH) signal is usually ascribed to the presence of ^•^OH. However, the adduct of SO_4_^•−^ with DMPO, i.e., DMPO‐SO_4_, could also hydrolyze into DMPO‐OH along with the time.^[^ ^16^ ^]^ Additionally, HMOS could oxidize DMPO via one‐electron transfer, and the oxidized product then hydrolyze to DMPO‐OH, a process known as the Forrester−Hepburn mechanism.^[^ ^17^ ^]^ Similar challenges are encountered in identifying ^1^O_2_, as a direct electron‐transfer process can also produce false positive signals of 2,2,6,6‐tetramethylpiperidine N‐oxyl (TEMPO), thus complicating the interpretation.^[^ ^3^ ^]^

Isotope techniques offer a solution to this Gordian knot by leveraging their fingerprint characteristics. Isotopes of the same element share an identical number of protons but differ in the number of neutrons, which endows them with similar chemical reactivity.^[^ ^18^ ^]^ However, differences in reaction kinetics, vibrational frequency, and other properties allow for detailed investigation of the reaction mechanism at both atomic and chemical bond levels. Compared with conventional techniques, isotope techniques significantly minimize the interference and preserve the reaction conditions, offering a more precise approach to unraveling complex mechanisms under real reaction scenarios.

Generally, the isotope techniques can be classified into two categories, including kinetic isotope effects (KIE) and isotope‐labeling (or elemental tracing). The former refers to the comparison of the reaction rates after the isotope substitution of specific atoms. According to the element species, KIE can be classified into ^1^H/^2^H KIE and heavy atom KIE, like ^12^C/^13^C KIE, ^14^N/^15^N KIE, ^16^O/^18^O KIE, and ^35^Cl/^37^Cl KIE.^[^ ^19^, ^20^ ^]^ The calculations of ^1^H/^2^H KIE can be achieved through direct measurement and comparison of reaction rates if heavy and light isotopologues are independently brought to the reaction (noncompetitive approach). Alternatively, kinetic isotope effects can be derived by analyzing the ratio of heavy over light isotopologues when both undergo reaction in the same experiment (competitive approach). Heavy atom KIE can only be determined through this latter approach because the subtle difference in reaction rates would otherwise be dwarfed by fluctuations in experimental conditions (temperature, etc.). Such precise isotope ratio measurements are typically conducted on isotope ratio mass spectrometers. In contrast, isotope‐labeling experiments, combined with quadrupole or time‐of‐flight mass spectrometer analysis, are usually used to identify the reaction species, trace the origins of functional groups, analyze the degradation products, and exclude the interference of impurities. By leveraging this effective tool, new mechanistic insights were obtained in some well‐established systems, such as Fe(II)/peroxydisulfate process, which had been long reckoned to generate ^•^OH and SO_4_^•−^ as the sole ROS while the role of ferryl iron Fe(IV) was previously ignored.^[^ ^16^, ^21^ ^]^ Despite the advantages of applying isotope techniques in mechanistic studies, there still exist some uncertainties and wrong interpretations of the experiment results in the references. For example, the possible isotope exchange was not considered when discussing the oxygen isotope distribution of CO_2_ during the mineralization of perfluorooctanoic acid (PFOA).^[^ ^22^ ^]^ Furthermore, only a limited number of reviews introduced the application of isotope techniques in wastewater treatment, and the relative discussions on this topic remain insufficiently comprehensive.^[^ ^23^, ^24^, ^25^, ^26^, ^27^, ^28^, ^29^ ^]^

In this review, we systematically summarized the fundamentals and applications of isotope techniques in chemical wastewater treatment. We begin by introducing the fundamental concepts of isotopic compounds, and then examine the physical and chemical effects induced by isotope changes. Subsequently, we discussed the implications of KIE and element tracing individually. During the discussion of KIE, we first focused on ^1^H/^2^H KIE using isotope‐enriched reagents, and then discussed heavy atom KIE and compound‐specific isotope analysis at natural isotope abundance. Notably, the combination of isotope techniques with other characterization methods, such as EPR, infrared and Raman spectrometer, and density functional theory (DFT) calculation, would also be highlighted. Finally, we address the limitations of isotope techniques and propose future research directions.

## 2 Fundamentals of Isotopes

### 2.1 Commercially Available Isotopic Compounds

**Figure 1:**
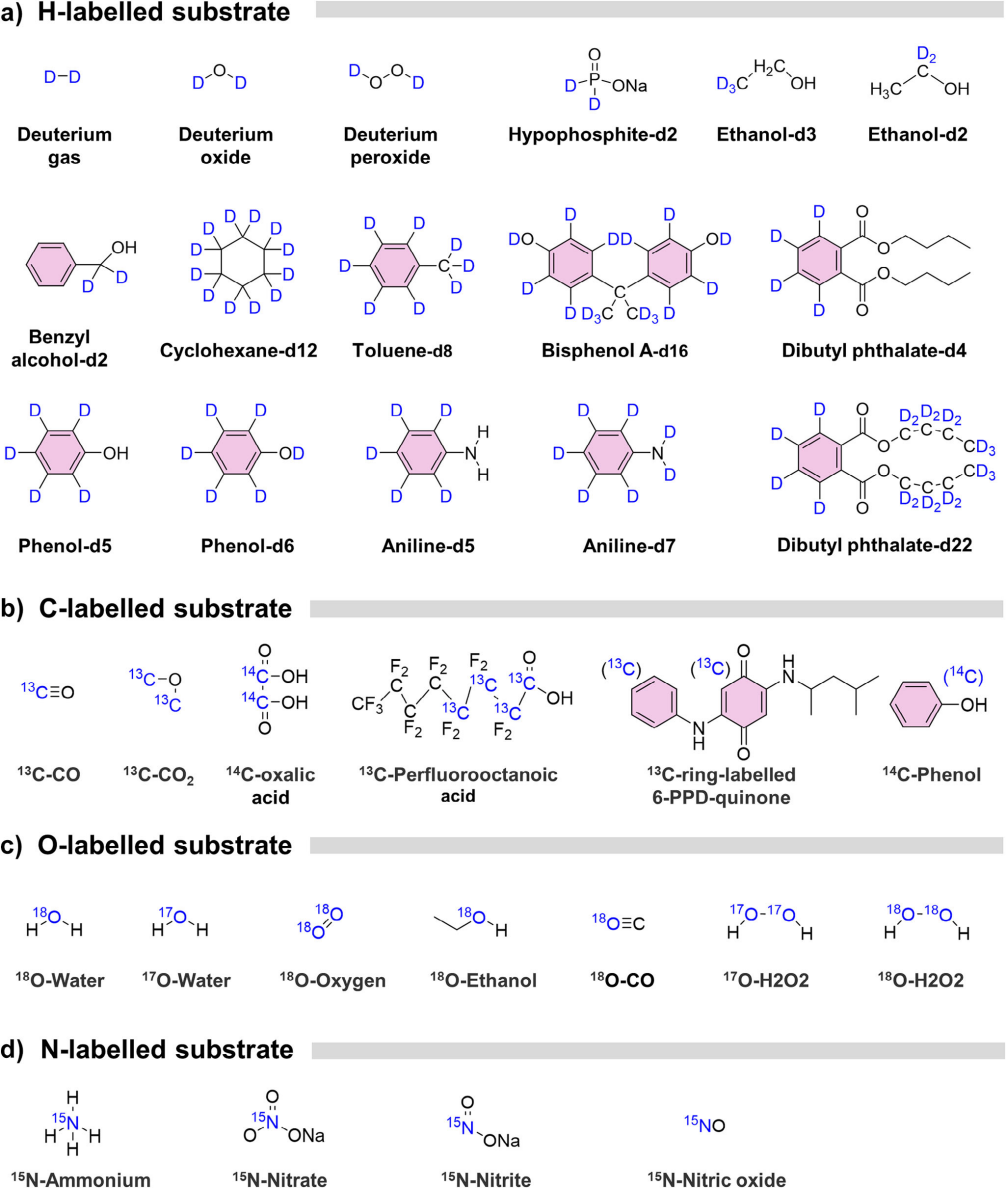
Commonly used isotope‐enriched and commercially available compounds for the investigation in chemical wastewater treatment.

Most isotopes are short‐lived radioactive species; however, the isotopes relevant to our focus constitute only a small subset. The most frequently used isotopes include ^1^H/^2^H, ^12^C/^13^C/^14^C, ^14^N/^1^N, ^16^O/^17^O/^18^O, ^35^Cl/^37^Cl, and ^79^Br/^81^Br, as these isotopes cover most of the reactions or reactive species of interest (Table S1). Typical commercially available compounds of these isotopes are summarized in Figure 1. Many of them either serve as model compounds for mechanistic studies (e.g., D_2_O, ethanol‐d2, phenol‐d5, aniline‐d5, H_2_^18^O, and ^15^N‐nitrate) or are frequently detected in wastewater and thus warrant particular attention (e.g., bisphenol A‐d16, dibutyl phthalate‐d22, and ^13^C‐ring‐labeled 6‐PPD‐quinone).^[^ ^30^, ^31^, ^32^, ^33^, ^34^, ^35^, ^36^ ^]^ Depending on the application scenario, these commercial compounds can be grouped into three categories. (1) Directly used for the kinetic experiment: most hydrogen‐labeled compounds (e.g., ethanol‐d2, phenol‐d5) can be used without specialized instruments. These substrates help elucidate reaction mechanisms involving hydrogen abstraction or transfer. (2) Element tracing to study the reaction pathways: Heavy atom‐labeled compounds (e.g., ^13^C‐, ^15^N‐, or ^18^O‐labeled) and some H‐labeled analogs (e.g., bisphenol A‐d22) are primarily employed for tracing reaction routes. In these cases, liquid/gas chromatographs combined with quadrupole or time‐of‐flight mass spectrometer are usually required for analysis. (3) In situ spectrometer and nuclear magnetic resonance (NMR) characterization: Specific compounds (like D_2_O, H_2_^18^O, ^13^CO_2_, and ^15^NO_3_^−^) are especially useful for in situ spectroscopic or NMR measurements. For instance, although both ^17^O and ^18^O can track oxygen‐atom migration, only ^17^O has a nuclear spin, making it suitable for EPR and NMR investigations of reaction mechanisms.^[^ ^37^, ^38^ ^]^

In contrast, mechanistic studies on heavy element isotope effects are frequently conducted with non‐labeled compounds at natural isotopic abundance. This approach will be discussed at a later stage.

Notably, the functional groups of concern can be selectively labeled, as in the case of dibutyl phthalate‐d_4_ and phthalate‐d_22_, allowing selective comparison of the site‐specific reactivity.^[^ ^36^ ^]^ Moreover, compared with ^13^C, ^14^C is a radioactive nucleus of carbon, which can be detected through a liquid scintillation counter.^[^ ^39^, ^40^ ^]^ This characteristic provides alternative measurement for some small molecules, such as formic acid and oxalic acid,^[^ ^39^ ^]^ and facilitates the tracing of total organic carbon (TOC) in the solution or on the catalyst surface.^[^ ^40^ ^]^ For less common isotope‐substituted substances, two main strategies are available for their synthesis: (1) selection of isotope‐labeled precursors, such as ^18^O_2_, H_2_^18^O_2_, and ^15^NH_4_Cl, for the synthesis of ^18^O_3_, CH_3_CO^18^O^18^OH, and ^15^NH_2_Cl,^[^ ^41^, ^42^, ^43^, ^44^, ^45^ ^]^ or (2) incubating reactants in isotope‐enriched solvents like K_2_FeO_4_ and KIO_4_.^[^ ^46^, ^47^ ^]^

### 2.2 Representative Chemical Probes for Isotope Labeling

**Figure 2:**
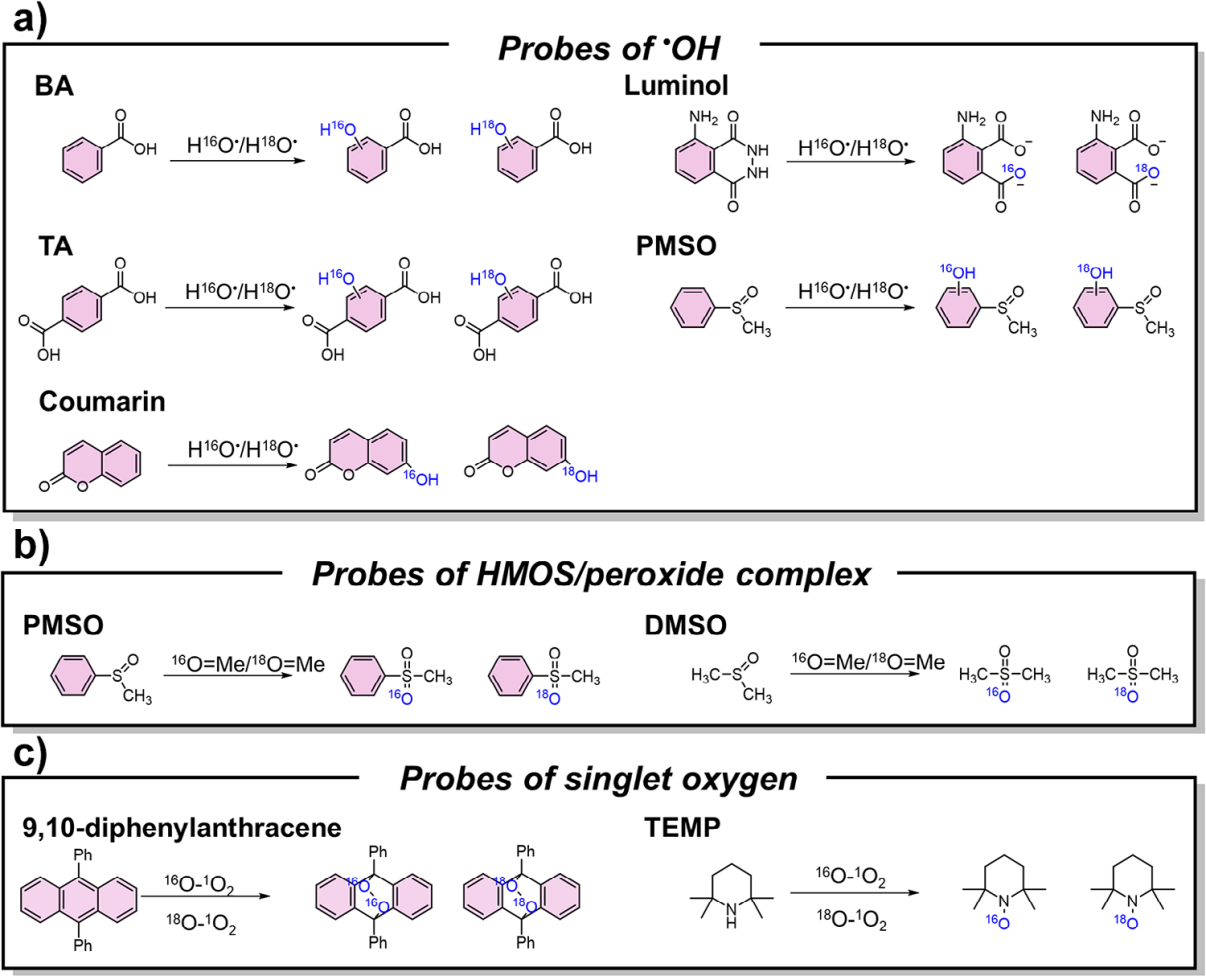
Commonly used probes for capturing the transient species.

In addition to isotope‐enriched reagents, certain chemical probes are frequently used to capture the isotope‐labeled transient species that cannot be directly distinguished through MS or other instruments (Figure 2). Benzoic acid (BA),^[^ ^48^ ^]^ terephthalic acid (TA),^[^ ^49^ ^]^ luminol,^[^ ^50^ ^]^ coumarin,^[^ ^51^ ^]^ and methyl phenyl sulfoxide (PMSO)^[^ ^52^ ^]^ are the benchmark probes to capture ^•^OH, yielding the corresponding hydroxylated products. The characteristic +2 shifts of *m/z* values in MS and secondary MS spectrum (MS/MS) indicate the incorporation of an ^18^O‐labeled hydroxyl group in the products. In contrast, dimethyl sulfoxide (DMSO) and PMSO are effective for the detection of HMOS and peroxide complex due to their characteristic oxygen atom transfer reaction with sulfoxides to produce featured dimethyl sulfone (DMSO_2_) and methyl phenyl sulfone (PMSO_2_). Notably, PMSO can be simultaneously used for the detection of ^•^OH and HMOS based on the different MS/MS spectrums of hydroxylated‐PMSO (PMSO‐OH) and PMSO_2_. A more detailed comparison of the advantages and limitations of different probes is provided in Table S2. Finally, 9,10‐diphenylanthracene and 2,2,6,6‐tetramethylpiperidine (TEMP) could readily react with ^1^O_2_ and incorporate the oxygen atoms from ^1^O_2_. Isotope substitution of ^1^O_2_ with ^18^O results in characteristic +4 and +2 *m/z* shifts in their products, respectively.^[^ ^53^, ^54^ ^]^

### 2.3 Potential Changes Induced by Isotope Substitution

#### 2.3.1 Solution pH/pD and Coefficient of Acidity (pKa)

Deuterium oxide (D_2_O) is one of the most important deuterium solvents in isotopic studies, as it closely mimics the reaction conditions of H_2_O. However, when measuring the acidity of D_2_O solution, i.e., measuring the concentration of D^+^ in the solution, the value of the pH meter does not accurately represent the actual D^+^ concentration. Through comparisons across different instruments, an empirical value of 0.4 was obtained as the difference between the pH meter reading and the actual pD value (Equation 1)^[^ ^55^ ^]^

(1) $$\mathrm{pD}=pH_{\mathrm{reading}}+0.4.$$

Besides, the ionization constants of D_2_O and H_2_O at 25 °C are 1.6 × 10^−15^ and 1 × 10^−14^, respectively, resulting in a difference of 0.8 in their p*K*_W_ values (p*K*_W_(D_2_O) – p*K*_W_(H_2_O) = 0.8). Consequently, even when the concentrations of H^+^ and D^+^ are equivalent, the concentration of OD^−^ would be lower than that of OH^−^ at 25 °C. This distinction should be considered especially for base‐catalyzed reactions. For example, the decomposition of O_3_ is accelerated under high alkalinity, requiring the initial pH to be set to *x* and initial pD to *x* + 0.8 to accurately study the effects of OD^−^ on O_3_ decomposition.^[^ ^56^ ^]^

**Figure 3:**
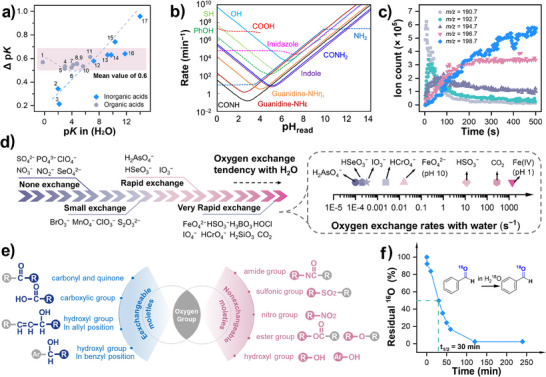
a) Relationship between the pKa of weak acids in H_2_O and the pKa difference in H_2_O and D_2_O. 1. o‐nitroanilinium ion, 2. H_2_SO_4_ (second step), 3. H_3_PO_4_ (first step), 4. m‐nitroanilinium ion, 5. 4‐chloro‐2,6‐dinitrophenol, 6. Citric acid [(pKa_1_ + pKa_2_)/2], 7. Succinic acid (first step), 8. Acetic acid, 9. Deuterioacetic acid, 10. Succinic acid (second), 11. 3,5‐dinitrohphenol, 12. H_3_PO_4_ (second step), 13. H_3_BO_3_, 14. PMS, 15. HCO_3_−, 16. H_2_O_2_ and 17. H_2_O. Data is from references.^[^ ^10^, ^57^, ^58^ ^]^ b) The hydrogen/deuterium exchange rate of various functional groups as a function of the solution pH. Copyright 2020 American Chemical Society.^[^ ^59^ ^]^ c) m/z ion traces corresponding to the evolution of IO_4_− species over time. Copyright 2011 Springer Nature.^[^ ^60^ ^]^ d) Oxygen exchange tendency of various inorganic oxyanions with H_2_O. e) Comparison of oxygen exchange tendency of various organic functional groups with H_2_O. f) Oxygen exchange between benzaldehyde and H_2_^18^O as a function of time. Copyright 2015 American Chemical Society.^[^ ^61^ ^]^

In addition to solvent, the dissociation behavior of weak acids or bases is also influenced by D_2_O. As shown in Figure 3a, the difference of the p*K* values in H_2_O and D_2_O (p*K*_a_^D^‐p*K*_a_^H^) follows a valley‐shaped curve as a function of p*K*_a_^H^, with a mean difference of 0.6.^[^ ^57^ ^]^ This variation alters the species distribution of substrate even under identical pH or pD conditions. For example, Yang et al. investigated the effects of D_2_O on the decomposition of peroxymonosulfate (PMS).^[^ ^10^ ^]^ Although the condition was controlled at [D^+^] = [H^+^] = 1 × 10^−9^ M, the p*K*_a2_^D^ of PMS (9.76) is 0.63 higher than the p*K*_a2_^H^ (9.13). This disparity led to notable differences in proportions of HSO_5_^−^ and SO_5_^2−^ under this condition. Therefore, the author allowed for the difference in species concentrations when calculating the KIE.

#### 2.3.2 Isotope Exchange with Solvent

Hydrogen/deuterium exchange: Beyond its effects on species distribution, D_2_O can substitute the active protons of many functional groups via hydrogen/deuterium exchange (HDX) reactions. Hamuro et al.^[^ ^59^ ^]^ summarized and predicted the HDX rates of ─SH, ─OH, Ph─OH, ─NH_2_, indole, and ─CONH─ via the proton transfer theory (Figure 3b). Most of these exchange reactions are catalyzed either by bases, where the proton acceptor is OD⁻, or by acids, where the deuteron donor is D_3_O⁺. As a result, the pseudo‐first‐order rates of HDX reactions show a strong pH dependence with a minimal point of about 1 min^−1^, suggesting that the functional group of various contaminants would be rapidly deuterated upon dissolution in D_2_O. Conversely, deuterio substances would be quickly hydrogenated upon dissolution in H_2_O. Therefore, D_2_O must be selected as the solvent for investigating reactions involving such functional groups.^[^ ^62^ ^]^ For example, the reaction between phenolic−OH and Fe(IV)/Cr(V) must be conducted in D_2_O to investigate the KIE effects of phenolic−OH.^[^ ^63^ ^]^ In contrast, when studying reactions between alkyl C─H bonds and some ROS, intrinsically labeled compounds should be used, as no significant isotope exchange would occur. However, for the C─H bond connected with strong electron‐withdrawing groups like nitromethane^[^ ^64^ ^]^ and 3‐methoxyacetophenone,^[^ ^65^ ^]^ the exchange of C─H bond cannot be disregarded. Finally, for the X─H (X = C, N, O, and S) bond in solid material, the extent and rate of solvent exchange remain poorly understood. Real‐time monitoring using solid‐state NMR could provide valuable insights into the rates and extent of H/D exchange in these systems.

Oxygen exchange reaction typically occurs between solvent H_2_^18^O and inorganic oxyanions or organic functional groups. The reversible hydration/dehydration reaction would gradually reach equilibrium in the solution, resulting in the labeling of substances with ^18^O.^[^ ^66^ ^]^ As depicted in Figure 3c, periodate (PI) I^16^O_4_^−^ would quickly transform into I^18^O_4_^−^ within 5 min in H_2_^18^O solution.^[^ ^60^ ^]^ Similarly, species like HSO_3_^2−^, CO_2_, H_3_BO_3_, HCrO_4_^−^, HOCl, and FeO_4_^2−^ all proceed rapid oxygen exchange in water with a half‐life period ranging from 0.0025 s to 4.81 min (Figure 3d and Table S3).^[^ ^67^ ^]^ In contrast, MnO_4_^2−^ and S_2_O_3_^2−^ require stricter reaction conditions, such as very low pH or high temperature. Due to their stability, Wiberg et al.^[^ ^68^ ^]^ prepared the ^18^O‐labeled KMnO_4_ through an extended exchange and recrystallization process, achieving ^18^O incorporation efficiency of 1.114%.

For organic functional groups, only carbonyl, quinone, carboxylic groups (both two oxygen atoms), and hydroxyl groups in allyl position and hydroxyl groups in benzyl position are exchangeable. In contrast, nitro group, sulfonic group, amide group, ester group, and other hydroxyl groups (including phenolic‐OH) are nonexchangeable (Figure 3e).^[^ ^69^ ^]^ Specifically, the oxygen exchange in carbonyl groups is driven by the reversible hydration of the electrophilic carbon in the C═O bond, which shows an acid‐catalyzed characteristic. The aliphatic aldehydes exhibit faster hydration rates than aromatic aldehydes due to the smaller resonance stabilization effects. Shi et al.^[^ ^61^ ^]^ observed that 100% oxygen exchange of benzaldehyde is achieved within 3 h, and thus, the exchange time of aliphatic aldehydes must be shorter than 3h (Figure 3f). In contrast, the oxygen exchange between carboxylic groups and H_2_^18^O occurs at a much slower rate. For example, in the case of acetic acids, only 23% of molecules exchange one of the oxygen atoms with H_2_^18^O at pH 2 and 25 °C after 18 h, whereas only 2.5% of molecules exchange both oxygen atoms under identical conditions.^[^ ^70^ ^]^ The exchange occurs via hydronium ion attack on the neutral acid molecule, and a higher temperature and acidity are conducive to the reaction (Table S4).^[^ ^69^, ^71^ ^]^

Given the common H/D and oxygen exchange phenomenon, it is crucial to consider the possibility of element exchange when investigating the mechanism of a reaction. However, these exchange phenomena can also be applied to identify unknown compounds through MS analysis.^[^ ^69^, ^72^ ^]^ Kostyukevich et al.^[^ ^72^ ^]^ reported that the *m/z* shifts of parent ions and daughter ions after exchange with D_2_O or H_2_^18^O could help screen the structures of known compounds and herein reduce the analysis workload up to 13 times.

#### 2.3.3 Solubility

Also, solubility is influenced by the solvent exchange to D_2_O. A smaller saturated concentration is observed in D_2_O compared to H_2_O.^[^ ^73^ ^]^ However, this effect is generally minor, as most reaction conditions occur well below the saturation solubility limit.

## 3 Basics and Implications of Kinetic Isotope Effects

### 3.1 Fundamentals

The classic definition of KIE is the ratio of reaction rates with and without isotopic substitution in the reactant(s) (Equation 2). The substitution of isotopes with higher mass would reduce the vibrational frequency, thus resulting in lower ground‐state vibrational energy (or zero‐point energy, ZPE) (Equations (3), (4), (5)).^[^ ^74^ ^]^

(2) $$\mathrm{KIE}=\frac{K}{k_{\mathrm{isotope}}}$$

(3) $$\varepsilon_{0}=\frac{1}{2}h\nu$$

(4) $$\nu =\frac{1}{2\pi }\sqrt{\frac{k}{m}}$$

(5) $$m=\frac{m_{1}m_{2}}{m_{1}+m_{2}}$$

where ε_0_ is the ZPE, *h* is the Planck constant, n is the vibrational frequency, k is the force constant of a bond, and m is the reduced mass for two atoms (m_1_ and m_2_) in a bond. Notably, Equation 4 is also known as Hooke's law, which is commonly used when calculating the isotope shift in infrared and Raman spectra (details in Section 4.5 Combination with Other Characterizations and DFT Calculation). Since the electronic energy remains unchanged after isotope substitution, the difference in reaction rates is dominantly determined by the difference in ZPE. If the reduction in ZPE after isotope substitution in the reactants cannot fully offset that in the transition state, the reaction rate will change according to the transition state theory (Equation 6)^[^ ^23^ ^]^:

(6) $$k=\frac{k_{B}T}{h}\frac{Q^{*}}{Q_{A}Q_{B}}\exp\left(-\frac{\Delta \varepsilon_{0}}{k_{B}T}\right)$$

where *k* is the rate constant, *k*_B_ is the Boltzmann constant, T is the temperature, and Q*, *Q*_A_, and *Q*_B_ are partition functions of the transition state, reactant A, and reactant B, respectively. Δε_0_ is the difference in ZPE between the reactant and the transition state.

In addition to the direct comparison of reaction rates, KIE can be experimentally determined from (1) the current densities of voltammetry in H_2_O vs D_2_O according to Equation 7 ^[^ ^75^, ^76^ ^]^ or (2) the product formation rates. Yet, caution is needed if a single reactant can yield multiple products, as isotopically sensitive branching in product distribution may affect the apparent KIE.

(7) $$\mathrm{KIE}={\left(\frac{i_{\mathrm{cat},\;H_{2}O}}{i_{\mathrm{cat},\;D_{2}O}}\right)}^{2}$$

where, $i_{\mathrm{cat},\;H_{2}O}$ is the current density of the catalytic reaction in H_2_O and $i_{\mathrm{cat},\;D_{2}O}$ is the current density of the catalytic reaction in D_2_O. Notably, the above non‐competitive or parallel approach for KIE measurements can be imprecise, especially if small variations in temperature or other parameters occur, and often leads to higher error bars than competitive techniques—particularly for heavier elements. A more precise protocol is to react light‐ and heavy‐labeled substrates together in a single flask, minimizing discrepancies in reaction conditions. According to the labeling position, these KIEs could be further classified into intermolecular KIE and intramolecular KIE, which are conducted in the presence of both light‐isotope‐labeled and heavy‐isotope‐labeled substrates, or in the presence of light and heavy isotope co‐labeled substrates, respectively (Equation 8).^[^ ^77^ ^]^ An illustration for intermolecular and intramolecular KIE is also provided in Figure S1.

(8) $$\mathrm{KIE}=\;\frac{k_{\mathrm{light}}}{k_{\mathrm{heavy}}}$$

where *k*_light_ is the reaction rate of the light‐isotope‐labeled substrate and *k*_heavy_ is the reaction rate of the heavy‐isotope‐labeled substrate.

Although ^1^H/^2^H (protium/deuterium) is the most commonly discussed isotope effect, heavier elements also exhibit KIE, such as ^12^C/^13^C KIE, ^14^N/^15^N KIE, ^16^O/^18^O KIE, ^35^Cl/^37^Cl KIE, and others. In the following sections, we would first examine ^1^H/^2^H KIE using commercially available isotope‐enriched compounds, and then focus on heavy atom KIE and how multiple isotope effects (e.g., δ^13^C + δ^15^N) may be combined to elucidate complex reaction mechanisms.

#### 3.1.1 Magnitude of ^1^H/^2^H KIE in an Elementary Reaction

**Figure 4:**
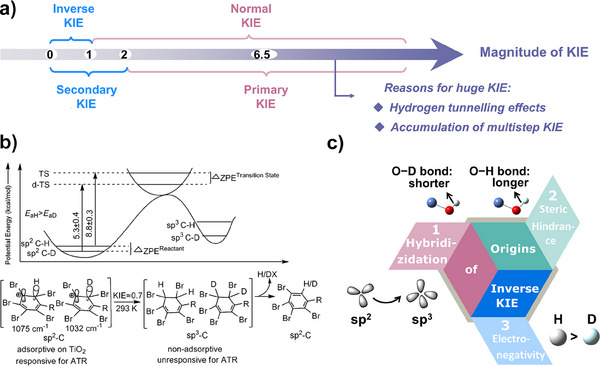
a) Illustration of the magnitude of different KIEs. b) Illustration of the origins of inverse KIE during the reductive degradation of decabromodiphenyl oxide. Copyright 2015 Wiley‐VCH.^[^ ^83^ ^]^ c) Three different sources of inverse KIE.

For an elementary reaction that involves the cleavage of C─H bond, the difference of ZPE between C─H and C─D bonds in the initial state is typically greater than that in the transition state because the strength of the C─H bond is weakened in the transition state, leading to lower‐energy molecular vibrations. As a result, the KIE is usually greater than 2, and in this case, the KIE is referred to as the primary KIE (Figure 4a). Assuming that the C─H or C─D bond is completely broken in the transition state, the maximum primary KIE is calculated as 6.5 in theory.^[^ ^23^ ^]^ However, in some cases, the observed KIE can exceed 6.5, which could be ascribed to either the quantum tunneling effects of hydrogen atoms, which penetrate rather than surmount the energy barrier of the transition state,^[^ ^78^, ^79^, ^80^ ^]^ or the accumulation of multiple KIE from several successive elementary reactions.^[^ ^81^ ^]^ Because tunneling effects are strongly dependent on atom mass, the substitution of H by D would significantly slow down the reaction rate and result in a colossal KIE. Conversely, if the C─H or C─D bond is not broken in the elementary reaction, the KIE would range between 0 and 2, because the configuration of the transition state may be influenced by the isotope atoms. This type of KIE, arising from the isotope substitution in the vicinity of the broken bond, is referred to as the secondary KIE. Notably, the cleavage of C─H bond of nonpolar compounds in aqueous solutions often induces a cage effect, where no primary KIE can be found (details in Text S1).^[^ ^82^ ^]^

Based on the relative reactivity of deuterated and protonated substrates, KIE could be classified into normal KIE (≥ 1) and inverse KIE (< 1). The inverse KIE manifests that the deuterio substrate has a higher reaction rate than the protio counterpart. Notably, the inverse KIE also belongs to the secondary KIE because no cleavage of the C–H bond is involved. A common cause of inverse KIE is the transformation of carbon hybridization (sp^2^ to sp^3^, or sp to sp^2^). The change in hybridization from Csp^2^ to Csp^3^ mainly affects the out‐of‐plane bending vibration of a C─H bond, which is stiffer for the Csp^3^ hybridization (1350 cm^−1^) than for the Csp^2^ hybridization (800 cm^−1^).^[^ ^26^ ^]^ In the transition state, the percentage of Csp^3^ hybridization is higher than that in the reactants. Therefore, the ZPE difference between the deuterio and protio reactants in the initial state is smaller than that in the transition state, resulting in an inverse KIE.

For example, Chang et al.^[^ ^83^ ^]^ found that the hydrophobic decabromodiphenyl oxide is first protonated and then adsorbed on the surface of TiO_2_ to capture one electron, which becomes hydrogenated at the ortho‐position (Figure 4b). Due to the transformation of the aromatic ring from Csp^2^ to Csp^3^ hybridization, the reaction exhibits a KIE of only 0.7. Beyond the hybridization change, many other factors might also lead to an inverse KIE. As shown in Figure 4c, the O─H bond is 3% longer than that of O─D bond, leading to greater steric hindrance on the surface with abundant hydroxyl groups.^[^ ^76^ ^]^ Besides, D is more electropositive than H, allowing D to donate electrons more easily. As a result, OD^−^ is more polar than OH^−^ and exhibits a stronger affinity for positively charged metal sites.^[^ ^84^ ^]^

#### 3.1.2 Composition of the Observed (total) ^1^H/^2^H KIE

**Figure 5:**
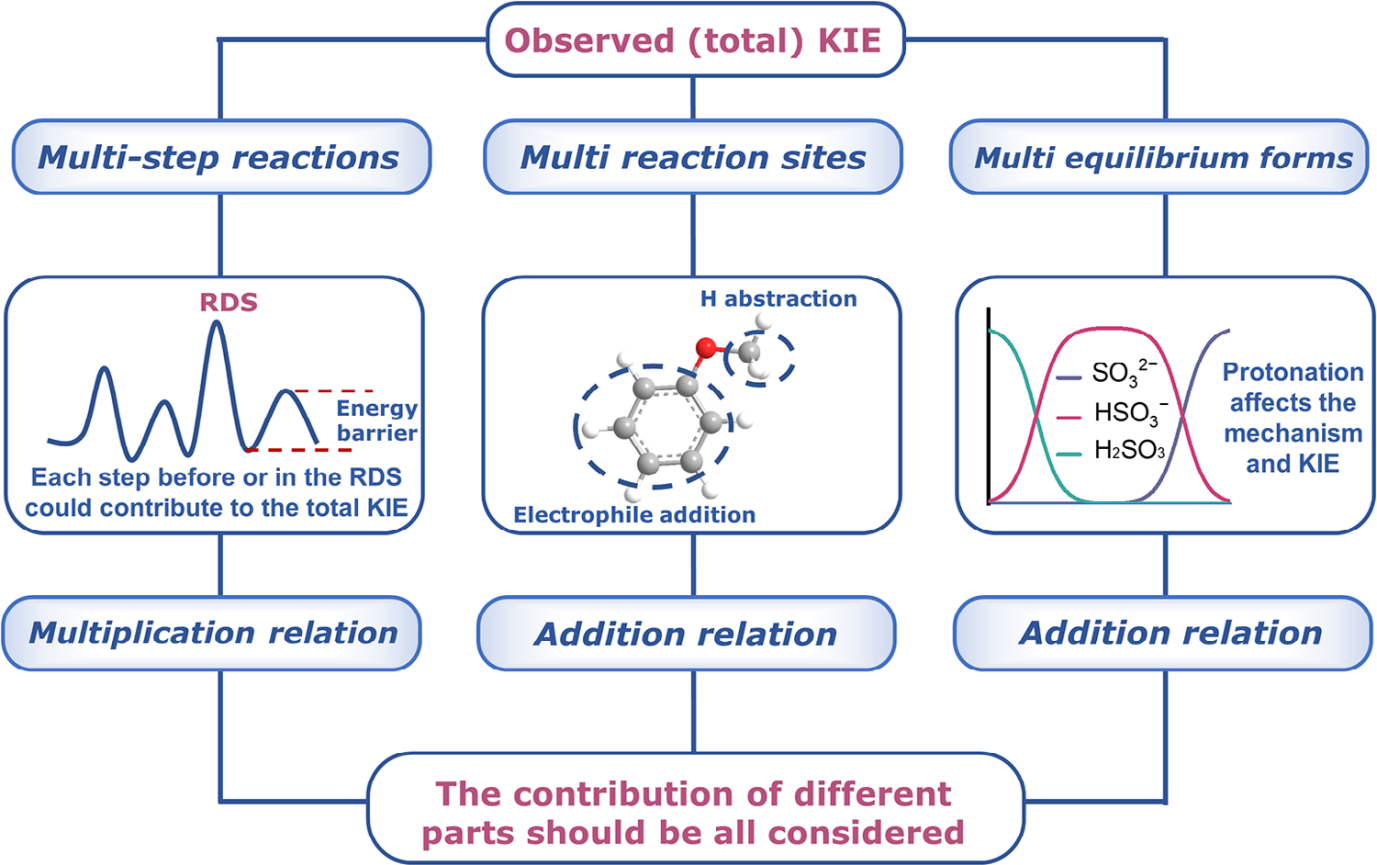
Illustration of the composition of the observed (total) KIE.

In an actual reaction, which usually involves multiple steps, reaction sites, and equilibrium forms of reactants, the observed KIE is a collective reflection of each elementary KIE (Figure 5). First, for a multistep reaction, the observed KIE usually includes the contributions from all the individual KIEs before and during the rate‐determining step (RDS).^[^ ^77^, ^85^, ^86^ ^]^ Especially, when the reaction in the RDS involves the cleavage of the C─H or O─H bond, a pronounced KIE would be observed. For example, He et al.^[^ ^85^ ^]^ used DFT calculations to investigate the change of the relative energy barrier during the electrochemical oxygen evolution reaction (OER) on FeNi and FeNiMo catalysts. They found that the OER on FeNi catalysts is limited by the desorption of *OO to form oxygen vacancy, whereas on FeNiMo catalysts, it is limited by the deprotonation of *OOH. As a result, a much higher KIE is observed for FeNiMo catalysts since the cleavage of the O─H bond occurs in the RDS. Therefore, if a reaction is featured with a remarkable KIE, it can be inferred that the cleavage of the X─H (X = C, O, or N) bond occurs in the RDS.^[^ ^87^ ^]^ Conversely, a minor KIE may indicate either a secondary KIE or that the cleavage of the X─H bond occurs before or after the RDS.^[^ ^88^ ^]^

The observed KIE also encompasses multiple individual KIEs at different reaction sites in one specific substrate. Especially, ^•^OH can attack different functional groups in the organic molecular via radical adduction, hydrogen abstract, and electron transfer pathways with a non‐selective characteristic. For nonpolar compounds like propane, the reaction between propane and ^•^OH could be divided into the reactions at the methyl site and the methylene site. Despite the higher reactivity of the methylene group, the reaction at this site only accounts for 72% of the total reaction, resulting in a total KIE of 3.21 if the whole molecule is deuterated (KIE_methyl_ = 4.74 and KIE_methylene_ = 2.61).^[^ ^89^, ^90^ ^]^

For polar compounds like alcohol, reactions with ^•^OH dominantly occur at the α─C─H bond rather than the polar O─H bond.^[^ ^30^, ^91^, ^92^ ^]^ However, for CH_3_NH_2_, the proportion of N─H reacting with ^•^OH (63%) is remarkably higher than that of C─H (37%). Therefore, only the deuteration of the aliphatic–NH_2_ group would cause significant effects on total KIE. Notably, the deuteration of the C─H bond would not significantly change the site‐selectivity, as the deuteration would not effectively reduce the activation energy difference between C─H bond and O(N)─H bond. Due to the different KIE values of different reaction sites, the observed KIE of the total reaction might be much lower than expected for a primary KIE (Tables S5–S6).

For aromatic compounds, both the alkyl site and the conjugated π system can be attacked by ^•^OH and HMOS. The selectivity toward the two sites is determined by their relative energy barrier, the reactant adsorption configuration, and the reaction conditions, such as solution pH.^[^ ^36^, ^93^ ^]^ A primary ^1^H/^2^H KIE is generally observed for the alkyl site, whereas an inverse ^1^H/^2^H KIE is observed for the addition of ^•^OH and HMOS on benzene rings due to the change of hybridization from sp^2^ to sp^3^.^[^ ^93^, ^94^ ^]^

The observed KIE is also composed of the individual KIEs of the different equilibrium species. Especially, the substrates with different protonation and deprotonation states should be treated as separate components, with their respective KIEs calculated individually. For example, both SO_3_^2−^ and HSO_3_^−^ are the equilibrium species of sulfite at weak acidic conditions.^[^ ^95^ ^]^ Thus, the KIE of the reaction between the high‐valent Ru(VI) complex and sulfite should be divided into two species‐specific KIEs.^[^ ^96^ ^]^ Apparently, the reaction between Ru(VI) and HSO_3_^−^ in D_2_O results in primary KIE (as high as 17.4), while the reaction with SO_3_^2−^ produces a much lower KIE (only 1.04).

### 3.2 Implication of ^1^H/^2^H KIE

#### 3.2.1 Distinguishing Different ROS or Oxidation Mechanism

One important implication of KIE is to identify reactive species or reaction mechanisms, as different reactive species or different reaction mechanisms exhibit different KIE patterns. Generally, the higher the selectivity of the ROS, the greater the KIE observed for organic degradation.^[^ ^94^, ^97^ ^]^ For example, Sühnholz et al.^[^ ^98^ ^]^ argued that SO_4_^•−^, rather than ^•^OH, is the dominant ROS generated in FeS/PDS system because the KIE for cyclohexane degradation (2.22) is much higher than ^•^OH‐dominated systems (1.08). Pignatello et al.^[^ ^99^ ^]^ found that after the addition of tert‐butanol in the UV/Fe^3+^/H_2_O_2_ system, the KIE for cyclohexane degradation increased from 1.2 to 1.4, suggesting the possible generation of Fe(IV) in addition to ^•^OH.

According to the distinctive attack pathway of ^1^O_2_ on phenol rings^[^ ^100^ ^]^ (1,4‐addition and subsequent C─H bond cleavage), Luo et al.^[^ ^101^ ^]^ used phenol‐d_5_ to investigate the ROS generated in the electrode‐periodate system. The observed primary KIE of 2.37 suggested that the dominant ROS was ^1^O_2_, as ^•^OH and other reactive species, such as aqueous Fe(IV), typically induce an inverse KIE when reacting with phenol in aqueous solution due to the change of carbon hybridization.

#### 3.2.2 Distinguishing Different Proton‐Coupled Electron Transfer Mechanism

**Figure 6:**
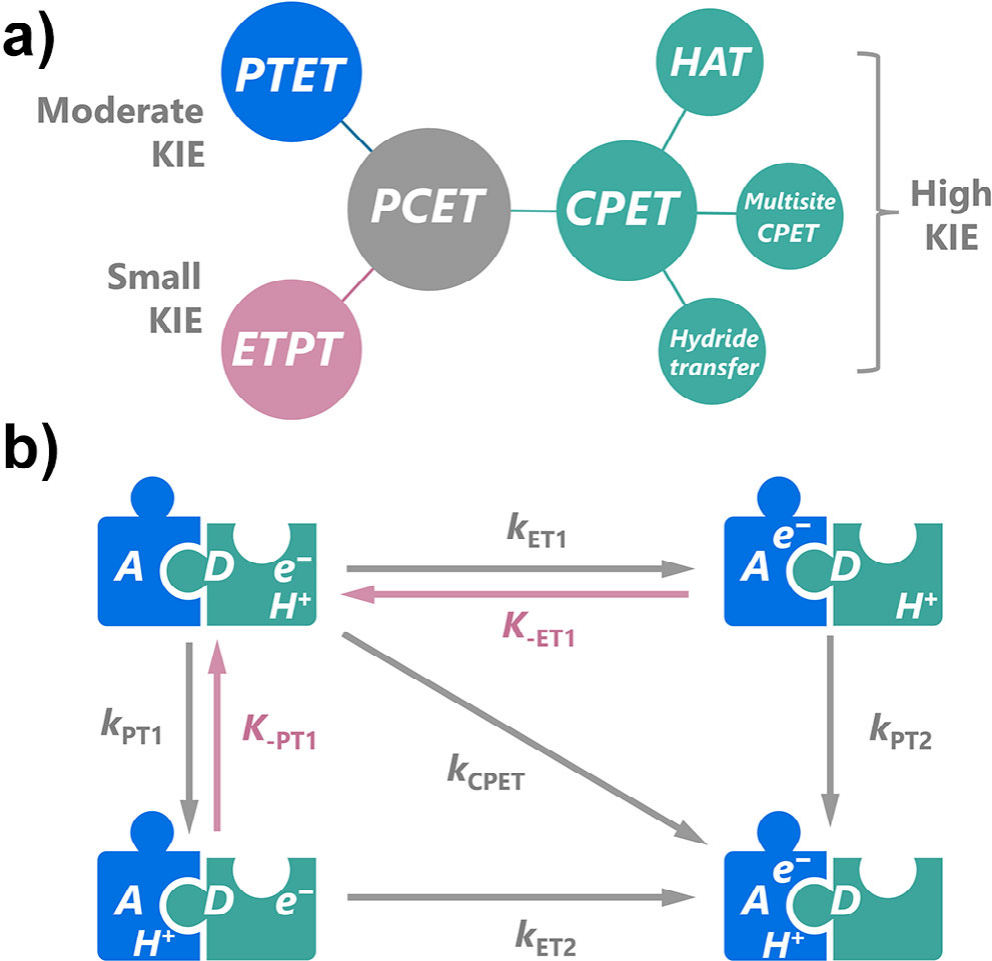
Illustration of the composition of the observed (total) KIE.

The degradation of organic contaminants usually involves the cleavage of the X─H bond (X = C, N, O, etc.), which is a typical proton‐coupled electron transfer reaction (PCET). Based on the sequence of the two elementary steps, i.e., the proton transfer (PT) step and the electron transfer (ET) step, the PCET process can be categorized into electron transfer proton transfer (ETPT, ET occurs before PT), proton transfer electron transfer (PTET, PT occurs before ET) and concerted proton electron transfer (CPET, PT, and ET occur simultaneously) (Figure 6). Compared with the solo ET step, the combination of ET with PT imparts an energetic advantage by lowering the activation barrier and the required driving force of the reaction,^[^ ^102^ ^]^ thus elevating the overall reaction rates. In terms of CPET, the reactions could be further divided into hydrogen atom transfer (HAT), multisite CPET, and hydride transfer. Specifically, if the source and sink of the proton and electron are the same, it is referred to as HAT. However, if the proton and electron originate from different substrates or the acceptors of the proton and electron are different, it is referred to as multisite CPET. As for hydride transfer, it resembles the HAT process, but two electrons are transferred from the reducing agents to the oxidants.

For the unknown reactions, comparing the KIE values is a well‐established approach to distinguishing the type of PCET and determining the driving force of the reactions.^[^ ^103^, ^104^, ^105^ ^]^ A large observed KIE generally corresponds to a CPET process, while a moderate and small KIE might be ascribed to a PTET or ETPT process, respectively. However, in some cases, the PTET process might also induce a great KIE, as the proton tunneling effects could also happen in the PTET process.^[^ ^102^ ^]^ Zhang et al.^[^ ^106^ ^]^ investigated the oxidation of HCOO^−^ at Pt anode and found that the oxidation proceeds via two pathways with the same RDS, namely, the adsorption of HCOO^−^ onto the surface of Pt (Equations (9), (10)):

(9)

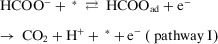



(10)

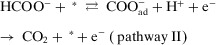




In the first pathway, the reaction was initiated by an ET step to form HCOO_ad_ intermediates (Equation 9), and then followed by a CPET process to produce CO_2_. Because the RDS does not involve deprotonation, pathway I exhibits a KIE close to unity. However, pathway II begins with a CPET process to form ${\;\mathrm{COO}}_{\mathrm{ad}}^{-}$ intermediate, followed by an ET process to produce CO_2_. Therefore, a significant KIE is expected in pathway II due to the involvement of deprotonation in the RDS. With the increase of pH from 1 to 13, the KIE would decrease from 5 to 1, suggesting that the oxidation gradually changed from pathway II to pathway I. Moreover, comparing KIE values across different para‐substituted analogs can assist in the identification of the reaction mechanism. For example, Shearer et al.^[^ ^107^ ^]^ established a quantitative‐structure‐activity relationship between Hammett constants and the oxidation rates of para‐substituted N,N‐dimethylaniline by a Cu(I)‐dioxygen adduct. The negligible change in KIE with the increase of Hammett constants indicated that the reaction proceed via an ETPT process, while the RDS is the ET process from N,N‐dimethylaniline to the adduct.

As a recently developed novel reaction mechanism, the direct electron‐transfer oxidation mechanism (an ET or ETPT process) could also be distinguished from the ROS‐based mechanism due to the small theoretical KIE.^[^ ^3^, ^32^, ^108^ ^]^ However, in some cases, the KIE in ROS‐based mechanism might also approach to unity, depending on the types of ROS, contaminants, and solvent. In acidic aqueous conditions, Cr(IV) oxidizes phenol through hydrogen abstraction from the phenolic O─H bond (large KIE), while (bpy)_2_(py)Ru^IV^=O^2+^ proceeds via the electrophilic attack on the aromatic ring (small and even inverse KIE).^[^ ^63^ ^]^ Fe(IV)‐ligand complex can oxidize phenol via either hydrogen abstraction^[^ ^109^ ^]^ or electrophilic attack on the aromatic ring.^[^ ^110^, ^111^ ^]^

The solvent also plays a critical role in determining the mechanism. For instance, phenol oxidation would prefer to occur via ET rather than HAT in polar solvents, while the HAT mechanism becomes dominant in nonpolar solvents.^[^ ^103^ ^]^ Therefore, in some cases, a small KIE could not distinguish the direct electron transfer mechanism from the ROS‐based mechanism. Notably, D_2_O should be used as the solvent if phenol‐d_6_^[^ ^31^, ^108^, ^112^ ^]^ or aniline‐d_7_^[^ ^32^, ^113^ ^]^ is used as the target contaminants as rapid H/D exchange would occur between active proton and the solvent. To avoid the interference of D_2_O on the other elementary steps, control experiment should be conducted using other contaminants that would not involve proton transfer during oxidation.

#### 3.2.3 ^1^H/^2^H KIE Measurements in Photocatalytic Reactions

Photocatalytic reactions involve two half‐reactions: the reduction reaction induced by the photogenerated electrons at the conduction band (CB) and the oxidation reaction induced by the photogenerated holes at the valence band (VB). As a result, both reactions at CB and VB would contribute to the overall observed KIE. In terms of CB reaction, the one‐electron reduction of H_3_O^+^ and the subsequent H_2_ evolution would be greatly impeded by D_2_O with a KIE of 3.5 when perylenetetracarboxylic acid nanosheets are used as the catalysts.^[^ ^114^ ^]^ Similarly, the reduction of O_2_ into H_2_O_2_ by K01/C TiO_2_ photocatalysts involves the cleavage of O─H bond, and thus, a KIE of 1.5 is observed.^[^ ^115^ ^]^ As for the VB side, the oxidation of D_2_O into ^•^OH involves the cleavage of O─H bond; then, a primary KIE will be observed.

In some cases, the KIE is induced by the reaction at the opposite band. For example, the reduction of Ag^+^ is hindered when the substrate, benzyl alcohol, in the VB reaction is replaced by the benzyl alcohol‐d_2_.^[^ ^116^ ^]^ This indicates that the CB reaction is constrained by the VB reaction, making the oxidation of benzyl alcohol to release electrons become the RDS of the total reaction. Choi et al.^[^ ^117^ ^]^ found that the KIE for the reductive dehalogenation of CCl_4_ in the presence of electron donors (like CD_3_OH/CD_3_OD and (CH_3_)_2_CHOH/(CD_3_)_2_CDOD) is 2 at pH 11, but close to unity at pH 2.8. The difference of KIE at different pH is attributed to the change of RDS during the photocatalytic reaction. According to the Nernstian equation, the redox potential of CB electron and VB hole would decrease as pH increases. Therefore, the reduction ability of CB electrons is insufficient at acidic pH, making the reduction half‐reaction RDS. Conversely, at basic pH, the oxidation ability of VB hole decreases, thus rendering the oxidation of alcohols the RDS.

#### 3.2.4 ^1^H/^2^H KIE Measurements in Electrochemical Reactions

**Figure 7:**
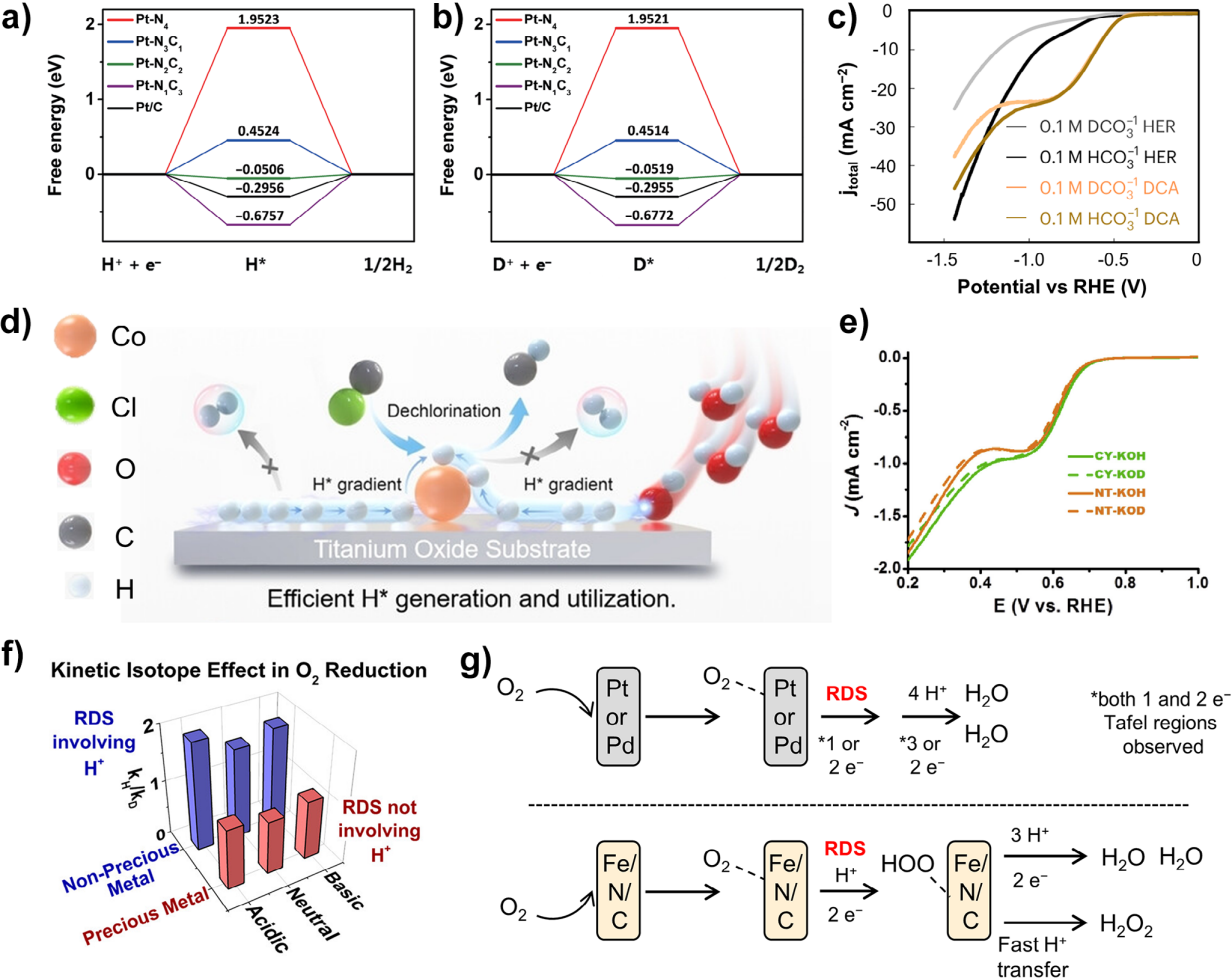
a) Calculated ΔG_H*_ and b) ΔG_D*_ values for different Pt‐NxC_4_‐x structures at the equilibrium potential. Copyright 2022 Springer.^[^ ^119^ ^]^ c) LSV curves of electrochemical dichlorination and HER in D_2_O solution. Copyright 2022 Springer Nature.^[^ ^120^ ^]^ d) Scheme of the H*‐dominated dichlorination. Copyright 2024 Wiley‐VCH.^[^ ^121^ ^]^ e) LSV curves of catalysts carried out in 0.1 M KOH and KOD media. Copyright 2022 Elsevier.^[^ ^122^ ^]^ f) KIE of precious metal catalysts and nonprecious metal catalysts in ORR. g) Illustration of the RDS for the ORR by two catalysts. Copyright 2016 American Chemical Society.^[^ ^123^ ^]^

Some key reactions occurring at the cathode during electrochemical reactions include hydrogen evolution reactions (HER), electrochemical dehalogenation reactions, electrochemical nitrate reduction reactions, 2‐e^−^ oxygen reduction reactions (ORR) to produce H_2_O_2_ and 4‐e^−^ ORR to produce H_2_O. In the HER process, the proton‐discharge step to form adsorbed H* from H_3_O^+^ involves the cleavage of O─H bond and the formation of the metal–H bond, resulting in a normal KIE.^[^ ^118^ ^]^ Notably, the magnitude of KIE in HER is greatly influenced by the catalyst type. As shown in Figure 7a,b, different Pt‐N‐C single‐atom catalysts with various coordination environments present different free energies for the formation of adsorbed H*.^[^ ^119^ ^]^ Among these, Pt‐N_2_C_2_ shows the lowest activation energy and highest relative change after deuterium replacement. Therefore, the KIE of Pt‐N_2_C_2_ during HER reaches as high as 5.13, while Pt nanoparticles only attain a KIE of 2.76.

As for electrochemical dehalogenation and nitrate reduction reaction, the current mechanisms are classified into two types, direct electron‐transfer and H*‐dominated reduction.^[^ ^124^ ^]^ It was found that the dichlorination of 1,2‐dichloroethane on Co‐N_4_ sites proceeded via a direct electron transfer pathway without significant KIE (Figure 7c).^[^ ^120^ ^]^ In contrast, the dichlorination at atomically dispersed Co─O sites on TiO_x_ support and commercial Pd/C exhibited KIEs of 1.66 and 1.83, respectively, suggesting that these processes were dominated by the H* reduction mechanism (Figure 7d).

In terms of 2‐e^−^ and 4‐e^−^ ORR, the KIE of both reactions is determined by whether the proton is involved in the RDS.^[^ ^125^, ^126^ ^]^ Generally, two elementary reactions are considered the most common RDS in ORR: the formation of O_2_^−*^ from O_2_ (ads) coupled with the first ET (that is, O_2_ (ads) + e^−^ → O_2_^−*^) and the protonation process of OOH* from O_2_^−*^ (that is, O_2_^−*^ + H_2_O → OOH* + OH^−^).^[^ ^122^ ^]^ As shown in Figure 7e, the production of H_2_O_2_ by two nanocarbons under alkaline conditions is hardly influenced by the isotope substitution, indicating that the first ET to form O_2_^−*^ is the RDS for these two catalysts. Similarly, Tse et al.^[^ ^123^ ^]^ found that no significant isotope effects were observed for precious metal ORR catalysts, while a KIE of about 2 was observed for a Fe/N/C catalyst (Figure 7f–g). Therefore, the RDS during the ORR of precious metal catalyst does not involve the cleavage of the O–H bond in H_3_O^+^, whereas the RDS for Fe/N/C catalysts does.

**Figure 8:**
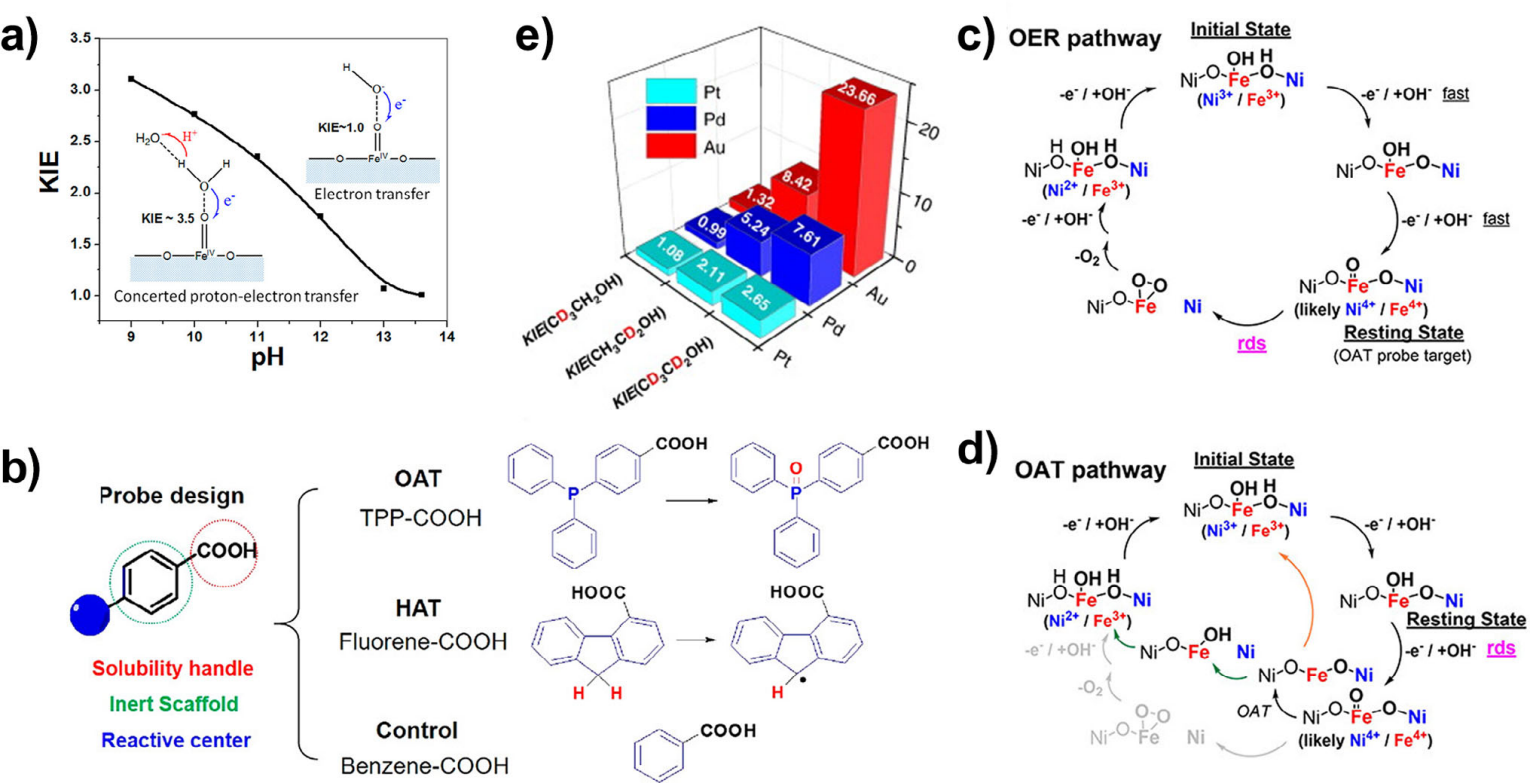
a) The change of KIE as a function of the solution pH during photoelectrochemical water oxidation on hematite photoanodes. Copyright 2016 American Chemical Society.^[^ ^128^ ^]^ b) Design of the probe to selectively screen the specific elementary reaction. The proposed mechanism of the OER catalytic cycle c) without and d) with the OAT probe addition. Copyright 2021 American Chemical Society.^[^ ^129^ ^]^ e) KIE of the anodic oxidation of ethanol on Pt, Pd, and Au. Copyright 2013 Elsevier.^[^ ^130^ ^]^

Compared with cathodic reactions, the oxygen evolution reaction (OER) at the anode is more kinetically hindered with a high overpotential. If the RDS involves the formation of active oxygen species (*O) through the deprotonation process of *OH or the release of O_2_ from the deprotonation of *OOH, a significant primary KIE is typically observed.^[^ ^127^ ^]^ For example, the photoelectrochemical water oxidation on hematite photoanodes exhibits a KIE of 3.5 at pH 9 and a KIE of 1 at pH 13.^[^ ^128^ ^]^ At pH 9, the surface Fe=O moiety is predominantly attacked by molecular H_2_O, while at higher pH the Fe=O moiety is attacked by OH^−^ (Figure 8a). The concerted electron transfer from molecular water to a surface‐trapped hole, coupled with proton transfer to another solvent water molecule, represents a typical CPET process. Thus, a higher KIE is observed at a lower pH.

Hao et al.^[^ ^129^ ^]^ designed a chemical probe of 4‐(diphenylphosphino) benzoic acid to selectively screen the surface intermediate HMOS during the OER process and assist in identifying the RDS (Figure 8b). Upon the addition of the probe, the KIE would be elevated from 1.37 to 1.64, suggesting the shift of a secondary KIE toward a primary KIE that involves H‐related bond breaking as the limiting step. Specifically, the RDS shifted from the nucleophilic attack of OH^−^ on surface Fe(IV) = O to the deprotonation of Fe–OH (Figure 8c,d).

In addition to OER, electrochemical anodic oxidation also plays a pivotal role in the decontamination of organic pollutants. Ren et al.^[^ ^130^ ^]^ investigated the oxidation of alcohols on Pt, Pd, and Au anodes. As shown in Figure 8e, the deuteration of β−H has little influence on the overall oxidation rate, while the deuteration of α−H significantly slowed down the oxidation rate with the KIEs of 2.1, 5.2, and 8.4 for Pt, Pd, and Au, respectively. Intriguingly, the remarkably lower KIE for Pt is ascribed to its high affinity to hydrogen. Accordingly, the cleavage of α−H from C–H on Pt is effortless, and one of the further steps may become the RDS.

### 3.3 Kinetic Solvent Isotope Effects (KSIE)

#### 3.3.1 ^1^O_2_−Induced ^1^H/^2^H KSIE

^1^O_2_ is an attractive ROS due to its high selectivity in organic oxidation and the high tolerance toward the co‐existing factors in the practical water matrix.^[^ ^131^ ^]^ Traditionally, ^1^O_2_ is generated via the excitation of dissolved organic matter (DOM) or molecule sensitizer and their subsequent energy transfer to ^3^O_2_ in sunlit surface water,^[^ ^132^, ^133^, ^134^ ^]^ and through chemical reactions between HOCl and H_2_O_2_.^[^ ^135^ ^]^ Recently, numerous work has reported the catalytic production of ^1^O_2_ from PMS,^[^ ^136^, ^137^, ^138^, ^139^ ^]^ PDS,^[^ ^140^, ^141^ ^]^ PAA,^[^ ^142^ ^]^ PI,^[^ ^143^, ^144^ ^]^ and ozone.^[^ ^145^, ^146^ ^]^ The dominant methods for detecting ^1^O_2_ include EPR measurement,^[^ ^14^ ^]^ quenching experiments,^[^ ^9^, ^14^ ^]^ or chemical capturing using probes like 9,10‐diphenylanthracene.^[^ ^131^, ^147^ ^]^ However, the presence of ^1^O_2_ is often debated due to the uncertainties in these methods and the potential contribution from other mechanisms that could result in a false‐positive signal.^[^ ^3^, ^11^ ^]^

In contrast, an intriguing characteristic of ^1^O_2_ is its significantly longer lifetime in D_2_O compared with H_2_O (68 ± 1 µs vs 3.7 ± 0.4 µs). This difference is due to the weaker physical deactivation rate (*k*_d_) of ^1^O_2_ by D_2_O (2.5 × 10^5^ s^−1^ in H_2_O vs 1.6 × 10^4^ s^−1^ in D_2_O).^[^ ^148^ ^]^ Therefore, if the EPR signal or the degradation rate of the contaminant obviously increases, it can be speculated that ^1^O_2_ may be produced and contribute to the system. A particularly useful metric for assessing ^1^O_2_ ​ involvement is the ratio of reaction rates in D_2_O vs H_2_O, known as kinetic solvent isotope effects (KSIE) and defined as *k*_D2O_/*k*_H2O_. (Note that for non‐^1^O_2‐_induced KSIE, it is customarily defined as *k*_H2O_/*k*_D2O_).

**Figure 9:**
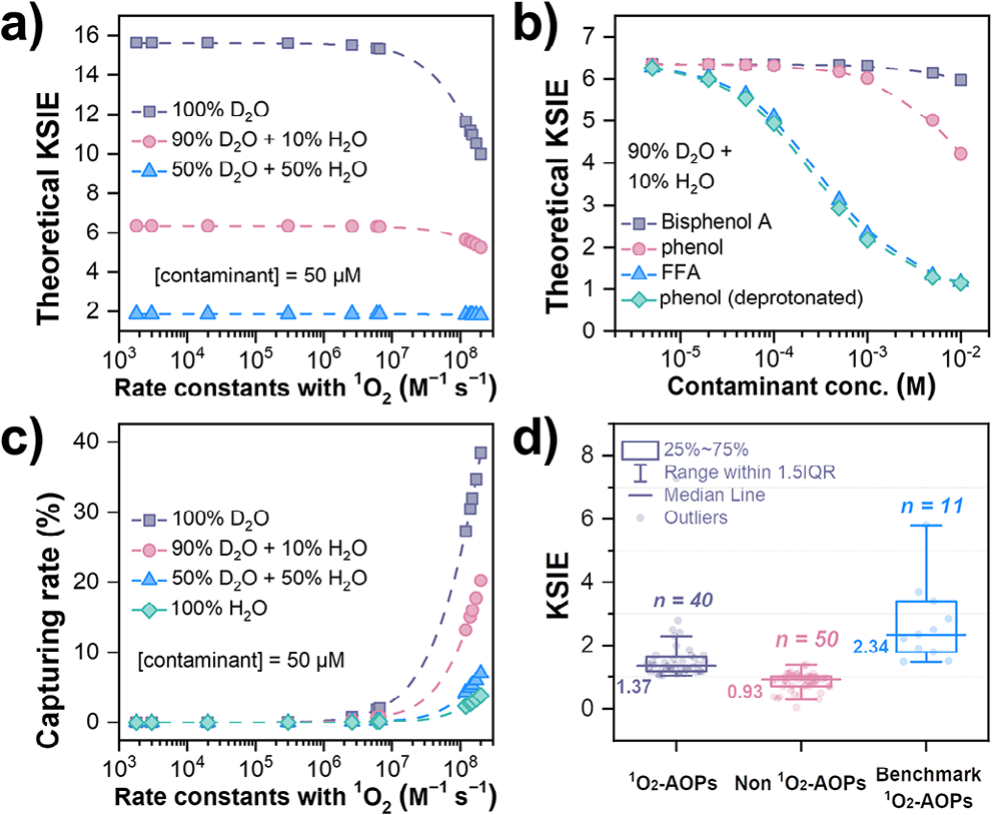
a) Change of theoretical KSIE as a function of the rate constants of the organic contaminants with ^1^O_2_. b) Change of theoretical KSIE as a function of the contaminant concentration. c) Change of the capturing rate of ^1^O_2_ by organic contaminants as a function of the rate constants of the organic contaminants with ^1^O_2_. d) Statistic results of the KSIE values reported in different references about different AOP systems.



To comprehend the factors influencing KSIE, we plotted the theoretical KSIE profile as a function of the second‐order reaction rates of contaminants with ^1^O_2_ (Figure 9a,b, Text S1, and Table S7). Clearly, KSIE will be elevated significantly at lower contaminant concentrations, higher D_2_O percentages, and lower secondary rate constants of contaminants. However, a low secondary rate constant can inversely cause the low capturing rate of ^1^O_2_ by organics (Equation 11), as ^1^O_2_ would be severely quenched by the solvent in this case (Figure 9c).^[^ ^149^ ^]^ The low capturing rate suggests that even though ^1^O_2_ is generated in the system, it would not effectively contribute to contaminant degradation. Therefore, to observe a discernible KSIE, contaminants like furfuryl alcohol are recommended for such studies.

(11)

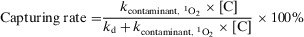




**Figure 10:**
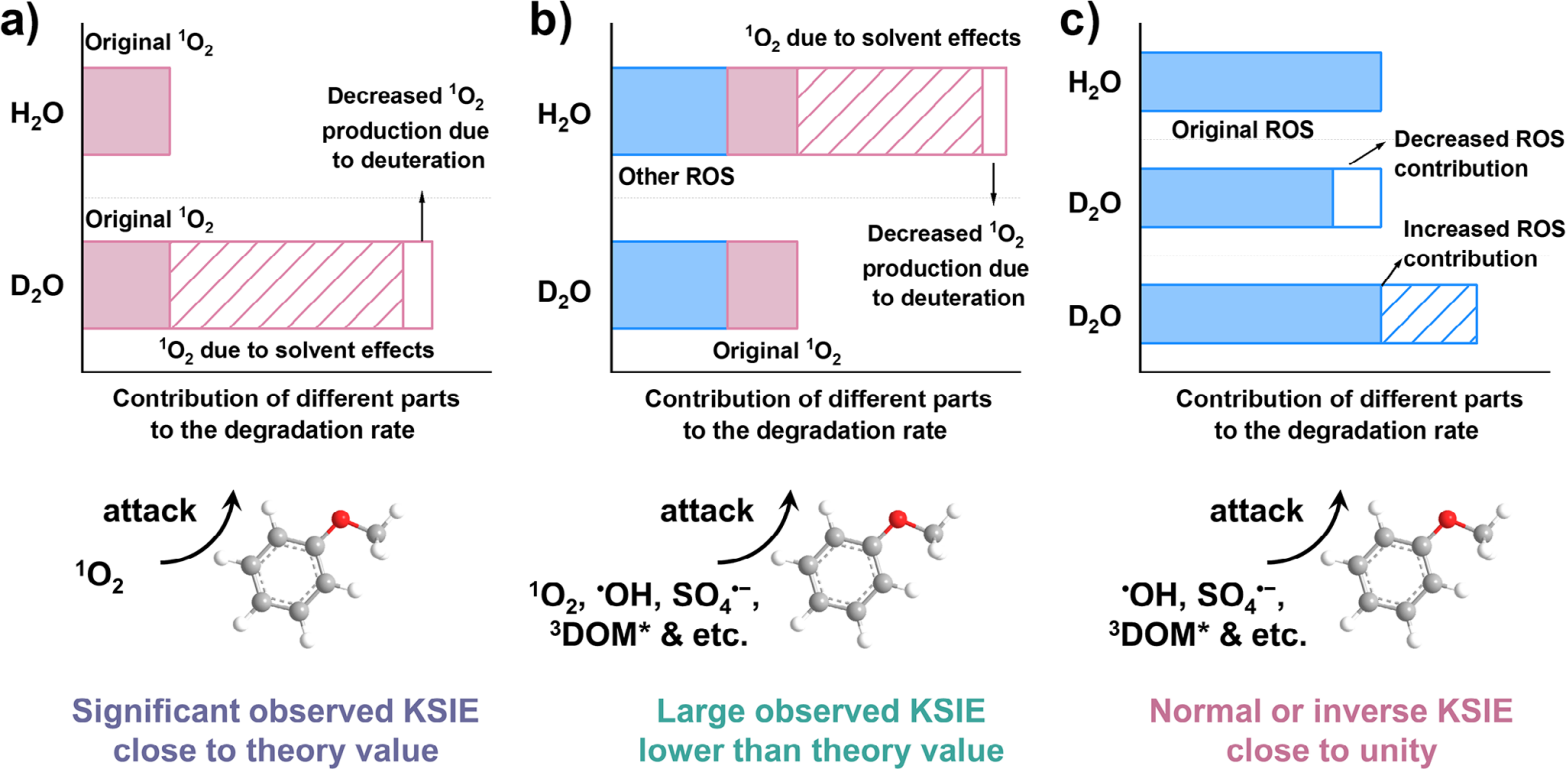
The effects of D_2_O on the change of the degradation rate in an AOP system a) with ^1^O_2_ as the only oxidant, b) with ^1^O_2_, ^•^OH, SO_4_^•−^, 3DOM*, and other oxidation mechanisms all contributing to contaminant degradation, and c) without the participation of ^1^O_2_.

We further summarized the KSIE in many AOP systems, including alleged ^1^O_2_‐involved AOPs, non‐^1^O_2_‐involved AOPs, and benchmark ^1^O_2_‐generated AOPs (Figure 9d and Tables S8–S10). The statistic median KSIE values of these three systems are 1.37, 0.93, and 2.34, respectively. The slightly lower KSIE in non‐^1^O_2_‐involved AOP systems can be ascribed to the effects of D_2_O on ROS generation. In contrast, the obviously lower KSIE in alleged ^1^O_2_‐AOPs compared with benchmark systems can be explained by the following two primary reasons: (1) the systems are dominant by multiple ROS, and thus, the elevated amount of ^1^O_2_ in D_2_O might not cause a high observed KSIE; or (2) the elevated performance in D_2_O is not ascribed to ^1^O_2_. As shown in Figure 10a, for the benchmark ^1^O_2_ systems like light/Rose Bengal, light/C_60_, and light/DOM, the dominant ROS are only ^1^O_2_, sometimes accompanied by the excited triplet sensitizers like ^3^DOM*. In this case, the observed KSIE is slightly lower than the theoretical value because of the deuteration effects on the second‐order reaction rates of organic with ^1^O_2_ and the production rate of ^1^O_2_. In terms of systems involving multiple ROS, the KSIE is much lower than the theoretical value owing to the concurrently increased contribution in denominator and numerator by other ROS (Figure 10b). Finally, in the non‐^1^O_2_‐involved AOP systems, the degradation rate usually decreases because of the higher activation energy caused by in situ deuteration. However, an inverse KSIE may occasionally be observed, attributed to the easier generation and transformation of ROS.^[^ ^65^, ^150^ ^]^

To ensure a reliable analysis of KSIE, we recommend a systematic approach. First, the easily distinguishable ROS like ^•^OH, SO_4_^•−^, and O_2_^•−^ should be excluded using low‐impact quenchers like ethanol and superoxide dismutase. The remaining *k*_obs_ of organic degradation can then be used to recalculate the KSIE. Notably, this method may introduce complications if ^1^O_2_ originates from the transformation of such ROS, like the oxidation of O_2_^•−^ by SO_4_^•−^.^[^ ^151^ ^]^ More importantly, we suggest supplementing experiments with furfuryl alcohol (FFA) at a concentration of no higher than 50 µM. FFA has a relatively higher capturing rate for ^1^O_2_ and a moderate theoretical KSIE, making it a reliable reference that would provide more trustworthy evidence for the identification of ^1^O_2_. Finally, an accurate statement of D_2_O concentration in the experimental method is essential for a correct analysis of KSIE.

#### 3.3.2 Non ^1^O_2_−Induced ^1^H/^2^H KSIE

As discussed above, the displacement of H_2_O by D_2_O will induce multiple effects on the systems in addition to prolonging the lifetime of ^1^O_2_. One of the most common effects is caused by the deuteration of exchangeable hydrogen atoms in the contaminant. For other ROS like HMOS and excited triplet molecules, the in situ deuteration can be detrimental to the CPET process on phenolic moiety.^[^ ^152^ ^]^ Similarly, the deuteration can also influence the reaction between PI and H_2_O_2_ because of the presence of secondary isotope effects.^[^ ^48^ ^]^ In catalytic reactions, the surface hydroxyl group of the catalysts would also be deuterated to produce ≡OD, which creates weaker bonding with the adsorbate and reduces adsorption strengths.^[^ ^153^ ^]^ Besides, Shao et al.^[^ ^154^ ^]^ also found that the solvent exchange to D_2_O results in a slower PMS decomposition rate.

**Figure 11:**
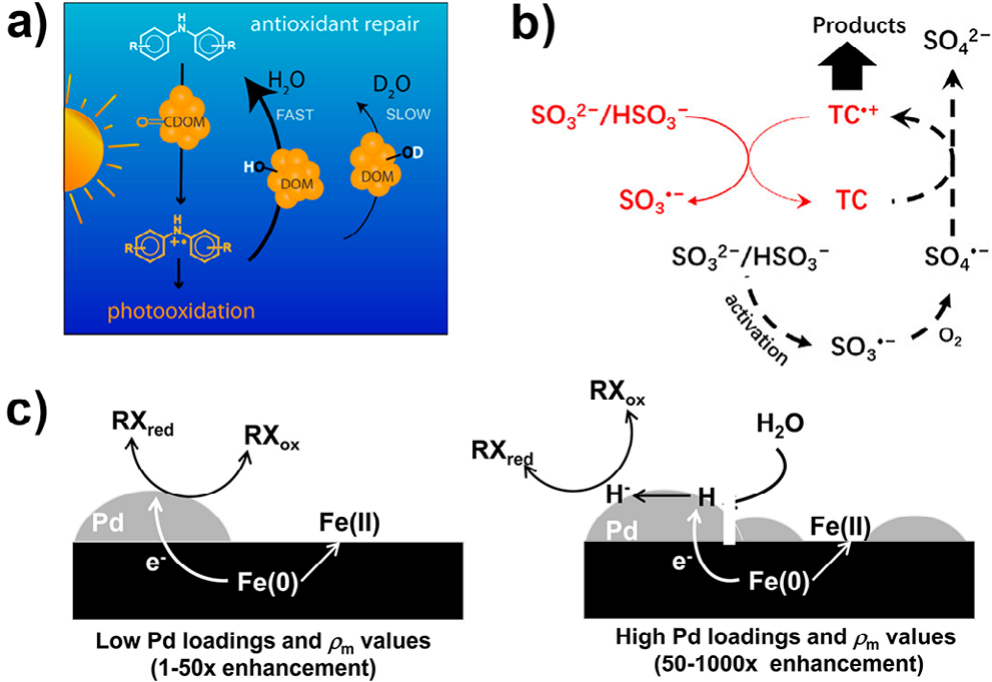
a) Illustration of the slower reduction rate of the oxidation intermediate by the antioxidant moiety of DOM. Copyright 2018 American Chemical Society.^[^ ^65^ ^]^ b) Illustration of the reduction of the oxidation intermediate by sulfite in UV/sulfite AOP system. Copyright 2022 American Chemical Society.^[^ ^155^ ^]^ c) Comparison of the dechlorination mechanism by nZVI and Pd‐loaded nZVI. Copyright 2013 American Chemical Society.^[^ ^81^ ^]^

In some cases, deuteration effects can induce inverse KSIE. For example, during the degradation of diclofenac in the light/DOM system, diclofenac is first oxidized to a N‐centered radical cation, which would subsequently experience the oxidative cleavage of C─N bond or the reduction to diclofenac by the phenolic moieties in the DOM (Figure 11a,b). This regeneration process occurs via a CPET process. Herein, the solvent exchange to D_2_O can suppress this backward reaction (with a phenolic O─D bond) and promote the overall conversion of diclofenac.^[^ ^65^ ^]^ This regeneration phenomenon is common in AOP systems involving reducing agents, such as the UV/sulfite system, where the tetracycline radical cation could be reduced by the sulfite.^[^ ^155^, ^156^ ^]^

In addition to the deuteration solvent effects, water can directly participate in the reaction. As a result, any reaction that involves the cleavage of an O─H bond in H_2_O and H_3_O^+^ can contribute to the KSIE. For example, due to the “slower” decomposition reaction rate with solvent (KSIE = *k*_H_/*k*_D_) of 2.85), the aqueous Fe(IV) also possesses a longer lifetime in D_2_O.^[^ ^111^ ^]^ Similarly, under acidic conditions, the hydrolysis of SO_4_^•−^ to ^•^OH involves O─H bond cleavage in H_2_O. Since the DO─D bond binding energy (5.28 ± 0.20 eV) is higher than that of HO─H (5.1 eV),^[^ ^157^ ^]^ the solvent exchange with D_2_O will decrease the yield of ^•^OH.^[^ ^158^ ^]^ In contrast, at neutral conditions, the hydrolysis of SO_4_^•−^ is dominated by OH^−^. Therefore, no effects on RS steady concentration were observed.^[^ ^159^ ^]^

In the dehalogenation of *cis*‐dichloroethene (cisDCE), Xie et al.^[^ ^81^ ^]^ found that the nano zero‐valent iron (nZVI) with a small Pd loading exhibits a small KSIE in D_2_O (Figure 11c). This suggests that nZVI with small Pd loading proceeded via the direct electron transfer mechanism, where Pd enhanced the utilization efficiency of electrons released from the corrosion of the Fe core to facilitate cisDCE reduction. In contrast, nZVI with a high Pd loading showed a significantly higher KSIE of 100, suggesting the involvement of solvent in the dichlorination process. Specifically, atomic H* probably serves as the reactive species for cisDCE reduction. The exceptionally high KSIE can be ascribed to the cumulative effects of multiple reaction steps involving the formation of atomic H(D) via solvent reduction, potential uptake of H(D) into the Pd lattice to promote hydride (deuteride) formation, and subsequent reaction of the atomic H with cisDCE. In contrast, the sulfidation of nZVI to form a surface sulfide coating only enhances the electron transfer from Fe to the chlorinated contaminant without the production of active H and the corresponding KSIE.^[^ ^160^ ^]^ Notably, the formation of dissolved Fe^2+^ is indeed slowed down in D_2_O, this is because the reaction between Fe^0^ and D^+^ is slower than that between Fe^0^ and H^+^, rather than the decreased electron transfer rate between the chlorinated contaminant and Fe^0^.

In some cases, D_2_O exhibits a higher intrinsic reactivity compared with H_2_O. As discussed in Section 3.1.1., D is less electronegative than H. Accordingly, OD^−^ is more nucleophilic than OH^−^, which explains why the decomposition of persulfate in alkaline conditions is accelerated in D_2_O.^[^ ^161^ ^]^ Finally, D_2_O can influence other properties like redox potential, secondary reaction rate constants, and hydrogen bonds, which are discussed in detail in Text S3 and Figure S2.^[^ ^76^, ^162^, ^163^ ^]^

### 3.4 Heavy Atom KIE and Compound‐specific Isotope Analysis

Due to the limited source of deuterated substances, direct comparisons of reaction rates and calculations of ^1^H/^2^H KIE are largely restricted to commercially available compounds or compounds with exchangeable hydrogen sites. In contrast, the calculation of ^1^H/^2^H KIE and heavy atom KIE (e.g., ^12^C/^13^C KIE, ^14^N/^15^N KIE, ^16^O/^18^O KIE, ^35^Cl/^37^Cl KIE) at natural isotope abundance offers a great opportunity to monitor the reactions of diverse substances of interest.^[^ ^164^ ^]^

Given the small differences in rate constants, which are similar in magnitude to the influence of other factors, such as temperature, these KIE measurements are not dependent on the comparison of absolute rate constants, but on the change of relative isotope ratio at determined time points.^[^ ^27^ ^]^ Notably, the time points can span from minutes to decades, allowing researchers to trace the reaction over extensive time scales. To determine such isotope ratios, one should typically analyze samples using gas/liquid chromatography combined with isotope ratio mass spectrometer (GC/LC‐IRMS) or NMR techniques.^[^ ^165^, ^166^ ^]^ In a typical GC‐IRMS procedure, the molecules of concern are converted into CO_2_, N_2_, and H_2_ before analysis, providing an average rather than position‐specific information about the isotope ratio shift.^[^ ^167^ ^]^ Therefore, such KIE measurement is often referred to as compound‐specific isotope analysis (CSIA). For the same element, when additional atoms not involved in the reaction are present, the observed magnitude of KIE values may decrease.

In general, isotope ratios (*R*) are expressed relative to the respective international reference material (Equation 12). For example, the ^13^C/^12^C ratio is reported in terms of C isotope signatures, δ^13^C, in δ notation.^[^ ^27^ ^]^

(12)

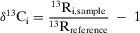

where ^13^R_i,sample_ and ^13^R_reference_ are the ^13^C/^12^C ratios determined for the carbon atoms in analyte i in a sample and the reference material (Vienna Pee Dee Belemnite), respectively. Based on the Rayleigh equation and nonlinear regression analysis (Equation 13), the estimate of a KIE can be calculated through Equation 14.

(13)

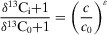

(14) $$\mathrm{KIE}=\frac{1}{1+n\;\times \;\varepsilon }$$

c and c_0_ are the concentration initially and at sampling time, respectively, ε is the isotope enrichment factor, and n is the total number of atoms of the element considered. Most heavy atom KIEs are primary and small in magnitude (typically between 0.95 and 1.05), but they can be normal or inverse.^[^ ^166^ ^]^ For a normal KIE (KIE > 1), the lighter substrate reacts faster than the heavier substrate, resulting in progressive enrichment of the heavy isotope in the remaining substrate and enrichment of the light isotope in the products. However, when the reaction approaches to completion, the isotope ratio in the generated products would finally approach the initial value of the reactants.

Such heavy atom KIEs and CSIA have shown great potential in discerning the reaction mechanisms and pathways in various water treatment processes, such as in the reduction of nitroaromatic compounds,^[^ ^168^ ^]^ dehalogenation of trichloroethene by zero‐valent iron,^[^ ^169^ ^]^ oxidation of benzene, toluene, ethylbenzene, xylenes (BTEX) by MnO_4_,^−[^ ^170^ ^]^ and heterogeneous photo‐Fenton reactions.^[^ ^171^ ^]^ When analyzing an unknown reaction, its isotope ratios, enrichment factors, or KIE values are usually compared with some benchmark systems or theoretical calculation values to get insights into the dominant mechanisms. For example, Spahr et al. found that the chloramination of secondary and tertiary amines involves the participation of O_2,_ and the corresponding ^18^O‐KIE was found to be between 1.0026 ± 0.0003 and 1.0092 ± 0.0009.^[^ ^19^ ^]^ Such a KIE is far smaller than those of one‐electron or two‐electron reduction of O_2_ to produce O_2_^•−^ (1.03) or O_2_^2−^ (1.05), but comparable to the reversible binding of O_2_ to oxygen transport proteins (1.004–1.005). Therefore, the reaction was attributed to the involvement of the binding of O_2_ by an unidentified radical intermediate, rather than a conventional electron‐transfer mechanism.

Meyer et al. investigated the oxidative N‐dealkylation of atrazine by cytochrome P450 and observed large normal C (up to 1.019) and H (up to 3.1) isotope effects.^[^ ^167^ ^]^ The measured ^13^C‐KIE was comparable to the theoretical results for a HAT mechanism (1.0065–1.0087), but much higher than that for a single electron transfer mechanism (0.9965). Consequently, the authors concluded that atrazine was oxidized by cytochrome P450 via Fe(IV)‐mediated HAT pathway.

**Figure 12:**
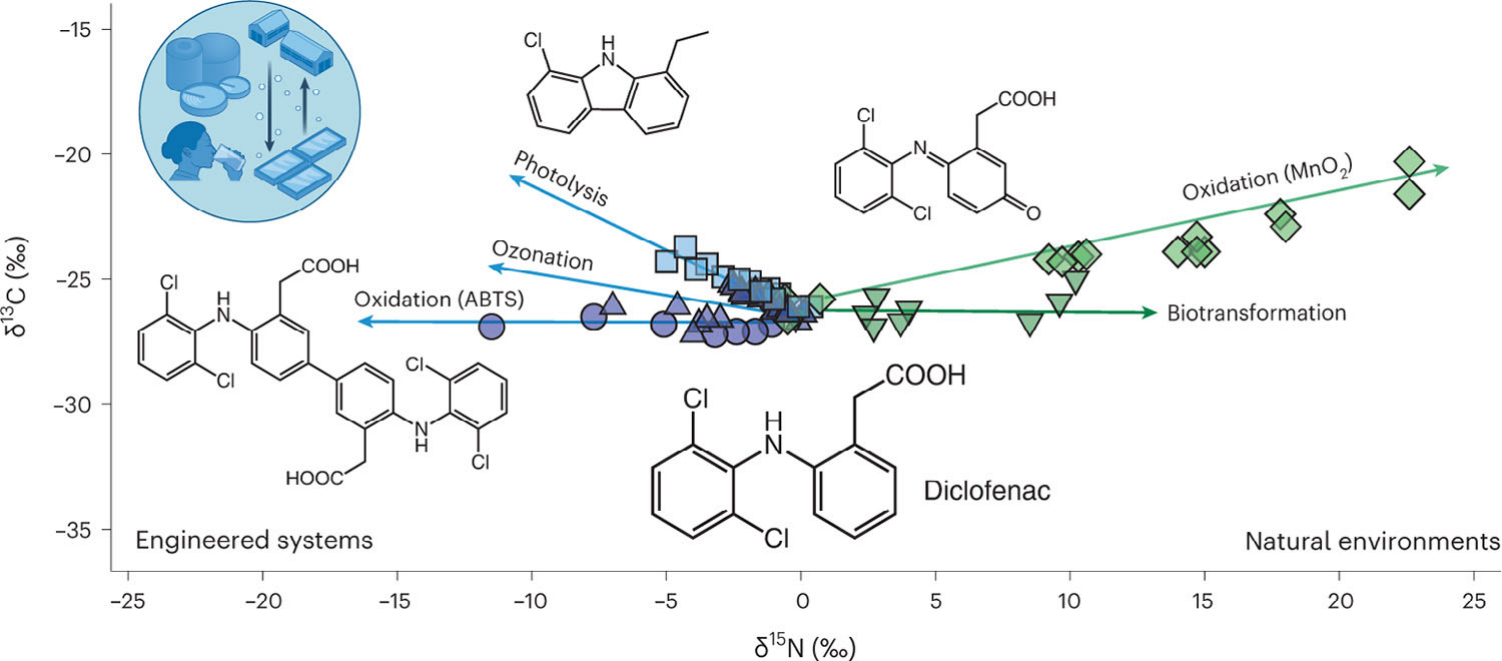
Diclofenac elimination pathways in engineered systems (blue: photolysis, ozonation, and oxidation) and in natural environments (green: mineral‐catalyzed oxidation and biodegradation) are characterized by distinct patterns of carbon and nitrogen isotope fractionation. Copyright 2024, Springer Nature.^[^ ^27^ ^]^

Furthermore, dual‐element (or multi‐element) isotope plots can further enhance mechanistic interpretation. As shown in Figure 12, the combination of ^13^C and ^15^N CSIA categorized the elimination of diclofenac into engineered systems and natural systems.^[^ ^27^, ^172^ ^]^ Both systems exhibited the normal ^13^C KIE, but only the natural systems exhibited the normal ^15^N KIE. Such a distinctive pattern enables us to predict and differentiate the degradation pathways of any unknown systems.

### 3.5 Roadmap of Interpreting KIE in Sophisticated Systems

**Scheme 1:**
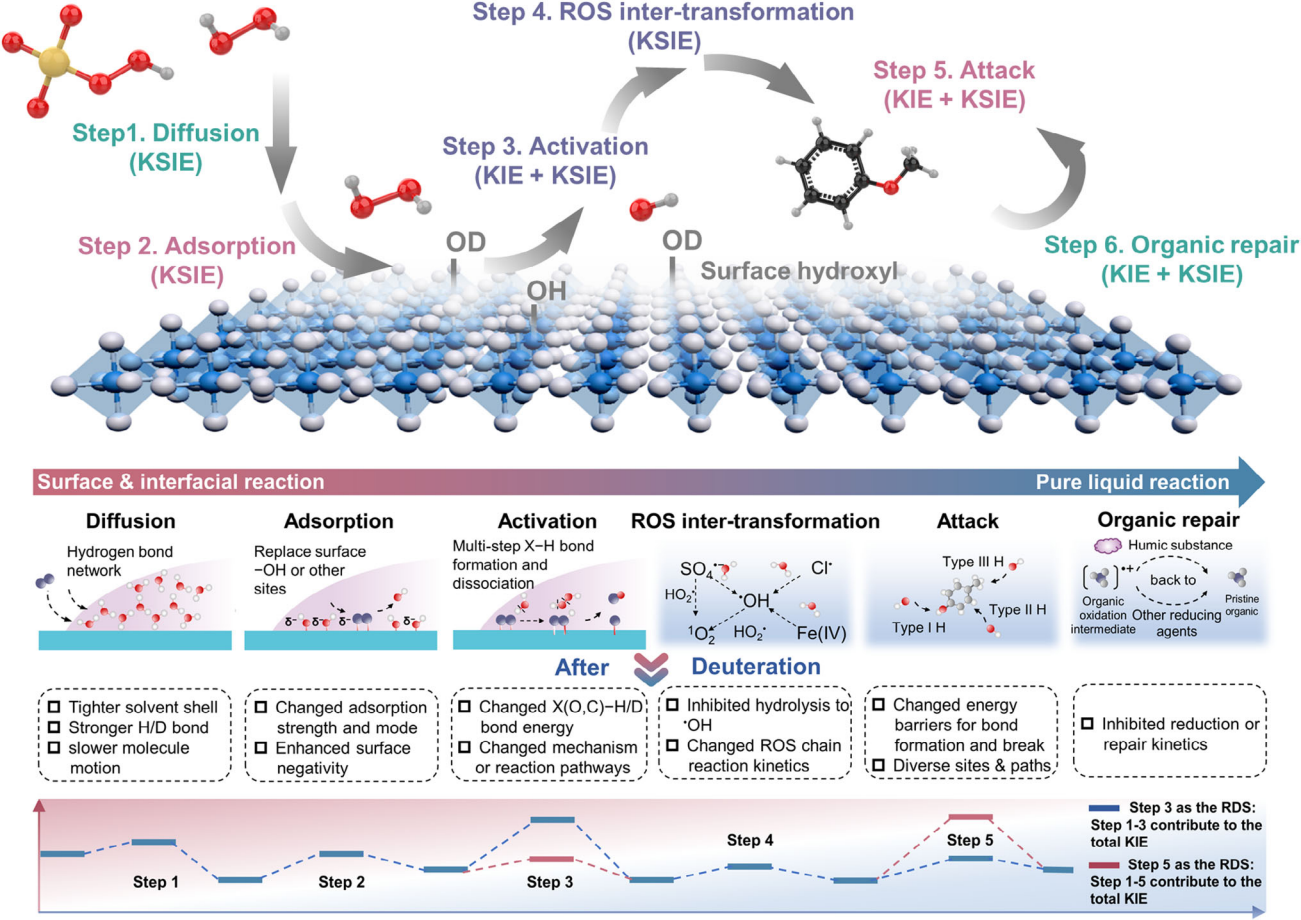
Every step that might produce KIE or KSIE in a typical heterogeneous catalytic oxidation process.

One should be cautious in interpreting data during an isotope substitution experiment because every step during ROS generation and contaminant transformation is likely to cause a KIE or KSIE. Therefore, we plotted a roadmap for considering every step that might produce KIE or KSIE in a typical heterogeneous catalytic oxidation (Scheme 1). If an isotope‐substituted organic is used as the substrate, only the attack of organics by ROS and the potential back‐conversion of short‐lived organic radical intermediates to the parent contaminant might induce a KIE. However, if D_2_O is used in lieu of H_2_O, multiple steps could induce a KSIE. This includes the diffusion, adsorption, the activation of oxidants, the inter‐transformation of different ROS, the attack of ROS on the organics, and the repair of oxidized organic radicals. In some cases, the change of the reaction rate is ascribed to the different reactivities of D_2_O with substrates, while in other cases, changes in reaction rates are attributed to the in situ deuteration of easily exchangeable groups. Notably, step 4 is usually overlooked but critical in comprehending the underlying observed isotope effects. The gap between ROS generated in step 3 and ROS that attacks organics in step 5 may account for the observed isotope effects.^[^ ^173^ ^]^

Herein, to exclude the possibility of KIE and KSIE from other steps, we recommend that careful control experiments with different organic contaminants should be carried out. Through a careful comparison of the degradation of contaminants with different functional groups, the role of steps 1–4 can be well excluded, thus increasing the reliability of the conclusion drawn.

## 4 Basics and Implications of Isotope Labeling

### 4.1 Analysis of the Origins of Oxygen

Oxygen is critical and constitutes various ROS and organic functional groups. Therefore, an accurate analysis of oxygen transformation is essential for understanding the overall reaction mechanisms. In most cases, ^18^O_2_, H_2_^18^O, and other ^18^O‐labeled substrates are individually or collectively utilized to trace oxygen from different sources. The calibration of the natural ^18^O percentage in the final products is required when quantitatively calculating the contribution of different oxygen sources, especially when H_2_^18^O is diluted with H_2_^16^O solution (details in Text S4). Besides, the isotope distributions can be greatly influenced if the molecules contain multiple atoms with naturally abundant stable isotopes of+2 Da (e.g., sulfur, with 4.2% natural abundance of ^34^S).^[^ ^44^ ^]^ In these cases, the target screening range in extracted ion chromatograms in the mass spectrometer should be carefully narrowed to ensure the analysis accuracy.^[^ ^174^, ^175^ ^]^

#### 4.1.1 Analysis of the Origins of ^•^OH and the Presence of HMOS

**Figure 13:**
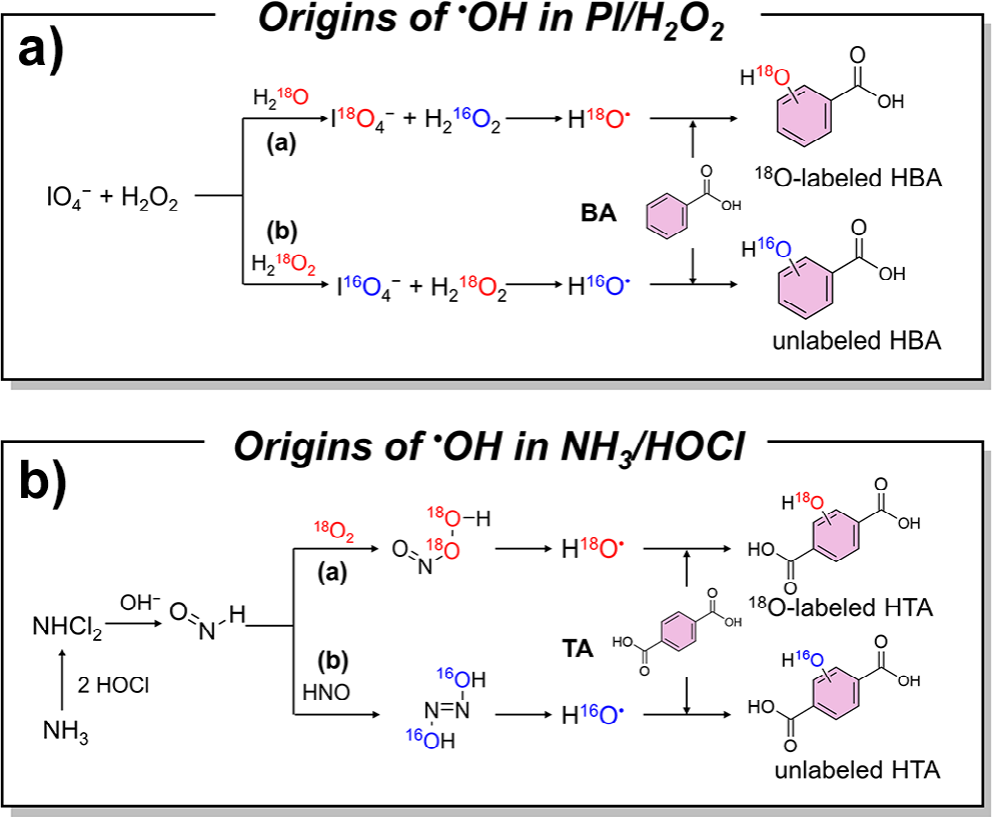
Schematic illustration of the origins of ^•^OH in a) PI/H_2_O_2_ and b) NH_3_/HOCl system using ^18^O‐labeling techniques.

In addition to the well‐documented Fenton reaction (Fe^2+^/H_2_O_2_) and UV/H_2_O_2_ process, many innovative systems have emerged that generate ^•^OH as the dominant ROS like PI/H_2_O_2_ system, halogenated quinones/H_2_O_2_ systems.^[^ ^47^, ^48^, ^176^, ^177^ ^]^ However, the generation pathways of these systems are initially unclear. Kim et al.^[^ ^48^ ^]^ first reinvestigated the chemistry behind the generation of ^•^OH from PI/H_2_O_2_ interaction (Figure 13a). Using BA as the probes of ^•^OH and H_2_^18^O_2_ as the marker, they demonstrated that no ^18^O‐labeled hydroxybenzoic acid (HBA) was generated from this system, confirming ^•^OH does not originate from the cleavage of peroxide bond. Chen et al.^[^ ^47^ ^]^ further used H_2_^18^O to in situ label the IO_4_^−^ and PMSO_2_ to capture ^•^OH, which exclusively generated ^18^O‐labeled hydroxylated PMSO_2_ as the products. These findings collectively proved that the oxygen comes from PI. Similarly, Lu et al.^[^ ^49^ ^]^ used ^18^O‐labeling experiment to prove that ^•^OH generated in breakpoint chlorination originated from the decomposition of peroxynitrite (ONOOH) rather than that of H─O─N═N─O─H (Figure 13b).

Thereby, the accurate analysis of the origin of ^•^OH should meet one prerequisite: the stability of isotope abundance in both reactants and the products (^•^OH does not exchange O with H_2_O). For easily exchangeable substances like PI, sufficient time is required to achieve complete exchange with H_2_^18^O. Notably, though the carboxylic group in HBA is theoretically exchangeable, it cannot achieve a high exchange percentage in a short period.

In addition to ^•^OH, ^18^O‐labeling techniques have been employed for the identification of HMOS.^[^ ^178^, ^179^, ^180^, ^181^, ^182^, ^183^ ^]^ Using PMSO or DMSO as the probes, the presence of HMOS in a system can be confirmed by the simultaneous formation of ^16^O and ^18^O labeled PMSO_2_ (or DMSO_2_).^[^ ^184^ ^]^ The ratio of PMS^16^O^18^O to total PMSO_2_ (PMS^16^O^18^O+PMS^16^O^16^O) depends on the initial H_2_^18^O concentration (c_18O_), the specific sulfoxide concentration ([sulfoxide]), the exchange rates of HMOS with water (*k*_ex_), and the reaction rates between sulfoxide and HMOS (*k*_s_), as described by Equation 15.^[^ ^21^, ^185^ ^]^ Additionally, the side reaction between oxidant precursors (like PMS) and PMSO could also lead to the generation of PMSO_2_, thereby reducing the ^18^O ratio in PMSO_2_ or DMSO_2_, as PMS does not exchange oxygen atom with H_2_^18^O. For example, Zong et al.^[^ ^174^ ^]^ found that in Co(II)/PMS system, the ^18^O ratio increased from 11.9% to 61.3% when the [PMS]_0_: [Co(II)]_0_ ratio decreased from 10:1 to 1:10.

(15)

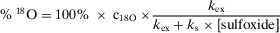




**Figure 14:**
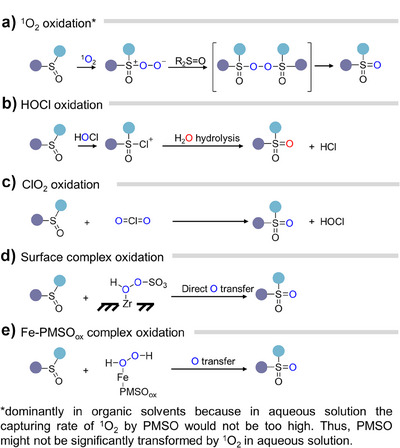
Schematic illustration of the generation pathways of sulfones by various ROS.

One key benefit of isotopes in HMOS investigation is its capacity to distinguish HMOS from other ROS that could also lead to the formation of PMSO_2_ like ^1^O_2_,^[^ ^131^ ^]^ HOCl,^[^ ^186^ ^]^ ClO_2_,^[^ ^187^ ^]^ surface or intermediate PMS complex^[^ ^188^, ^189^ ^]^ and the Fe(III)−PMSO oxidation products (Fe(III)−PMSO_ox_, Figure 14).^[^ ^190^, ^191^ ^]^ None of these ROS can simultaneously generate ^16^O‐and ^18^O‐labeled PMSO_2_ with an adjustable ratio by changing the H_2_^18^O concentration. Some single‐electron‐transfer oxidants, like CO_3_,^•−[^ ^192^ ^]^ and heat/peroxydisulfate(PDS) system,^[^ ^193^ ^]^ are also reported to oxidize PMSO into PMSO_2_. However, the specific oxidation mechanisms underlying these processes remain unclear, and the conversion efficiency for PMSO and selectivity for PMSO_2_ in some of these processes are not significant. Moreover, it is noteworthy that the generation and coexistence of secondary ROS from primary HMOS could cause some uncertainties. For example, the oxidation of PMSO by permanganate (Mn(VII)) can generate intermediate Mn(V),^[^ ^194^ ^]^ which can rapidly exchange oxygen atoms with water, oxidize PMSO, or disproportionate back to Mn(VII), leading to a higher ^18^O‐labeled ratio in PMSO_2_ than the ^18^O ratio of initial Mn(VII).^[^ ^175^ ^]^ Similar phenomenon might occur in Fe(VI)/Fe(V)/Fe(IV) and Co(IV)/Co(III) systems.^[^ ^195^ ^]^ Furthermore, in the co‐presence of diverse species like HOCl and ClO_2_, the identification of HMOS becomes more challenging.

**Figure 15:**
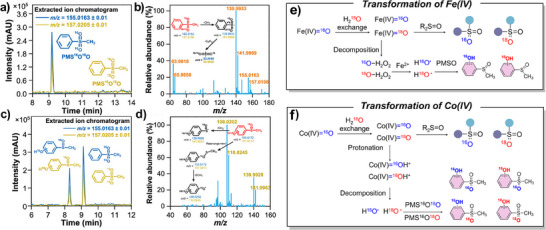
a) Extracted ion chromatography (EIC) of PMS^16^O^16^O and PMS^16^O^18^O generated during the oxidation of PMS^16^O in the Fe(II)/PI process in H_2_^18^O and b) the corresponding MS_2_ spectrum. c) EIC of PMS^16^O─^16^OH/PMS^16^O^16^O and PMS^16^O─^18^OH/PMS^16^O^18^O generated in the Fe(II)/PI process and d) the corresponding MS_2_ spectrum. Copyright 2021 American Chemical Society.^[^ ^52^ ^]^ Schematic illustration of the transformation of e) Fe(IV) and (f) Co(IV) in the aqueous solution and the formation of sulfones, hydroxylated sulfoxides, and hydroxylated sulfones.

^18^O‐labeling can also help distinguish the generation of ^•^OH from the decomposition of HMOS. This phenomenon is usually observed in Fe(IV) and Co(IV)−based systems, while no ^•^OH generation is expected from the decomposition of Mn(V)−NTA complexes (Figure 15a–d).^[^ ^52^, ^174^, ^196^ ^]^ In this system, ^•^OH acts as the secondary ROS, and its contribution is not dominant unless Fe(IV) and Co(IV) are inert to the substrates, such as nitrobenzene or dimethyl phthalate.^[^ ^174^ ^]^ As the decomposition products of HMOS, ^•^OH would inherit the ^16^O/^18^O ratio of HMOS. Zong et al. observed that the percentage of PMS^16^O─^18^OH in total PMSO─OH was 34.42% in the Fe(II)/PI system, which was close to the ratio of PMS^16^O^18^O to total PMSO_2_ (Figure 15e).^[^ ^52^ ^]^ This revealed that the ^•^OH generated in Fe(II)/PI system indeed comes from the decomposition of Fe(IV). Similar results were observed in Co(II)/PMS systems; specifically, the generation of double‐labeled PMS^16^O^18^O─^18^OH was ascribed to the oxygen‐transfer reaction with Co(IV)═^18^O and the hydroxylation reaction involving Co(IV)−derived H^18^O^•^ (Figure 15f).

#### 4.1.2 Analysis of the Origins of Oxygen in Contaminant or Oxidant Transformation

**Figure 16:**
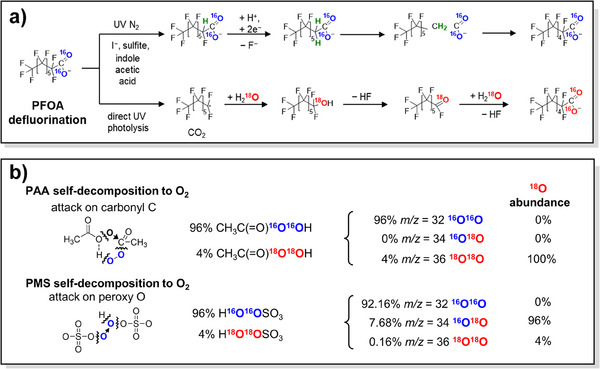
a) PFOA defluorination pathways confirmed via H_2_^18^O labeling b) Two different decomposition pathways of peroxides and the theoretical calculation of the m/z distribution for the generated O_2_.

In addition to discerning the origins of hydroxyl radicals, isotope labeling techniques can be applied to reveal the origins of oxygen‐containing functional groups in contaminants.^[^ ^197^, ^198^ ^]^ For example, the defluorination process of PFOA by UV/sulfite or UV/I^−^ process,^[^ ^199^, ^200^ ^]^ which generates hydrated e^−^ as the reducing agents, proceeds the successive hydrogeneration reactions and then decomposes into short‐chain fluorinated radicals, CH_2_ carbenes and CO_2_^•−^ radicals (Figure 16a). The fluorinated radicals and CO_2_^•−^ radicals would rapidly recombine to form perfluoroheptanoic acid.^[^ ^200^ ^]^ However, the direct photolysis of PFOA first proceeds the decarboxylation reaction to release CO_2_ and fluorinated radicals. These fluorinated radicals subsequently hydrolyze in H_2_^18^O, leading to the incorporation of ^18^O into the carboxylic groups.^[^ ^22^ ^]^

Isotopic experiments are effective for identifying the cleavage pathways of the O─O bond during the self‐decomposition of peroxides (Figure 16b). These pathways typically involve two mechanisms: (1) the terminal oxygen atom attacking another peroxy bond, and (2) the terminal oxygen atom attacking a heteroatom center. For example, during the decomposition of PMS, the terminal O in the O─O bond of SO_5_^2−^ anions attacks the O─O bond of another HSO_5_^−^.^[^ ^201^ ^]^ As a result, O_2_ is generated containing oxygen atoms from two different PMS molecules, yielding isotopic species ^16^O^16^O, ^16^O^18^O, and ^18^O^18^O with the *m/z* values of 32, 34, and 36, respectively. In contrast, both cleavage mechanisms contributed to the decomposition of PAA, as indicated by the 83% ^18^O abundance of ^18^O_2_ in the gaseous products, which is between 4% and 100% based on an initial ^18^O‐labeled PAA concentration of 4%.^[^ ^42^ ^]^

Jennings et al. used ^18^O‐labeled O_3_ to differentiate the direct oxidation products (oxidized by ^18^O_3_) and indirect oxidation products (oxidized by ^•^OH) from the ozonation of organic matters in the effluent.^[^ ^45^ ^]^ The direct oxidation products featured a higher ^18^O/^16^O ratio due to the direct oxygen transfer between organic matter and O_3_. In contrast, the ^18^O/^16^O ratio was much lower in indirect oxidation products because ^•^OH was generated from the decomposition of ^18^O_3_ in H_2_^16^O. Among the 929 oxidation products, only 84 were classified as direct oxidation products, suggesting the major contribution by indirect oxidation pathways.

#### 4.1.3 Analysis of the Origins of Oxygen in Photochemical and Electrochemical Reactions

**Figure 17:**
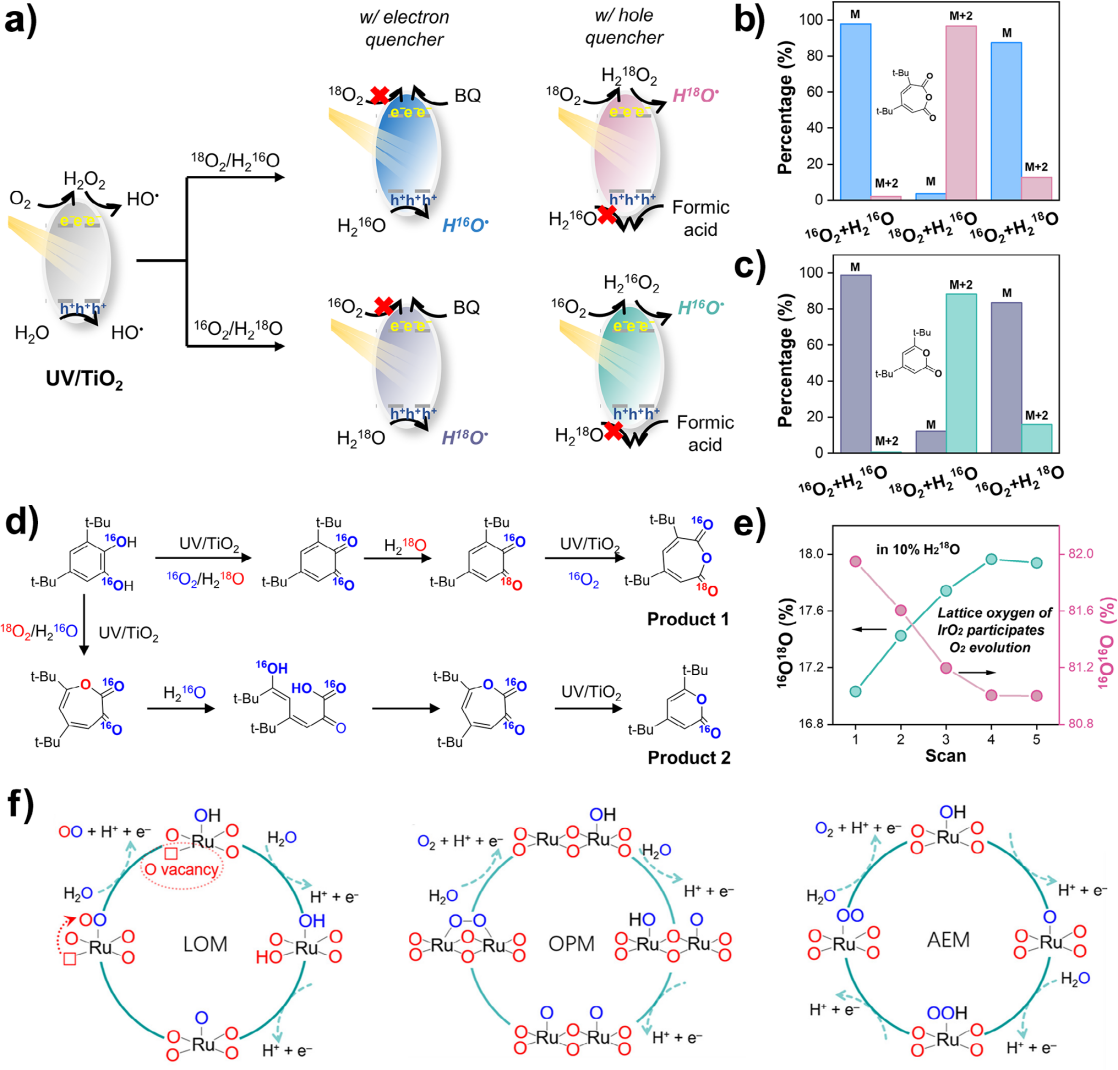
a) Illustration of the selective labeling of molecule O_2_ and H_2_O by ^18^O to investigate the role of photo‐generated electrons and holes in ^•^OH generation and simultaneously the corresponding quenchers are added to further confirm the mechanism. Oxygen‐isotope distribution of the two ring‐opening products of 3,5‐di‐tert‐butylcatechol: b) product 1 and c) product 2. Copyright 2014 American Chemical Society.^[^ ^205^ ^]^ d) Illustration of the possible oxygen exchange process. e) Electrochemical evolution of ^16^O^18^O and ^18^O^18^O in 10% H_2_^18^O solution by IrO_2_ electrode during five successive scans. Copyright 2007 Elsevier.^[^ ^206^ ^]^ f) Three possible OER mechanisms on RuO2 Copyright 2024 American Chemical Society.^[^ ^207^ ^]^

^18^O‐labeling techniques are also extensively studied in the photocatalytic degradation of pollutants.^[^ ^202^, ^203^, ^204^ ^]^ Given that photooxidation of organics includes both the photo‐generated electron (e^−^) induced ROS generation and photo‐generated hole (h^+^) induced oxidation, it is essential to label the e^−^ pathway and h^+^ pathway separately to figure out their respective contributions. In most cases, a set of control experiments under ^18^O_2_/H_2_^16^O and ^16^O_2_/H_2_^18^O conditions is sufficient to calculate the percentage of ^•^OH from O_2_ reduction or H_2_O oxidation. However, additional quenching experiments using benzoquinone e^−^ as quenchers and formic acid as h^+^ quenchers can further validate the results of isotope experiments (Figure 17a).^[^ ^203^ ^]^

Photocatalytic H_2_O_2_ production has attracted broad research interests in recent years owing to its high efficiency and sustainability. Both O_2_ reduction and H_2_O oxidation can contribute to H_2_O_2_ generation, highlighting the importance of distinguishing the source of H_2_O_2_. Generally, two methods are employed to identify the source of H_2_O_2_: (1) employ MnO_2_ to catalyze H_2_O_2_ decomposition into O_2_ and detect the *m/z* value of gaseous product through gas‐chromatograph/mass spectrometer ^[^ ^208^ ^]^; and (2) leverage the selective reaction between H_2_O_2_ and organic boranate or 4‐carboxyphenylboronic acid (with a second‐order reaction rate constant of approximately 2.2 M^−1^ s^−1^) and detecting the featured phenolic products through liquid‐chromatograph/mass spectrometer.^[^ ^209^, ^210^ ^]^

In terms of electrochemical OER, the origins of oxygen might come from either water or the lattice oxygen. For example, Fierro et al. found that the percentage of ^16^O^18^O from OER increased from 17% to the theoretical maximum 18% after five scans in 10% H_2_^18^O solution using Ir^16^O_2_ as the anode (Figure 17e).^[^ ^206^ ^]^ This phenomenon indicates that the generation of reactive intermediates in the OER process involves the participation of lattice oxygen, supporting the lattice‐oxygen‐mediated mechanism (LOM). However, if the evolution of O_2_ is only induced by the adsorbed H_2_O or OH^−^ (Figure 17f), following the adsorbate evolution mechanism (AEM), the isotopic content remains 18% from the initial scan. Similarly, if the OER catalyst proceeds via a O*─O* radical coupling reaction, i.e., oxide path mechanism (OPM), the isotopic distribution of O_2_ would be only influenced by the ^18^O percentage in the solvent. Liu et al.^[^ ^211^ ^]^ also found that the ozone generated from electrochemical water (H_2_^18^O) oxidation by Pb_3_O_4_ pre‐catalyst produces ^48^O_3_ dominantly, which confirms that the ozone evolution proceeds a LOM pathway.

### 4.2 Analysis of the Origins of Nitrogen

Isotope labeling is a reliable tool for eliminating interference from environmental nitrogen contamination and comprehending the mechanism of nitrate/nitrite reduction reactions.^[^ ^212^, ^213^ ^]^ After the labeling with ^15^N, a characteristic *m/z* shift of+1 or a different splitting pattern could be observed in MS and ^1^H NMR, respectively.^[^ ^212^ ^]^ If the generated NH_4_^+^ completely originates from NO_3_^−^, the produced NH_4_^+^ concentration with ^15^NO_3_^−^ as the substrate should be consistent with that using ^14^NO_3_^−^ as the substrate; both of ^15^NH_4_^+^ and ^14^NH_4_^+^ could be quantified using a standard curve method via NMR.^[^ ^214^ ^]^

**Figure 18:**
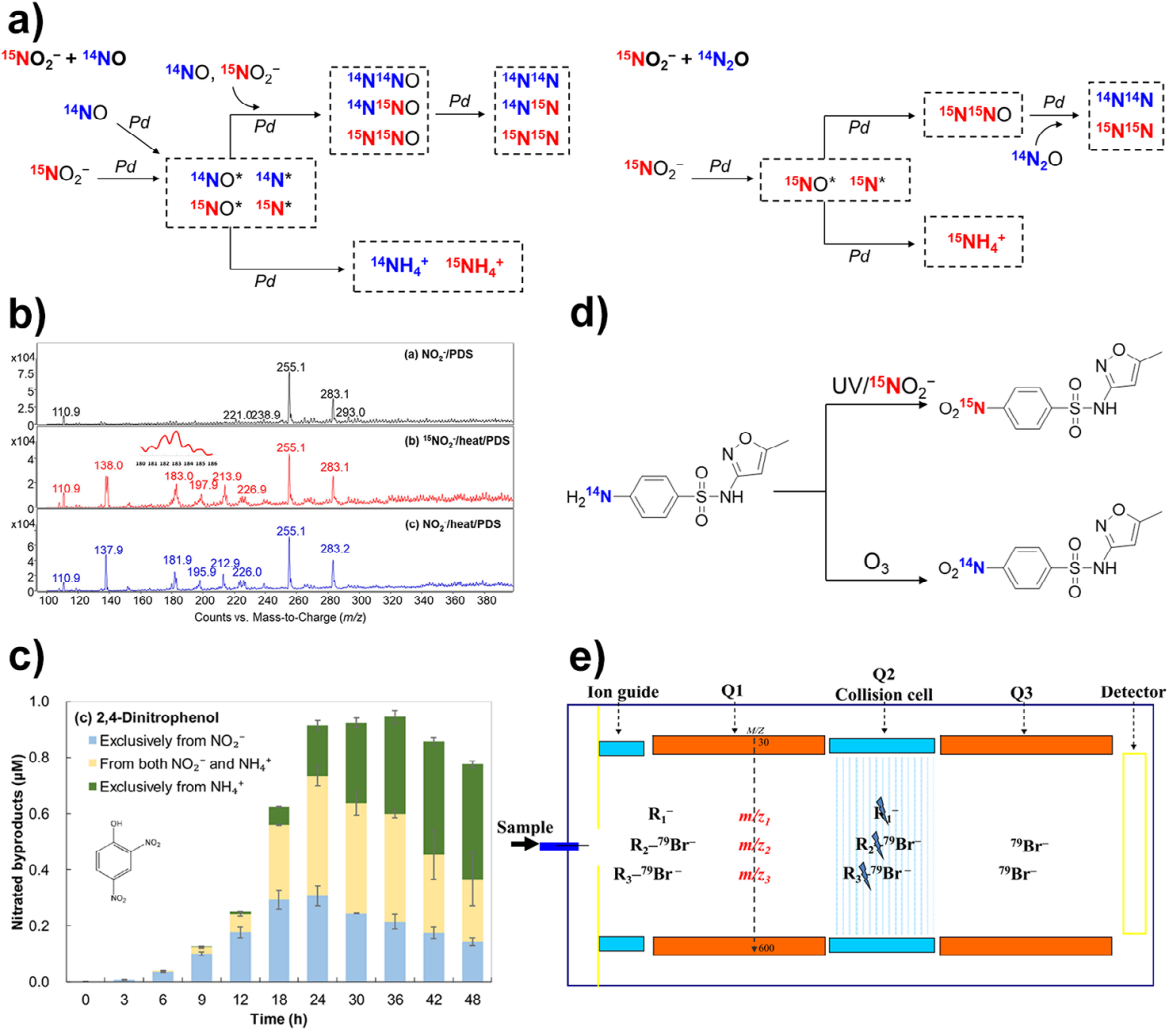
a) Illustration of the isotope experiments of ^15^NO_2_− reduction in the presence of ^14^NO or ^14^N_2_O by Pd‐based catalysts and hydrogen gas. b) MS of natural organic matters subjected to heat/PDS treatment in the presence of nitrite. Copyright 2019 American Chemical Society.^[^ ^216^ ^]^ c) Formation of p‐nitrophenol and 2,4‐dinitrophenol after heat/PDS oxidation of natural organic matters in the presence of both NO_2_− and NH_4_+. Copyright 2022 American Chemical Society.^[^ ^217^ ^]^ d) Illustration of the formation of nitro DBPs during UV/NO_2_− system and O_3_ oxidation. e) Illustration of the working mechanism of the Precursor Ion Scan (PIS) method. Copyright 2013 American Chemical Society.^[^ ^218^ ^]^

Moreover, ^15^N labeling is also conducive to the identification of the transformation pathway of N species during nitrate/nitrite reduction. For example, the simultaneous addition of ^15^NO_3_^−^, ^14^NO_2_^−^, and ^14^NH_4_^+^, followed by monitoring of the isotopic abundance of each species, demonstrate that neither reoxidation nor disproportionation occurs during the reduction of NO_3_^−^ by Fe^2+^, thereby ruling out the regeneration of NO_3_^−^.^[^ ^215^ ^]^ Zhang et al.^[^ ^20^ ^]^ designed a double‐substrate experiment using ^15^NO_2_^−^/^14^NO and ^15^NO_2_^−^/^14^N_2_O as the reactants to probe the NO_2_^−^ reduction pathway in the Pd‐In/H_2_ system (Figure 18a). They found that only ^15^NO_2_^−^/^14^NO could produce ^14^N^15^N (*m/z* 29), while ^15^NO_2_^−^/^14^N_2_O only produced ^14^N^14^N and ^15^N^15^N. These results confirmed that only NO participates in the N─N coupling reaction, whereas N_2_O is directly reduced into N_2_.

^15^N labeling is also effective in tracing the generation of reactive nitrogen species, such as NO_2_^•^, NO^•^, and ONOO^−^, as well as the formation of nitroso/nitro DBPs in UV/NO_2_^−^, UV/NO_3_^−^, UV/NH_2_Cl, UV/PDS/NO_2_^−^, and UV/PDS/NH_4_^+^ processes.^[^ ^43^, ^217^, ^219^, ^220^, ^221^ ^]^ As shown in Figure 18b, the ^15^N labeling techniques enable the fast screen and recognition of potential nitrogen‐containing products by the shift of *m/z* when the ^14^NO_2_^−^ in NO_2_^−^/heat/PDS system is replaced with ^15^NO_2_^−^.^[^ ^219^ ^]^ The +1 *m/z* shift indicates the incorporation of one nitrogen group, while the +2 shift indicates the incorporation of two nitrogen groups. In addition to MS, the ^15^N NMR spectrum of the reacted solution also shows an obvious chemical shift of 376.59 ppm, which precisely fits the theoretical N shifts in 4‐nitrophenol, 4‐hydroxyl‐3‐nitrobenzoic acid, and 2,4‐dinitrophenol determined by a DFT calculation.^[^ ^216^ ^]^ The individual contributions of NH_4_^+^ and NO_2_^−^ in the heat/PDS system could also be distinguished by the single labeling of NH_4_^+^ with ^15^N (Figure 18c).^[^ ^217^ ^]^ Finally, the ^15^N labeling could distinguish the origins of N from the contaminant itself or from NO_2_^−^. For example, as depicted in Figure 18d, the DBPs formed in the UV/NO_2_^−^ system are formed via the electrophilic substitution of ^•^NO_2_ on aromatic rings, rather than through the oxidation of the amine group.^[^ ^220^ ^]^

### 4.3 Analysis of the Origins of Carbon and Hydrogen

The carbon isotope is widely used in synthesis and hydrocarbon conversions such as CO_2_ reduction,^[^ ^222^ ^]^ while its application in contaminant degradation is relatively limited. One notable example is the use of 1,2,3,4─^13^C‐PFOA to identify the elimination pathway of CF_2_ moieties during the UV/I^−^ treatment of PFOA.^[^ ^200^ ^]^ Through MS analysis, only 1,2,3─^13^C‐perfluoroheptanoic acid and 1,2–^13^C‐perfluorocaproic acid were detected, while 1,2,3,4─^13^C ‐perfluoroheptanoic acid and 1,2,3,4─^13^C ‐perfluorocaproic acid were not detected. This suggests that the elimination of CF_2_ moieties initiates from the C atom near the carboxylic groups. Besides, Zhang et al. also synthesized ethylparabens containing ^13^C labels at different positions of the molecule to ascertain DBP formation mechanisms from the chlorination process of phenols.^[^ ^223^ ^]^

Most studies focus on tracing hydrogen by using nonexchangeable H. For example, Lin et al.^[^ ^224^ ^]^ analyzed the evolution of H_2/_HD/D_2_ during the degradation of microplastic (with aliphatic C─H bond) in D_2_O solution. The products dominantly consisted of H_2_, suggesting that the source of proton mainly originated from the solvent. However, Zhang et al. ^[^ ^225^ ^]^ studied the successive HER performance of PtC catalysts in D_2_O and H_2_O solutions. The emergence of D_2_ and HD signals in H_2_O indicated that PtC catalysts were poisoned by the HER intermediates.

### 4.4 Analysis of Chlorinated and Brominated Degradation Intermediates

Chlorine and bromine possessed high natural isotope abundances. The natural isotope ratios of ^35^Cl:^37^Cl (3:1) and ^79^B:^81^Br (1:1) endow any chlorinated compounds or brominated compounds with a nearly constant distribution of the *m/z* values. These isotope patterns should be carefully considered when analyzing the degradation pathways of chlorinated or brominated compounds. With the increase of the numbers and the types of halogen atoms, the distribution pattern would be more sophisticated, with some common examples provided in Figure S2.^[^ ^226^ ^]^

Leveraging this characteristic, Zhang et al. developed a fast and accurate method, the precursor ion scan (PIS) method, to detect any chlorinated or brominated polar compounds in drinking water.^[^ ^218^, ^226^, ^227^ ^]^ The working mechanism of PIS with the configuration of the electrospray ionization‐triple quadrupole mass spectrometer is illustrated in Figure 18e. In short, any compounds that could generate the fragment ion of 35/37 or 79/81 from the total ion chromatography with the same retention time could be reckoned as possible chlorinated or brominated products. This approach significantly enhances the signal‐to‐noise ratio, allowing more products to be screened out. For example, the degradation products of 3‐brominated phenol by Fe(IV) could be easily recognized as polymerized 3‐brominated phenol via the PIS method.^[^ ^228^ ^]^

### 4.5 Combination with Other Characterizations and DFT Calculation

#### 4.5.1 Infrared Spectroscopy

**Figure 19:**
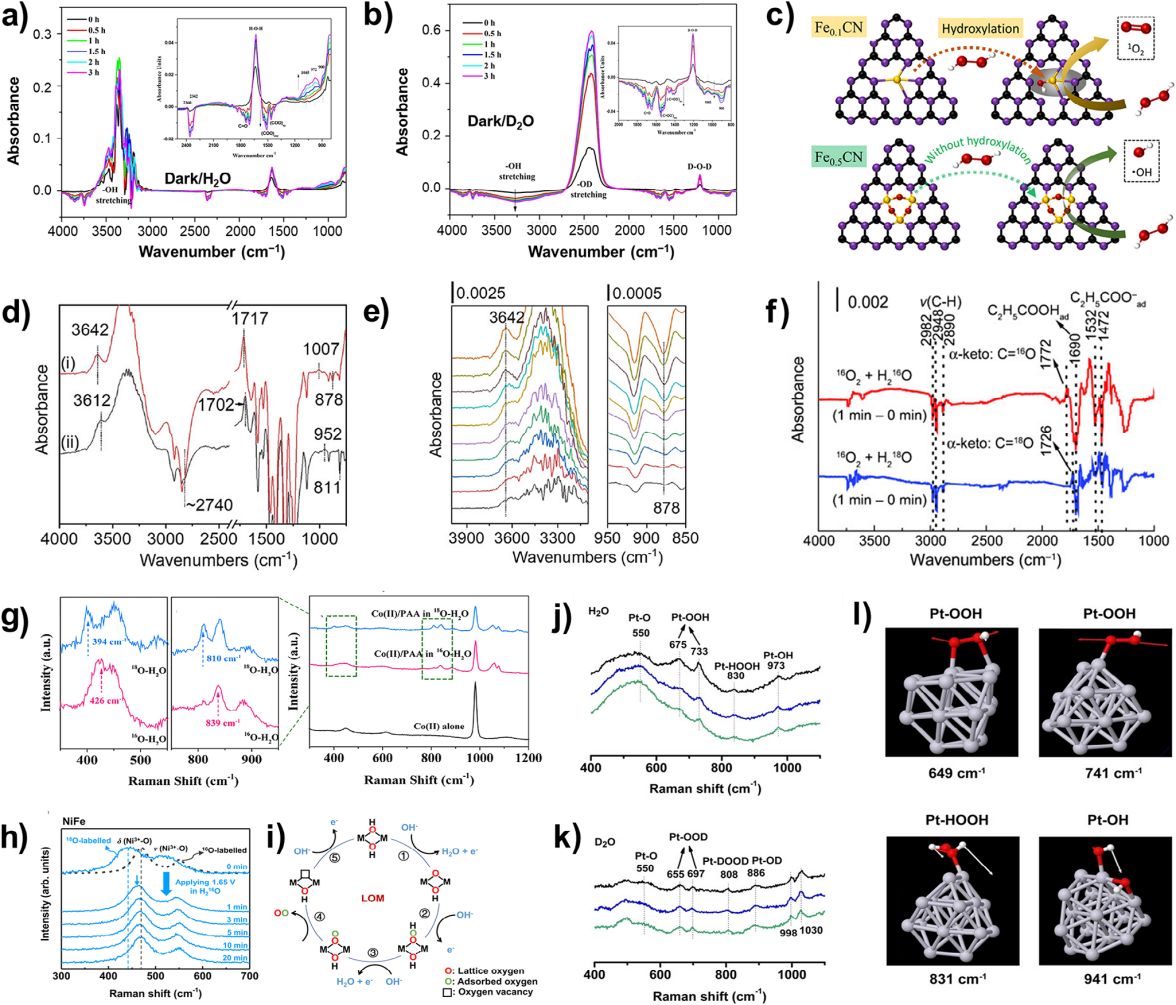
Time evolution of the attenuated total reflection Fourier transform infrared (ATR‐FTIR) spectra of adsorbed acetate on TiO_2_ a) with H_2_O at pH 6.0 and b) in D_2_O at pD 6.4. Copyright 2016 Elsevier.^[^ ^229^ ^]^ c) Illustration of the different H_2_O_2_ activation pathway by single‐atom Fe_0.1_CN catalyst. d) IR spectra collected after illumination on Fe_0.1_CN with (i) H_2_^16^O_2_ and (ii) H_2_^16^O_2_. e) Series of IR spectra collected after different illumination durations on Fe_0.1_CN/H_2_^16^O_2_. Copyright 2022 American Chemical Society.^[^ ^234^ ^]^ f) Difference diffuse reflectance infrared Fourier transform spectroscopy (DRIFTS) spectra obtained for the decarboxylation of propionic acid by TiO_2_ photocatalysis after 1 min of UV irradiation using H_2_^16^O (upper line) and H_2_^18^O (lower line). Copyright 2010 Wiley‐VCH.^[^ ^235^ ^]^ g) In situ Raman spectra views of Co(II), PAA, and Co(II)/PAA in H_2_^16^O or H_2_^18^O matrix. Copyright 2021 Elsevier.^[^ ^17^ ^]^ h) Quasi in situ Raman spectra of ^18^O‐labeled NiFe (oxy)hydroxides after being applied a positive potential of 1.65 V with H_2_^16^O for different times. i) Schematic illustration of the LOM pathway on NiFe (oxy)hydroxide. Copyright 2022 Springer Nature.^[^ ^85^ ^]^ Raman spectra of Au@Pt nanoparticles to detect the generation of ROS in j) H_2_O and k) D_2_O. l) Optimized adsorption configurations (side views) and vibrational frequencies of different ROS on Pt clusters. Copyright 2021 Wiley‐VCH.^[^ ^236^ ^]^

Infrared spectroscopy (IR) is one of the most utilized techniques to analyze the presence of reaction intermediates or change of functional groups on the catalyst surface. However, the strong IR vibration absorption of H_2_O originating from the solvent and the atmosphere can interfere with the measurement. To address this, D_2_O is usually utilized in lieu of H_2_O, as it eliminates the interference in the critical wavenumber regions.^[^ ^84^, ^229^, ^230^, ^231^, ^232^ ^]^ For instance, the ─OH stretching band (3000–3600 cm^−1^) obviously shifts toward lower wavenumber regions (2300–2700 cm^−1^) after the solvent‐exchange from H_2_O to D_2_O. In addition, the bands of molecular bending modes of H_2_O, δ (H─O─H) would shift from 1638 to 1205 cm^−1^.^[^ ^229^ ^]^ As a result, these shifts enable clear observation of the stretching vibrations of acetic acids (1400–1800 cm^−1^), without interference from the molecular bending bands of H_2_O (Figure 19a,b).^[^ ^229^ ^]^ Besides, D_2_O could circumvent the limitation of H_2_O in IR measurement during the wavenumber range of 600–850 cm^−1^.^[^ ^233^ ^]^ The enhanced transmittance in D_2_O would facilitate the detection of many important species like metal‐oxo bonds or peroxyl bonds, which all featured low wavenumbers.

Another application of isotope in IR measurement is to identify the reaction intermediates based on the isotope shifts. An approximate estimation of the isotope shift can be calculated based on Hooke's laws, whereas DFT calculations can provide accurate simulations and predictions of the band position of the intermediates. The increased molecule mass of isotopes results in decreased wavenumber values of specific intermediates, analogous to the shifts observed with D_2_O. Sheng et al.^[^ ^234^ ^]^ used H_2_^18^O_2_ to investigate the activation of H_2_O_2_ by a single‐atom Fe_0.1_CN catalyst (Figure 19c,e).^[^ ^234^ ^]^ The 1007 cm^−1^ band could be assigned to the adsorbed O_2_^•−^, with a significant ^18^O/^16^O isotope shift to 952 cm^−1^, confirming that O_2_^•−^ originates from the oxidation of H_2_O_2_ rather than the reduction of molecular O_2_. Besides, the relatively smaller isotope changes at 3642 cm^−1^ (to 3612 cm^−1^) and 1717 cm^−1^ (to 1702 cm^−1^) indicate only one ^18^O atom is included in these functional groups, likely corresponding to the O─H stretching and Fe─O─H bending vibrations of the surface hydroxyl formed in situ from H_2_^18^O_2_ activation.

When reaction signals are too weak for direct observation, differenced spectra can be used to analyze the presence of intermediates. As shown in Figure 19f, the isotopic bands at 1772 and 1726 cm^−1^, corresponding to the C─O stretching of the α‐keto group of pyruvic acid, confirm that the oxidation of propionic acid in UV/TiO_2_ system proceeds via the hydroxylation of α‐carbon and the subsequent oxidation to carbonyl moieties. Meanwhile, the absence of alcohol or aldehyde intermediates excludes the contribution of the conventional Russell mechanism.

#### 4.5.2 Raman

In situ Raman spectrometer is another powerful tool for the identification and analysis of the reaction intermediates.^[^ ^237^, ^238^ ^]^ Similar to IR, the ^18^O/^16^O shift can also confirm the presence of HMOS like Co(IV),^[^ ^17^ ^]^ Fe(IV),^[^ ^32^ ^]^ and Mn(IV),^[^ ^239^ ^]^ as well as other surface metal‐oxo or peroxyl species.^[^ ^240^, ^241^ ^]^ As shown in Figure 19g, the isotopic pair band of 394/426 cm^−1^ can be reasonably assigned to the diatomic Co─O oscillator, a signature of terminal Co─O bond, as observed isotopic shift 32 cm^−1^ closely matches the calculated value of 34 cm^−1^. In addition, the vibrational frequency at 810/839 cm^−1^ can be ascribed to the O─O harmonic oscillator, which stems from the side‐on Co(II)−PAA complex.

The ^18^O‐labeling can also be applied to distinguish the AEM and LOM mechanisms in the OER process.^[^ ^85^, ^242^ ^]^ In the case of the LOM mechanism, the ^18^O‐labeled δ (Ni^3+^−O), ν (Ni^3+^−O), and ν (O─O) bands shift to higher wavenumbers upon being electrolyzed in H_2_^16^O solution, because the lattice oxygen is substituted by solvent oxygen (Figure 19h,i). In contrast, no isotope is observed in the AEM mechanism. Intriguingly, for the high‐valent solid (oxy)hydroxide catalysts, the oxygen exchange between the lattice oxygen and the solvent only occurs at high anode potentials. In contrast, for of Ni(II)−(oxy)hydroxide, the oxygen exchange quickly happens even at open circuit potential.^[^ ^242^ ^]^

In the case of other ROS, the Raman intensity might not be sufficient for clear observation. However, the presence of noble metal particles can promote the adsorption of the intermediate ROS on the metal surfaces, thus enabling the detection of ROS through Raman spectra. Furthermore, when the solution is substituted by D_2_O, the corresponding bands of various intermediates would redshift if the intermediates contain hydrogen atoms, providing insights into the protonation states of intermediates (Figure 19j,k). Supplementary DFT calculations of the Raman signals can further consolidate the judgment of the band locations and nature of different ROS (Figure 19l).

#### 4.5.3 EPR and NMR

**Figure 20:**
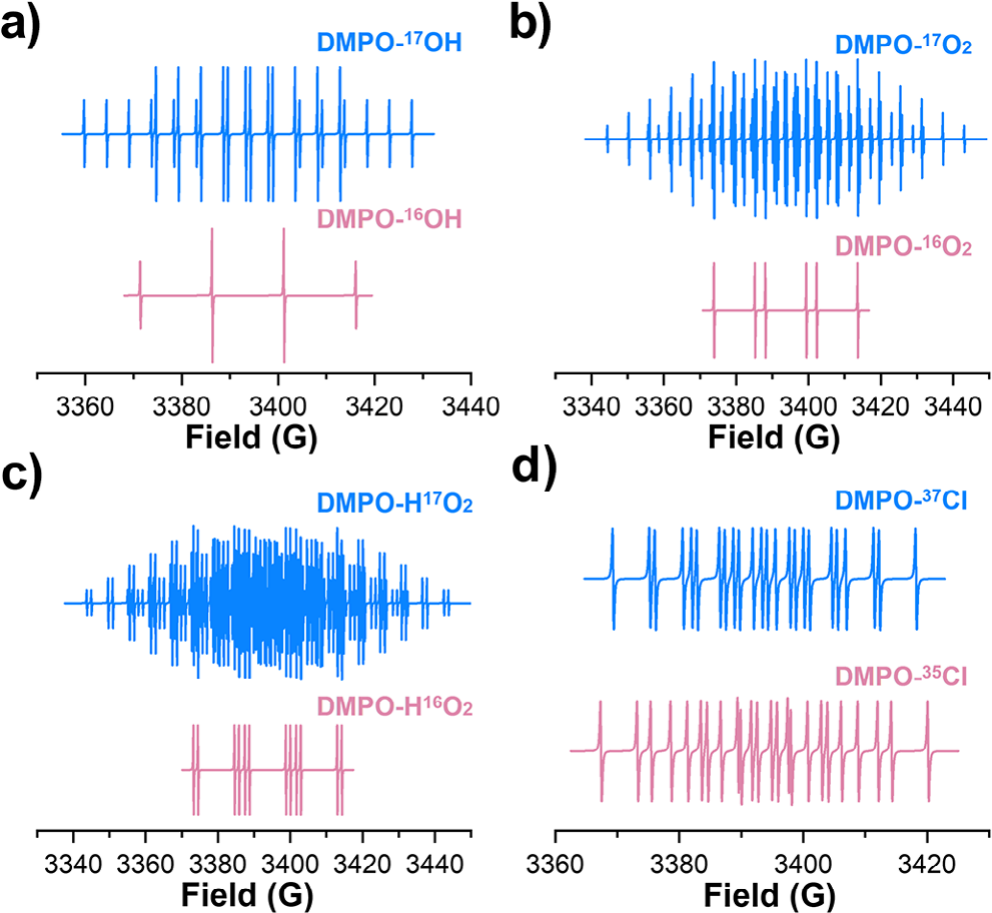
a) Simulated EPR spectrum of DMPO‐^16^OH (αN = 1.49 mT, αβH = 1.49 mT) and DMPO‐^17^OH (αN = 1.49 mT, αβH = 1.49 mT, αβO(^17^O) = 0.46 mT). b) Simulated EPR spectrum of DMPO‐^16^O_2_ (αN = 1.42 mT, αβH = 1.134 mT) and DMPO‐^17^O_2_ (αN = 1.42 mT, αβH = 1.134 mT, αβO(^17^O) = 0.59 mT). c) Simulated EPR spectrum of DMPO‐^16^HO_2_ (αN = 1.42 mT, αβH = 1.134 mT, αγH = 0.15 mT) and DMPO‐^17^HO_2_ (αN = 1.42 mT, αβH = 1.134 mT, αγH = 0.15 mT, αβO(^17^O) = 0.59 mT). d) Simulated EPR spectrum of DMPO‐^35^Cl (αN = 1.134 mT, αH = 0.589 mT, αCl = 0.814 mT) and DMPO‐^37^Cl (αN = 1.134 mT, αH = 0.589 mT, αCl = 0.678 mT).

As previously discussed, the appearance of the DMPO‐OH signal in the EPR spectrum is not conclusive evidence of the generation of ^•^OH, because other ROS can produce false‐positive signals of DMPO‐OH. However, such limitation can be well addressed by combining with ^17^O‐labeling techniques, which yield specific spectrum of ^17^O‐labeled ^•^OH (Figure 20a). The relative contribution of different pathways to the generation of DMPO‐OH could also be quantified via the simulation of experimental results. Wang et al.^[^ ^38^ ^]^ used H_2_^17^O to investigate the origins of DMPO‐OH in the Cu‐based peroxide activation process. They found that the detected DMPO‐OH adduct originates completely from water in the Cu(II) alone system. However, in the presence of peroxides, over 99.8% of the detected ‐OH is derived from the oxidant. In addition, the ^17^O‐labeled HO_2_^•^, O_2_^•−^, and ^35^Cl/^37^Cl‐labeled Cl^•^ radicals also exhibit different spectra in EPR analysis. These differences could be utilized to distinguish the origins of HO_2_^•^ and O_2_^•−^, whether from molecular O_2_ reduction or peroxide oxidation, as well as to analyze the spectral composition of DMPO‐Cl (Figure 20b–d).^[^ ^243^ ^]^

^17^O solid‐state NMR is another robust characterization tool, that can probe the chemical environment around the oxygen anions in solid materials.^[^ ^37^ ^]^ Compared with other techniques, such as electron microscopy, ^17^O NMR results represent the whole sample rather than a limited field of view, thus providing detailed structural and dynamic information of surface chemistry and reactions on a catalyst. For example, three TiO_2_ samples with different exposed facets exhibit distinctive chemical shifts ranging from ‐200 to 1200 ppm, which could be used to analyze the percentage of different facets in an unknown sample.^[^ ^244^ ^]^ Furthermore, a comparison between the experimental spectrum and the simulation spectrums obtained from DFT calculations can provide further insights into how water molecules are adsorbed and dissociated on different TiO_2_ surfaces.

## 5 Summary and Outlook

As a fingerprint technology for studying the reaction mechanism and tracing element transformations, isotope techniques have become increasingly pivotal in aqueous reactions, particularly environmental chemistry. Its dual functions as kinetic probes and atomic tracers offer a great opportunity for most chemists to get insights into remediation reaction systems, especially when the conventional experiment methods fail to evidence reaction mechanisms. However, isotope techniques also have limitations in some scenarios. The ubiquitous isotope exchange and the multi‐possibility of the KIE experiments may complicate the explanation of the experiment results. Therefore, careful experimental design and cautious interpretation of the data are essential. Herein, we summarize several key considerations and future outlooks for isotope techniques:

1. **Conducting gradient experiments with varying concentrations of isotope**. For the calculation of KSIE and the quantitative calculation of element origins, using single isotope concentration may not reflect the overall effects of isotope substitution. Conducting gradient experiments with varying isotope concentrations provides more comprehensive information and excludes exceptional cases, as discussed in the theoretical KSIE. Meanwhile, the isotope concentration should also be carefully noted in the experimental section.
2. **Isotope exchange with solvent affects both reactants and products**. While rough estimations based on exchange rates are helpful, real‐time monitoring using MS or NMR can provide more precise information on the duration required for the complete exchange.
3. **Careful selection of KIE calculation parameters**. Although the degradation rate of contaminants is commonly used to calculate the KIE, the system current density and product (or intermediate) generation rate may also be relevant for the calculation. Different parameters can emphasize different elementary steps, leading to variations in KIE values.
4. **Symmetric labeling of reactants**. All possible element sources should be considered in the tracing experiment. For example, H_2_O, O_2_, and the oxidants could all serve as the donors of oxygen, while NO_2_^−^, NH_4_^+^, and the amine group in the contaminant can serve as the donors of nitrogen. Separate labeling of each component enhances the reliability of the tracing experiments and mitigates interferences from isotope exchange.
5. **Addressing limitations in isotope‐enriched reagents**. Due to the unavailability of isotope‐enriched chemicals and the rapid solvent exchange reactions, the investigations in many reactions are not suitable through isotope experiments, such as the generation and consumption of HOCl. Besides, for some oxidants like PMS, the labeling ratio is relatively low. Thus, developing a cost‐effective and sensitive method for the fast and efficient labeling of such substances is a critical direction to explore.
6. **Expanding KIE studies to underexplored reactive species**. To the best of our knowledge, limited research has been conducted to investigate KIEs of less reactive species, such as CO_3_^•−^, Cl_2_^•−^, ClO^•^, ClO_2_, Br^•^, Br_2_^•−^, HONOO, and NO_2_. These should be explored further.
7. **Integrating isotope experiments with complementary methods**. The results of isotope experiments should not be overinterpreted. Kinetic studies, quenching experiments, and spectroscopic characterizations are equally important. Therefore, a comprehensive understanding of all the available results will be conducive to the accurate interpretation of the phenomenon.
8. **Adopting novel experimental methods and machine learning in the investigations of chemical wastewater treatment**. For example, advanced techniques such as steady‐state isotopic transient kinetic analysis (SSITKA) have proven effective in identifying the reactive intermediate in the synthesis of NH_3_^[^ ^213^ ^]^ and hydrogenation of CO_2_.^[^ ^245^ ^]^ Thus, their applications in chemical wastewater treatment can provide new insights. Additionally, substantial KIE and KSIE data of diverse ROS can serve as the training set to feed machine learning, enabling a better prediction of KIE and KSIE in unknown reactions.

## Conflict of Interests

The authors declare no conflict of interest.

## Supporting information



Supporting information

## Data Availability

The data that support the findings of this study are available from the corresponding author upon reasonable request.
