# Supplementary material for: Isotope Techniques in Chemical Wastewater Treatment: Opportunities and Uncertainties
Source: Angew Chem Int Ed Engl. 2025 Mar 18;64(19):e202422892. doi: 10.1002/anie.202422892 (PMC12051784; doi:10.1002/anie.202422892)
Supplement: Supplementary file 1 — Supporting information [file ANIE-64-e202422892-s001.docx]

Supporting Information
©Wiley-VCH 2024
69451 Weinheim, Germany

Isotope Techniques in Chemical Wastewater Treatment: Opportunities and Uncertainties

Hongyu Zhou^[a][b]^, Xiaoguang Duan*^[b]^, Bingkun Huang^[a]^, Shuang Zhong^[b]^, Cheng Cheng^[b]^, Virender K Sharma^[c]^, Shaobin Wang^[b]^, Bo Lai*^[a]^

[a] Hongyu Zhou, Bingkun Huang, and Prof. Bo Lai
State Key Laboratory of Hydraulics and Mountain River Engineering, College of Architecture and Environment
Sichuan University
Chengdu 610065, China
E-mail: [laibo@scu.edu.cn](mailto:laibo@scu.edu.cn)

[b] Hongyu Zhou, Dr. Shuang Zhong, Dr. Cheng Cheng, Prof. Xiaoguang, Duan, Prof. Shaobin Wang
School of Chemical Engineering
The University of Adelaide
Adelaide, SA 5005, Australia

E-mail: [xiaoguang.duan@adelaide.edu.au](mailto:xiaoguang.duan@adelaide.edu.au)

[c] Prof. Virender K Sharma
Department of Chemical, Environmental, and Materials
University of Miami
1251 Memorial Drive, Coral Gables, Florida 33146, United States

**This file of supporting information contains 4 Texts, 3 Figures and 10 Tables.**


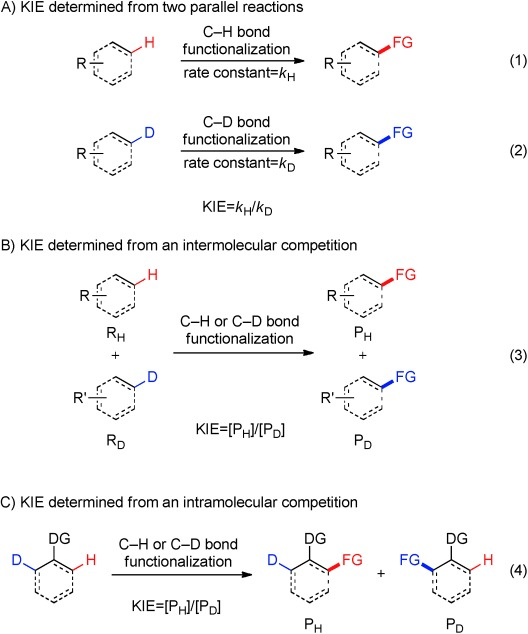


**Figure** **S1.** Different ^1^H/^2^H KIE calculation methods in three KIE experiments A–C. Copyright 2012 Wiley-VCH.^[1]^

**Text S1 Cage effects**

Theoretically, the cleavage of C−H bond would result in a primary KIE. However, for non-polar compounds, such as alkane, a KIE of unity is usually observed during the oxidation by ^•^OH in aqueous solution.^[2]^ For example, Pignatello et al.^[2b]^ found that the degradation of cyclohexane by UV/H_2_O_2_, UV/Fe^3+^ and Fe^2+^/H_2_O_2_ systems in aqueous solution exhibits an intermolecular KIE of 1.08, 1.12 and 1.06, respectively, whereas the degradation by gas-phase ^•^OH produces a KIE of 2.59.^[3]^ As for Fe(IV), the phenomenon is in accord with that of ^•^OH, i.e., a KIE of 1.26 and 10 is observed in aqueous system^[2b]^ and organic solvent system,^[4]^ respectively.

The origin of such contradictory results is the presence of cage effects.^[5]^ In the cage model, ^•^OH and the hydrophobic hydrocarbon molecules are entrapped in a solvent (H_2_O) cage. The contact between ^•^OH and the hydrophobic substrate is remarkably enhanced, and ^•^OH could not oxidize the substrate outside the cage until most of the substrate inside the cage is consumed.^[5]^ As a consequence, the hydrogenated substrate and deuterated substrate would be non-selectively attacked by ^•^OH, resulting in a lower KIE value. Therefore, it is recommended not to use non-polar substrate in mechanism investigation due to the tiny magnitude of KIE. However, it is noteworthy that the intramolecular KIE would not be influenced by the cage effects, because the C−H and C−D bonds are present in the same molecule.

**Text S2 Theoretical calculations of the ^1^O_2_-induced KSIE**

Assuming the formation rate of ^1^O_2_ and the reaction rates with ^1^O_2_ would not be altered by the displacement of the solvent, then we could obtain eqs. 1-4

$\text{k}_{\text{H}_{\text{2}}\text{O}}\text{ = }\text{k}_{\text{contaminant, }{}_{\text{ }}^{\text{1}}{\text{O}_{\text{2}}}}\text{ × [}{}_{\text{ }}^{\text{1}}{\text{O}_{\text{2}}\text{]}_{\text{ss, }\text{H}_{\text{2}}\text{O}}\text{ }}\text{× [C]}$ (eq. 1)

$\text{k}_{\text{D}_{\text{2}}\text{O}}\text{ = }\text{k}_{\text{contaminant, }{}_{\text{ }}^{\text{1}}{\text{O}_{\text{2}}}}\text{ × [}{}_{\text{ }}^{\text{1}}{\text{O}_{\text{2}}\text{]}_{\text{ss, }\text{D}_{\text{2}}\text{O}}\text{ }}\text{× [C]}$ (eq. 2)

$\text{R}_{{}_{\text{ }}^{\text{1}}{\text{O}_{\text{2}}}}\text{ = [}{}_{\text{ }}^{\text{1}}{\text{O}_{\text{2}}\text{]}_{\text{ss}}\text{ × }}\text{[}\text{k}_{\text{d}}\text{ + }\text{k}_{\text{contaminant, }{}_{\text{ }}^{\text{1}}{\text{O}_{\text{2}}}}\text{ × [C]]}$ (eq. 3)

$\text{KSIE }\text{= }\frac{\text{k}_{\text{D}_{\text{2}}\text{O}}}{\text{k}_{\text{H}_{\text{2}}\text{O}}}\text{ = }\frac{\text{k}_{\text{d, }\text{H}_{\text{2}}\text{O}}\text{ + }\text{k}_{\text{contaminant, }{}_{\text{ }}^{\text{1}}{\text{O}_{\text{2}}}}\text{ × [C]}}{\text{k}_{\text{d, }\text{D}_{\text{2}}\text{O}}\text{ + }\text{k}_{\text{contaminant, }{}_{\text{ }}^{\text{1}}{\text{O}_{\text{2}}}}\text{ × [C]}}$ (eq. 4)

where $\text{k}_{\text{contaminant, }{}_{\text{ }}^{\text{1}}{\text{O}_{\text{2}}}}$ is the second-order reaction rates between ^1^O_2_ and the contaminant, $[{}_{\text{ }}^{\text{1}}{\text{O}_{\text{2}}\text{]}_{\text{ss}}}$ is the steady-state concentration of ^1^O_2_ in the system, $[C]$ is the concentration of the organic contaminant and $\text{R}_{{}_{\text{ }}^{\text{1}}{\text{O}_{\text{2}}}}$ is the formation rate of ^1^O_2_ in the system.

**Text S3** Other factors influenced by D_2_O

**(1) Redox potential and secondary reaction rate constants**

The change of redox potential induced by the substitution of isotope is the reflection of the change of driving force for the reaction. Generally, the lighter the mass, the faster the reaction rate, but for different oxidants, the extent of the influence caused by deuteration is different. In terms of ^•^OH and ^•^OD, there is only a small change of the reactivity and their second-order reaction rate constants with various substrate are all close to the diffusion-controlled reaction rate constants. For example, the KIE for the reaction of ^•^OH/acetone and ^•^OD/acetone is only 1.08 at 298 K.^[6]^ As for the transitional metal species like Fe^3+^, the change of redox potential is also determined by the ligand of Fe^3+^. The redox potential of hydration [Fe(H_2_O)]^3+^ is 0.04 V less than [Fe(D_2_O)]^3+^,^[7]^ while for [Fe(CN)_6_)]^3−^ the redox potential is hardly changed in D_2_O. Similarly, the anodic oxidation intermediate, NiOOH/Ni(OH)_2_, also exhibits a higher cyclic voltammetry potential in D_2_O.^[8]^ These increased redox potential of metal ions could be all attributed to the tighter solvent shell in D_2_O. Therefore, a greater increase in entropy is observed when the deuterated solvent structure relaxes during the reduction of Ni(III) to Ni(II). Due to the more favorable change in entropy that occurs upon the reduction of Ni(III), the redox peak would shift positively. Finally, for organic molecules, the change of redox potential is highly associated with the deuteration of the functional group. Figure S1 shows the change of redox potential of the benzoquinone/hydroquinone redox couple in D_2_O. Due to the in-situ deuteration of phenolic hydroxyl group, the cleavage of O−D bond is more difficult than that of O−H bond, thus resulting in the upshift of redox potential.


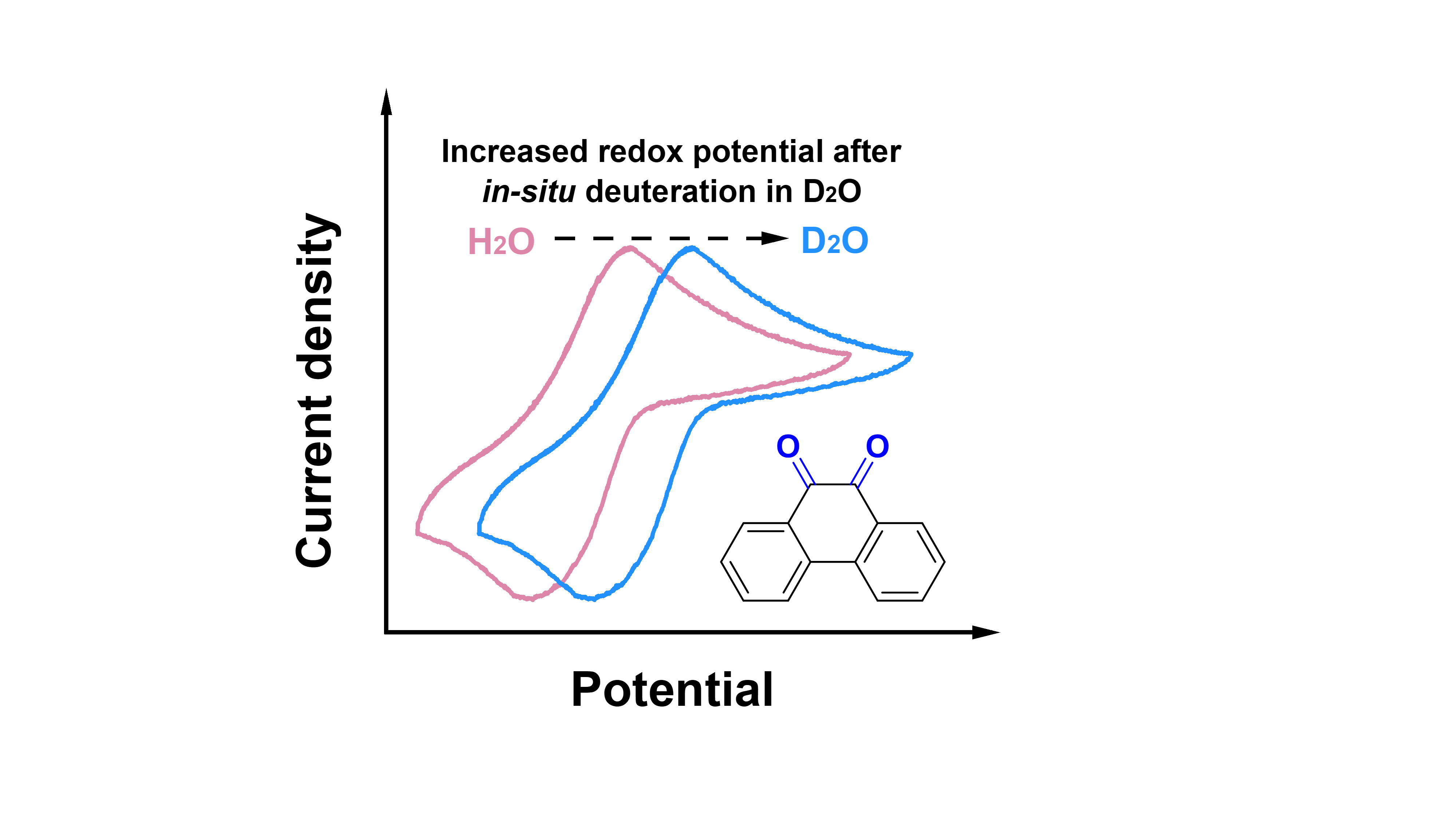


**Figure S2.** Change of the redox potential of benzoquinone moiety in D_2_O reflected by the cyclic voltammetry curves.

**(2) Hydrogen bond**

Due to the stronger interaction between O and D than that between O and H, the deuterium bond network is stronger than the hydrogen bond network. This difference would result in a tighter solvent shell for highly charged ions as mentioned above.^[9]^ Moreover, the diffusion coefficient and molecular motions of D_2_O/OD^−^ in the vicinity of metal oxide surface is slower than those of H_2_O/OH^−^ due to the strong hydrogen-bonding interaction with surface species.^[9a]^ This difference is especially crucial to the OER process at a high overpotential region because the diffusion of reactants is the rate-limiting step in this reaction.

**Text S4 Calibration of the isotope abundance in products**

Generally, in ^16^O_2_/H_2_^18^O system the calibration is conducted according to eqs. 5 and 6, while in ^18^O_2_/H_2_^16^O system the calibration is conducted according to eqs. 7 and 8.^[10]^

$\text{H}_{\text{2}}\text{O \% = }\frac{\text{C}_{\text{p}}\text{ - }\text{C}_{\text{n}}}{\text{C}_{\text{w}}\text{ - }\text{C}_{\text{n}}}\text{ × 100\%}$ (eq. 5)

$\text{O}_{\text{2}}\text{ \% = }\frac{\text{C}_{\text{w}}\text{ - }\text{C}_{\text{p}}}{\text{C}_{\text{w}}\text{ - }\text{C}_{\text{n}}}\text{ × 100\%}$ (eq. 6)

$\text{H}_{\text{2}}\text{O \% = }\frac{\text{C}_{\text{O}}\text{ - }\text{C}_{\text{p}}}{\text{C}_{\text{O}}\text{ - }\text{C}_{\text{n}}}\text{ × 100\%}$ (eq. 7)

$\text{O}_{\text{2}}\text{ \% = }\frac{\text{C}_{\text{p}}\text{ - }\text{C}_{\text{n}}}{\text{C}_{\text{O}}\text{ - }\text{C}_{\text{n}}}\text{ × 100\%}$ (eq. 8)

where *C*_p_, *C*_n_, *C*_w_ and *C*_O_ are the ^18^O percentages of the measured isotope abundance of the product, natural isotope abundance of the product, measured isotope abundance of solvent and isotope abundance of ^18^O-enriched O_2_.

**
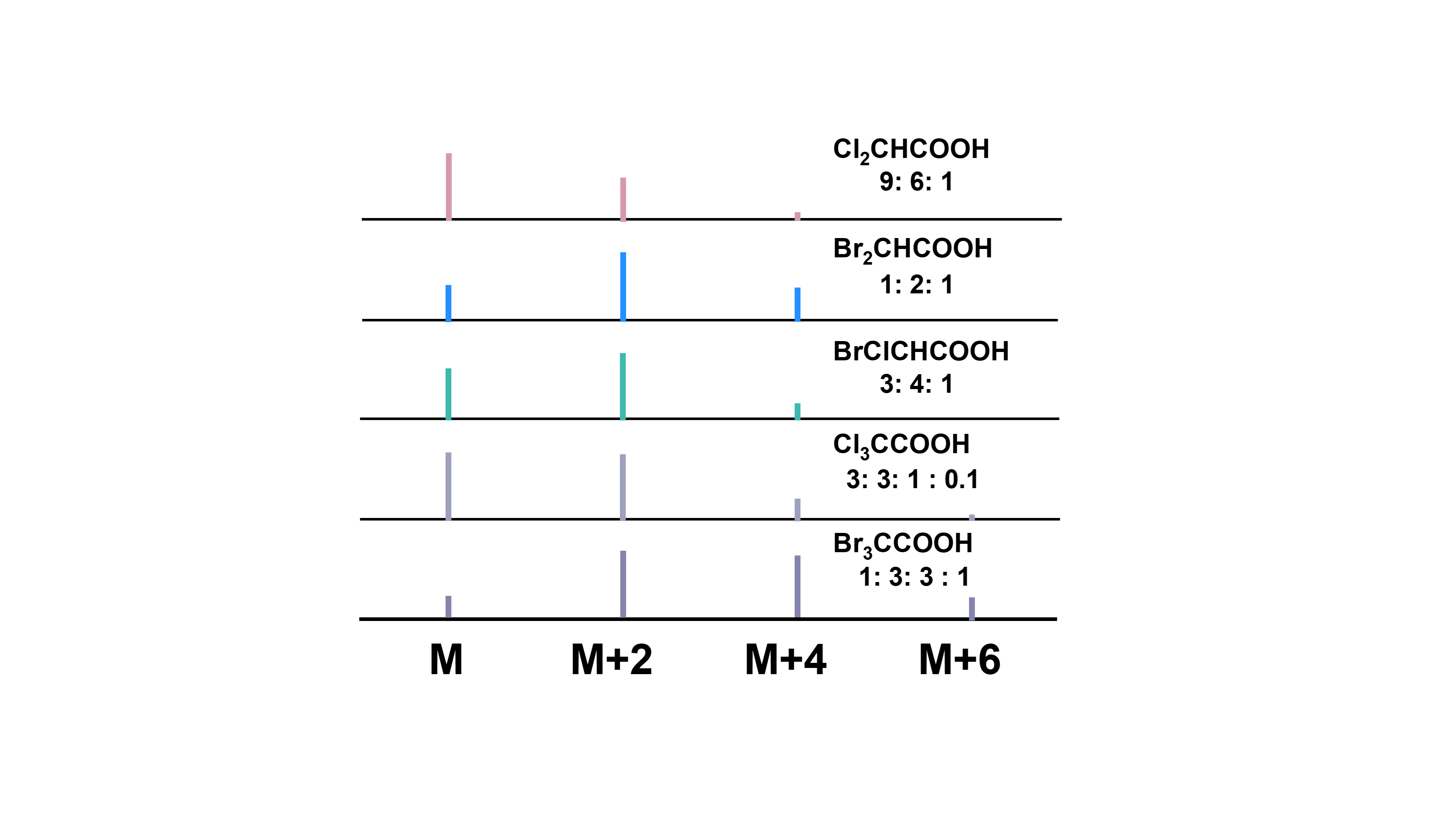
**

**Figure S3.** Isotopic distribution of *m/z* of five typical chlorinated and brominated acetic acids.

**Table S1.** The spins and natural abundance of the concerned isotopes.

| Elements | Isotopes | Isotopes, spin (abundance) |
| --- | --- | --- |
| H | ^1^H, ^2^D | 1/2 (99.985%), 1 (0.015%) |
| C | ^12^C, ^13^C, ^14^C | 0 (98.93%), 1/2 (1.07%), 0 (10^−12^) |
| N | ^14^N, ^15^N | 1 (99.632%), 1/2 (0.368%) |
| O | ^16^O, ^17^O, ^18^O | 0 (99.762%), 5/2 (0.038%), 0 (0.2%) |
| Cl | ^35^Cl, ^37^Cl | 3/2 (75.78%), 3/2 (24.22%) |
| Br | ^79^Br, ^81^Br | 3/2 (50.69%), 3/2 (49.31%) |

**Table** **S2.** The advantages and disadvantages of different ^•^OH and HMOS probes.

| Probes | Advantages as the probes of ^•^OH | Disadvantages as the probes of ^•^OH | Advantages as the probes of HMOS | Disadvantages as the probes of HMOS |
| --- | --- | --- | --- | --- |
| BA | Easily accessible | Interference from SO_4_^•−^ and Co(IV) since SO_4_^•−^ and Co(IV) could also transform BA to HBA | / | / |
| Terephthalic acid (TA) | Similar to BA but without HTA isomers | Low solubility at low acidic conditions | / | / |
| PMSO | Only ^•^OH could transform PMSO to PMSO−OH, while SO_4_^•−^ and other ROS produce other products; capable of distinguishing the secondary ^•^OH derived from other ROS | The concentration of secondary ^•^OH might be low since PMSO could react with other ROS via different mechanism and thus the concentration of secondary ^•^OH would be low | Easy to be measured via HPLC and HPLC-MS; easier adsorption onto catalyst surface due to the lower polarity | phenolic oxidation products are generated which could lead to unknown formation pathway of sulfone |
| PMSO_2_ | / | Interference from SO_4_^•−^ since SO_4_^•−^ could directly transform PMSO_2_ to PMSO_2_-OH | / | / |
| DMSO | / | / | Higher polarity and no phenolic oxidation products | Inconvenient to measure |

**Table S3.** Oxygen exchange reaction rates between various inorganic oxyanions and water.

| Oxyanions | Reactions | Rate constants | Half-life period t_1/2_ | Reference |
| --- | --- | --- | --- | --- |
| H_2_AsO_4_^−^ | H_2_O + H_2_AsO_4_^−^ → | 1 × 10^−4^ s^−1^ | 116 min | ^[11]^ |
| HSeO_3_^−^ | H_2_O + HSeO_3_^−^ → | 2 × 10^−4^ s^−1^ | 57 min | ^[11]^ |
| IO_3_^−^ (pH = 7.5) | H_2_O + IO_3_^−^ ↔ H_2_IO_4_^−^ (Both acid and base catalyzed) | 3.2 × 10^−4^ s^−1^ | 36 min | ^[12]^ |
| HCrO_4_^−^ (pH = 7) | H_2_O + HCrO_4_^−^ → ;  HCrO_4_^−^ + HCrO_4_^−^ → Cr_2_O_7_^−^ + H_2_O | 2.4 × 10^−3^ s^−1^ | 4.81 min | ^[11]^ |
| FeO_4_^2−^ (pH = 10) | H_2_O + FeO_4_^2−^ → | 1.62 × 10^−2^ s^−1^ | 42.8 s | ^[13]^ |
| HSO_3_^−^ | H^+^ + HSO_3_^−^ → SO_2_ + H_2_O | 11.5 s^−1^ | 0.06 s | ^[14]^ |
| CO_2_ | CO_2_ + H_2_O→ H^+^ + HCO_3_^−^ | 2.75 × 10^2^ s^−1^ | 0.0025 s | ^[15]^ |
| Fe(IV) (pH = 1) | (H_2_^18^O)_5_Fe(^16^O)^2+^ ↔ (H_2_^18^O)_4_Fe(^16^OH)(^18^OH)^2+^ ↔ (H_2_^18^O)_4_(H_2_^16^O)Fe(^18^O)^2+^ ↔ (H_2_^18^O)_5_Fe(^18^O)^2+^ (oxo–hydroxo tautomerism mechanism) | 1.4 × 10^3^ s^−1^ | 0.0005 s | ^[16]^ |
| BrO_3_^−^ | 2 H^+^ + BrO_3_^−^ ↔ H_2_BrO_3_^+^  H_2_BrO_3_^+^ ↔ BrO_2_^+^ + H_2_O | *R* = *k* × [H^+^]^2^ × [BrO_3_^−^]  6.6 × 10^−3^ M^−2^ s^−1^ | Associated with initial concentration | ^[17]^ |
| ClO_3_^−^ | 2 H^+^ + ClO_3_^−^↔ H_2_ClO_3_^+^  H_2_ClO_3_^+^ ↔ ClO_2_^+^ + H_2_O | *R* = *k* × [H^+^]^2^ × [ClO_3_^−^]  8.9 × 10^−4^ M^−2^ s^−1^ | Associated with initial concentration | ^[18]^ |

**Table S4.** Results of ^16^O/^18^O exchanges.^[19]^ General conditions: acetonitrile : H_2_^18^O = 30:70 with the addition of 1% trifluoroacetic acid for 24 h. The red label means the exchangeable oxygen atoms.

| No. | Compound | Number of observed changes | | Structure | Maximum possible number of exchanges | Number of oxygen atoms |
| --- | --- | --- | --- | --- | --- | --- |
|  |  | At 37 °C | At 90 °C |  |  |  |
| 1 | Acemetacin | 2 | 2 |  | 2 | 6 |
| 2 | Climbazole | 1 | 1 |  | 1 | 2 |
| 3 | Norfloxacin | 1 | 2 |  | 3 | 3 |
| 4 | Acebutolol | 1 | 1 |  | 1 | 4 |
| 5 | Ketoprofen | 3 | 3 |  | 3 | 3 |
| 6 | Indapamide | 0 | 0 |  | 0 | 3 |
| 7 | Androstenedione | 2 | 2 |  | 2 | 2 |
| 8 | Ofloxacin | 1 | 1 |  | 2 () | 4 |
| 9 | Caffeine | 0 | 0 |  | 0 | 2 |
| 10 | Benzbromarone | 1 | 1 |  | 1 | 3 |
| 11 | Prednisolone | 2 | 2 |  | 2 | 5 |
| 12 | Nifedipine | 0 | 0 |  | 0 | 0 |
| 13 | Serotonin | 0 | 0 |  | 0 | 1 |
| 14 | Warfarin | 1 | 1 |  | 1 | 4 |
| 15 | Piroxicam | 0 | 0 |  | 0 | 4 |
| 16 | Doxorubicin | 1 | Degraded |  | 3 | 11 |
| 17 | Oxyfedrine | 1 | Degraded |  | 2 | 3 |
| 18 | Bumetanide | 0 | 2 |  | 2 | 5 |

**Table S5.** Relative yield of radicals formed in reactions with ^•^OH. Data from the reference.^[20]^

| Compound | Radical (α−C) | Relative yield | Radical (β−C) | Relative yield | Radical (O/N centered) | Relative yield |
| --- | --- | --- | --- | --- | --- | --- |
| CH_3_CH_2_OH | CH_3_^•^CHOH | 0.89 | ^•^CH_2_CH_2_OH | 0.07 | CH_3_CH_2_O^•^ | 0.04 |
| CD_3_CD_2_OH | CD_3_^•^CDOH | 0.88 | ^•^CD_2_CD_2_OH | 0.04 | CD_3_CD_2_O^•^ | 0.08 |
| CH_3_NH_2_ | ^•^CH_2_NH_2_ | 0.37 |  |  | CH_3_^•^NH | 0.63 |
| CD_3_NH_2_ | ^•^CD_2_NH_2_ | 0.26 |  |  | CD_3_^•^NH | 0.74 |

**Table S6.** Total and partial KIE for H/D abstraction reaction by ^•^OH. Data from the reference.^[20]^

| Compound | KIE(total) | KIE(α−C) | KIE(β−C) | KIE(O/N centered) |
| --- | --- | --- | --- | --- |
| CH_3_CH_2_OH/CD_3_CD_2_OH | 1.93 | 1.96 | 3.4 | 1.0 |
| CH_3_NH_2_/CD_3_NH_2_ | 1.31 | 1.86 |  | 1.11 |

**Table S7.** Second-Order Rate Constants for Reactions of Various Organics with ^1^O_2_. Data from the reference.^[21]^

| Compound | Rate constants with ^1^O_2_ (M^−1^ s^−1^) | Compound | Rate constants with ^1^O_2_ (M^−1^ s^−1^) | Compound | Rate constants with ^1^O_2_ (M^−1^ s^−1^) |
| --- | --- | --- | --- | --- | --- |
| tert-butyl alcohol | 1.8 × 10^3^ | phenol | 2.6 × 10^6^ | phenol(deprotonated) | 1.4 × 10^8^ |
| methanol | 3.0 × 10^3^ | 4-chlorophenol | 6 × 10^6^ | histidine | 1.5 × 10^8^ |
| sulfamethoxazole | 2.0 × 10^4^ | clofibric acid | 6.6 × 10^6^ | 4-chlorophenol (deprotonated) | 1.7 × 10^8^ |
| bisphenol A | 3.0 × 10^5^ | furfuryl alcohol | 1.2 × 10^8^ | bisphenol A (deprotonated) | 2.0 × 10^8^ |

**Table S8.** Summary of the reaction information and the KSIE in alleged ^1^O_2_-involved system.

| Catalysts | Oxidants | Contaminants | Other conditions | D_2_O concentration (%) | Dominant ROS | KSIE(*k*(D_2_O)/*k*(H_2_O)) | Reference |
| --- | --- | --- | --- | --- | --- | --- | --- |
| Heat | [PMS] = 0.1 mM | [sulfadiazine] = 5 μM | T = 70 °C, pH =5.7 | 100 | ^1^O_2_ | 1.09 | ^[22]^ |
| Alkaline | [PMS] = 0.5 mM | [TMDS] =20 μM | pH =5, 7 and 9 | 100 | ^1^O_2_ | 1.0 (pH 5); 1.43 (pH 7);  2 (pH 9) | ^[23]^ |
| [Fe_3_O_4_@CuO_0.8_] = 0.02 g/L | [PMS] = 0.04 mM | [triclosan] = 20 μM | T = 25 °C, pH = 7 | 100 | ^1^O_2_ | 1.38 | ^[24]^ |
| [Zn-Fe double oxides] = 0.3 g/L | [PMS] = 0.97 mM | [bisphenol A] = 87.6 μM | pH = 5.2 | 100 | ^1^O_2_ | 1.12 | ^[25]^ |
| [FeP] = 0.2 g/L | Air aeration | [ibuprofen] = 48.5 μM | [tripolyphosphate] = 2 mM, pH = 6.0 | 50 | ^1^O_2_ | 1.57 | ^[26]^ |
| [CuO/CNTs] = 0.2 g/L | [PMS] = 0.65 mM | [tetracycline] =22.5 μM | / | 100 | SO_4_^•−^, ^•^OH, O_2_^•−^ and ^1^O_2_ | 1.27 | ^[27]^ |
| [FeCo alloy@N-doped carbon yolk-shell nanoreactors] = 0.02 g/L | [PMS] = 0.65 mM | [bisphenol A] = 87.6 μM | T = 25 °C, pH = 7 | 100 | SO_4_^•−^, ^•^OH and ^1^O_2_ | 1.21 | ^[28]^ |
| [β-MnO_2_] = 0.4 g/L | [PDS] = 4 mM | [phenol] =100 μM | 1 mM borate buffer for pH 6.5 | 100 | ^1^O_2_ | 1.5 | ^[29]^ |
| [Fe_3_O_4_@UiO-66] = 1g/L | [H_2_O_2_] = 10 mM | [bisphenol A] = 100 μM | pH = 5 | 80 | ^•^OH and ^1^O_2_ | 1.28 | ^[30]^ |
| [Ni_3_ZnC_0.7_ alloy@N-doped graphite] = 0.2 g/L | [PMS] = 0.244 mM | [bisphenol A] = 87.6 μM | T = 30 °C, pH = 6.5 | 100 | ^1^O_2_ | 1.10 | ^[31]^ |
| [FeMg@NCNT] = 0.1 g/L | [PMS] = 1 mM | [sulfamethoxazole] = 19.7 μM | T = 25 °C, pH = 6.4 | 50 | ^1^O_2_ | 2.29 | ^[32]^ |
| [goethite-MoS_2_] = 0.05 g/L | [PMS] = 1 mM | [tetracycline] = 67.5 μM | pH = 7 | 100 | ^•^OH and ^1^O_2_ | 1.36 | ^[33]^ |
| [FeCu_1.5_O_3_@zeolite hollow sphere] = 0.5 g/L | [PMS] = 1 mM | [bisphenol A] = 87.6 μM | T = 25 °C, pH = 7 | 100 | ^1^O_2_ | 1.26 | ^[34]^ |
| [Carbon nitride@ graphitic biochar] = 1 g/L | [PMS] = 1 mM | [bisphenol A] = 35 μM | T = 25 °C, pH = 6.38 | 50 | ^1^O_2_ | 1.26 | ^[35]^ |
| Self-catalysis | [PMS] = 0.1 mM | [thiacloprid] = 35 μM | pH = 7 | 50 | ^1^O_2_ | 1.25 | ^[36]^ |
| [La_0.6_Sr_0.4_Co_0.8_Fe_0.2_O_2.75-δ_Cl_0.25_] = 0.1 g/L | [PMS] = 3.25 mM | [bisphenol A] = 35 μM | pH = 7 | 100 | SO_4_^•−^ and ^1^O_2_ | 1.45 | ^[37]^ |
| Electro-activation | [PMS] = 1 mM | [aniline] = 53.7 μM | Applied potential = 1.2 V and pH = 6.5 | 100 | Anodic oxidation and ^1^O_2_ | 1.86 | ^[38]^ |
| [dicarbonyl bacterial cellulose] = 0.05 g/L | [PMS] = 2 mM | [sulfamethoxazole] = 100 μM | T = 25 °C, pH = 6.5 | 100 | SO_4_^•−^, ^•^OH and ^1^O_2_ | 7.29 | ^[39]^ |
| [FeCoNC singlet-atom catalysts] = 0.3 g/L | [PMS] = 2 mM | [tetracycline] = 180 μM | T = 25 °C, pH = 6.5 | 100 | SO_4_^•−^ and ^1^O_2_ | 1.04 | ^[40]^ |
| [N-CNTs] = 0.05 g/L | [PMS] = 0.1 mM | [phenol] = 10 μM | T = 25 °C, pH = 4.3 | 50 | ^1^O_2_ | 1.68 | ^[41]^ |
| [N-doped carbon] = 0.9 g/L | [PMS] = 1.5 mM | [sulfamethoxazole] = 100 μM | T = 25 °C, pH = 6.5 | 100 | ^1^O_2_ and direct electron transfer | 1.23 | ^[42]^ |
| [sulfonated cobalt (II) phthalocyanine] = 0.1 mM | [PMS] = 0.5 mM | [levofloxacin] = 138.4 μM | T = 25 °C, pH = 4 | 100 | SO_4_^•−^, Co(IV) and ^1^O_2_ | 1.33 | ^[43]^ |
| Heat | [PMS] = 1.5 mM | [acetaminophen] = 10 μM | T = 75 °C, pH = 9.0, [Na_2_B_4_O_7_] = 10 mM | 50 | ^•^OH and ^1^O_2_ | 1.05 | ^[44]^ |
| [Co/N co-doped biochar] = 0.16 g/L | [PMS] = 1 mM | [tetracycline] = 45 μM | T = 25 °C, pH = 5.6 | 100 | SO_4_^•−^ and ^1^O_2_ | 1.60 | ^[45]^ |
| [CoFe alloy@N-doped carbon] = 0.05 g/L | [PMS] = 0.2 mM | [norfloxacin] = 15 μM | T = 25 °C, pH = 6.0 | 100 | SO_4_^•−^ and ^1^O_2_ | 1.10 | ^[46]^ |
| Heat | [PMS] = 1 mM | [acetaminophen] = 10 μM | T = 75 °C, pH = 6.52, [Na_2_CO_3_] = 3 mM | 50 | ^1^O_2_ | 1.14 | ^[47]^ |
| [B,N-decorated carbocatalyst] = 0.15 g/L | [PMS] = 2 mM | [bisphenol A] = 87.6 μM | T = 25 °C, pH = 7.0 | 100 | SO_4_^•−^, ^•^OH and ^1^O_2_ | 2.79 | ^[48]^ |
| [UiO-66-X] = 1 g/L | O_2_ | [Acid Orange 7] = 100 μM | T = 25 °C, pH = 4.5, visible-light irradiation with a 300 W  Xenon lamp | 100 | ^1^O_2_ | 1.68 | ^[49]^ |
| Calcined ZIF-67 membrane | [PMS] = 1 mM | [bisphenol A] = 44 μM | T = 25 °C flow rate = 1.5 mL/min | 100 | SO_4_^•−^, ^•^OH and ^1^O_2_ | 1.40 | ^[50]^ |
| [Nitrogen-doped graphitic carbon] = 0.1 g/L | [PMS] = 0.65 mM | [bisphenol A] = 87.6 μM | T = 25 °, pH = 6.5 | 100 | SO_4_^•−^, ^•^OH and ^1^O_2_ | 1.38 | ^[51]^ |
| Bicarbonate | [PDS] = 5 mM | [acetaminophen] = 10 μM, [furfuryl alcohol] = 20 μM | T = 25 °C, pH = 8.3, CO_2_ saturated | 50 | ^1^O_2_ and peroxymonocarbonate | 1.20 (acetaminophen); 1.49 (furfuryl alcohol) | ^[52]^ |
| / | [PI] = [H_2_O_2_] = 2 mM | [furfuryl alcohol] = 100 μM | T = 25 °C, pH = 6 | 100 | ^•^OH and ^1^O_2_ | 1.16 | ^[53]^ |
| Visible light | [PDS] = 5 mM | [Atrazine] = 10 μM | T = 25 °C, pH = 7.0, [Na_2_HPO_4_] = 10.0 mM, | 100 | SO_4_^•−^, ^•^OH and ^1^O_2_ | 2.5 | ^[54]^ |
| [Zn_4_Co_1_−N−C] = 0.1 g/L | [PMS] = 0.65 mM | [phenol] = 212.5 μM | T = 25 °C | 100 | ^1^O_2_ | 1.16 | ^[55]^ |
| [Nitrogen-doped carbon nanosheets] = 0.1 g/L | [PMS] = 2 mM | [bisphenol A] = 100 μM | T = 30 °C, pH = 7.0 | 100 | ^1^O_2_ | 2.40 | ^[56]^ |
| [Graphitized nanodiamonds] = 0.1 g/L | [PMS] = 0.5 mM | [4-chlorophenol] = 389 μM | T = 25 °C | 100 | ^1^O_2_ and direct electron transfer | 1.68 | ^[57]^ |
| [Fe_0.15_Mn_0.85_O_2_] = 0.04 g/L | [PMS] = 0.5 mM | [bisphenol A] = 5 μM | T = 25 °C, pH = 10.5, [Na_2_B_4_O_7_] = 5 mM | 100 | Direct electron transfer and ^1^O_2_ | 1.31 | ^[58]^ |
| [Fe_5_-NC] = 0.20 g/L | [O_3_] = 3.5 mg/L, flow rate = 0.2 L/min | [p-hydroxybenzoic acid] = 362 μM | T = 25 °C | 50 | surface-adsorbed atomic oxygen and ^1^O_2_ | 1.49 | ^[59]^ |
| [Manganese dioxide octahedral molecular sieve] = 0.1 g/L | [PMS] = 1.5 mM | [furfuryl alcohol] = 100 μM | T = 25 °C, pH = 6.2 | 75, 90, 100 | Direct electron transfer and ^1^O_2_ | 1.42 (75%); 1.48 (90%); 0.04 (100%) | ^[60]^ |

**Table S9.** Summary of the reaction information and the KSIE in non ^1^O_2_-involved system.

| Catalysts | Oxidants | Contaminants | Other conditions | D_2_O concentration (%) | Dominant ROS | KSIE(*k*(D_2_O)/*k*(H_2_O)) | Reference |
| --- | --- | --- | --- | --- | --- | --- | --- |
| [Carbon nitride nanosheet@ single atom FeN_5_] = 0.08 g/L | [PMS] = 0.65 mM | [Sulfamethoxazole] = 79 μM | T = 25 °C, pH =5.6 | 100 | Non-radical surface contact oxidation | 0.68 | ^[61]^ |
| [Fe-Zr bimetallic oxide] = 0.5 g/L | [H_2_O_2_] = 0.5 mM | [bisphenol A] = 0.1 mM | pH = 7 | 80 | Fe(IV) and ^•^OH | 1.03 | ^[62]^ |
| [nano NiO-ZnO] = 0.05 g/L | [PMS] = 0.1 mM | [bisphenol A] = 43.8 μM | T = 25 °C, pH = 6.5 | 100 | Surface bounded SO_4_^•−^ and ^•^OH | 1.02 | ^[63]^ |
| [magnetic Mn-Fe oxycarbide] = 0.125 g/L | [PMS] = 2 mM | [butyl paraben] = 25.7 μM | pH = 6.8 | 100 | SO_4_^•−^ and ^•^OH | 0.45 | ^[64]^ |
| [CoWO_4_] = 0.5 g/L | [PMS] = 1 mM | [4-chlorophenol] = 100 μM | pH = 8.0 | 100 | SO_4_^•−^ | 0.92 | ^[65]^ |
| [α-Fe_2_O_3_] = 5 g/L | [PMS] = 1 mM | [Furfuryl alcohol] = [phenol] = 100 μM | pH = 3.3 | 100 | Fe(IV) | 0.93 (Furfuryl alcohol); 0.734 (phenol) | ^[66]^ |
| [hemin] = 0.5 g/L | [PMS] = 1 mM | [bisphenol A] = 4 μM | T = 25 °C, pH = 3.2 | 100 | Fe(V) | 1.36 | ^[67]^ |
| [MoSe_2_] = 0.3 g/L | [PMS] = 1.2 mM | [carbamazepine] = 8.5 μM | pH 4.0, visible-light irradiation | 100 | O_2_^•−^ | 0.04 | ^[68]^ |
| [Phenylhydrazine modified carbon quantum dots] = 4 g/L | [PMS] = 0.4 mM | [methylene blue] = 31.3 μM | T = 25 °C, LED light | 100 | O_2_^•−^ and photogenerated holes | 1.14 | ^[69]^ |
| [MnO_x_] = 0.1 g/L | [PMS] = 0.7 mM | [phenol] = 53 α-MnO_2_), 42.5 (β-MnO_2_), 106 (γ-MnO_2_), 53 (AMO), and 42.5μM (Mn_2_O_3_) | T = 23 °C | 100 | nonradical electron-transfer pathway | 0.87 (α-MnO_2_); 0.45 (β-MnO_2_); 0.71 (γ-MnO_2_); 0.72 (amorphous MnO_2_); 0.82 (Mn_2_O_3_) | ^[70]^ |
| [Hollow hemispherical Si-doped TiO_2_] = 0.1 g/L | [PMS] = 1 mM | [carbamazepine] = 42.3 μM | T = 20 °C, light | 100 | ^•^OH (dominant), SO_4_^•−^  and holes | 1.40 | ^[71]^ |
| [N/S co-doped Fe-C composite] = 0.2 g/L | [PMS] = 0.8 mM | [ciprofloxacin] = 60.4 μM | T = 25 °C, pH = 6 | 100 | Fe(IV) | 0.60 | ^[72]^ |
| [Oily sludge derived carbocatalysts] = 0.2 g/L | [PMS] = 0.925 mM | [phenol] = 531 μM | / | 100 | electron-transfer mechanism | 0.79 | ^[73]^ |
| [Mn doped C_3_N_4_] = 1 g/L | [PMS] = 1 mM | [bisphenol A] = 43.8 μM | T = 25 °C, pH = 6.8, visible light | 100 | electron-transfer mechanism | 1.10 | ^[74]^ |
| [Cu_2_O cubic crystals] = 0.2 g/L | [PMS] = 1 mM | [bisphenol A] = 44 μM | pH = 7.2 0.5 mM NaHCO_3_ buffer | 100 | surface-confined metastable intermediate | 1.05 | ^[75]^ |
| [Amorphous MnO_x_] = 0.4 g/L | [PMS] = 2 mM | [acid organic II] = 57.1 μM | pH = 7 | 100 | electron-transfer mechanism | 0.99 | ^[76]^ |
| [Mn-incorporated biochar] = 0.2 g/L | [PMS] = 2 mM | [acid organic 7] = 53.7 μM | T = 25 °C, pH = 7 | 100 | electron-transfer mechanism | 0.86 | ^[77]^ |
| [natural Mn-containing minerals] = 1 g/L | [PMS] = 0.4 mM | [bisphenol AF] = 10 μM | T = 23 °C, pH = 7.5 | 50 | electron-transfer mechanism | 0.82 | ^[78]^ |
| [MnO_x_-N-biochar] = 0.2 g/L | [PMS] = 1.6 mM | [acid organic 7] = 53.7 μM | T = 25 °C, pH = 6 (1 mM phosphat) | 100 | electron-transfer mechanism (dominant); SO_4_^•−^, ^•^OH and ^1^O_2_ (secondary) | 0.90 | ^[79]^ |
| [Hydrogenated nanodiamonds] = 0.1 g/L | [PDS] = 1 mM | [4-chlorophenol] = 10 μM | T = 25 °C, pH = 7 | 100 | electron-transfer mechanism | 0.9 | ^[80]^ |
| [Fe-N@biochar] = 0.5 g/L | [PDS] = 1.8 mM | [tetracycline] = 11.3 μM | / | 100 | SO_4_^•−^, ^•^OH (dominant); O_2_^•−^, ^1^O_2_ and electron transfer (secondary) | 0.30 | ^[81]^ |
| [Metal free biochar] = 0.3 g/L | [PI] = 0.5 mM | [bisphenol A] = 21.9 μM | pH = 7 | 100 | electron-transfer mechanism | 0.71 | ^[82]^ |
| Phosphate | [PMS] = 5 mM | [atrazine] = 10 μM | T = 25 °C, pH = 7.0, [Na_2_HPO_4_] = 10 mM | 100 | SO_4_^•−^ (O_2_^•−^ and ^1^O_2_ were generated but didn’t contribute to degradation) | 1 | ^[83]^ |
| [Fe-N-porous carbon] = 0.03 g/L | [PMS] = 0.3 mM | [sulfamethoxazole] = 39.5 μM | T = 25 °C, pH = 6.0 | 20 | SO_4_^•−^ and O_2_^•−^ (^1^O_2_ were generated but didn’t contribute to degradation) | 0.51 | ^[84]^ |
| [Hollow carbon nanospheres] = 0.01 g/L | [PDS] = 1 mM | [sulfamethoxazole] = 7.9 μM | / | 100 | electron-transfer mechanism | 0.95 | ^[85]^ |
| [N-CNT] = 0.1 g/L | [PMS] = 0.2 mM | [phenol] = 10 μM | T = 25 °C, pH = 4.0 | 100 | electron-transfer mechanism | 1.03 | ^[86]^ |
| [Ultrathin 2D Fe_3_O_4_ nanosheets] = 0.2 g/L | [PMS] = 1.6 mM | [bisphenol A] = 87.6 μM | T = 25 °C, pH = 6.0 | 100 | SO_4_^•−^, ^•^OH and electron-transfer | 1.05 | ^[87]^ |
| [Co−N−CNTs] = 0.1 g/L | [PMS] = 1 mM | [sulfamethoxazole] = 39.5 μM | T = 25 °C, pH = 6.8 | 100 | electron-transfer mechanism | 1.02 (Co−N−CNTs); 1.17 (N−CNTs) | ^[88]^ |
| [Fe_0.15_Mn_0.85_O_2_] = 0.04 g/L | [PMS] = 0.5 mM | [bisphenol A] = 5 μM | T = 25 °C, pH = 5, [acetate] = 20 mM | 100 | Direct electron transfer | 0.93 | ^[58]^ |
| [CuO] = 0.2 g/L | [PMS] = 0.2 mM | [4-chlorophenol] = 77.8 μM | T = 25 °, pH = 7 | 100 | Cu(III) | 0.37 | ^[89]^ |
| [Pd] = 0.25 g/L | [PMS] = 0.25 mM | [4-chlorophenol] = 100 μM | T = 25 °C, pH = 7.5, [borate buffer] = 5 mM | 100 | electron transfer and surface bounded radicals | 0.98 | ^[90]^ |
| [Co−N_4_−C] = 0.1 g/L | [PMS] = 0.5 mM | [metronidazole] = 58.4 μM [bisphenol A] = 87.6 μM | T = 25 °C | 100 | SO_4_^•−^, ^•^OH and electron-transfer | 0.33 (metronidazole); 0.98 (bisphenol A) | ^[91]^ |
| / | [O_3_] = 3.5 mg/L, flow rate = 0.2 L/min | [p-hydroxybenzoic acid] = 362 μM | T = 25 °C | 50 | Direct O_3_ oxidation | 1.13 | ^[59]^ |
| [CNT] = 0.1 g/L | [PMS] = 1 mM | [4-chlorophenol] = 150 μM | T = 25 °C, pH =7.0, [phosphate buffer] = 1 mM | 100 | electron-transfer | 0.93 | ^[92]^ |
| [Co_3_O_4_] = 1 g/L | [chlorite] = 0.1 mM | [cephalexin] = 28.8 μM | T = 25 °C | 100 | Co(IV) and ClO_2_ | 1.13 | ^[93]^ |
| [Single-Atom Iron Anchored Tubular g-C_3_N_4_] = 0.15 g/L | [PAA] = 0.5 mM | [bisphenol A] = 43.8 μM | T = 25 °C | 100 | Fe(IV) and organic radicals | 1.02 | ^[94]^ |
| [Co_3_O_4_] = 1 g/L | [chlorite] = 0.1 mM | [cephalexin] = 28.8 μM | T = 25 °C | 100 | Co(IV) and ClO_2_ | 1.13 |  |
| [Co^2+^] = 0.15 mM | [PMS] = 0.15 mM | [methyl phenyl sulfoxide] = 500 μM; [sulfamethoxazole] = 27 μM | T = 25 °C, pH = 3 | 100 | Co(IV) | 0.72 (methyl phenyl sulfoxide and sulfamethoxazole) | ^[95]^ |
| [Fe^2+^] = 0.25 mM | [PI] = 0. 5 mM | [methyl phenyl sulfoxide] = 500 μM; | T = 25 °C, pH = 3 | 100 | Fe(IV) | 1.0 | ^[96]^ |
| [Amorphous MnO_x_@polymer] = 0.2 g/L | [PMS] = 0.25 mM | [bisphenol A] = 25 μM | T = 25 °C, pH = 3.8 | 100 | high-spin MnIII-peroxy complex | 0.37 | ^[97]^ |
| [Amorphous ZrO_2_] = 0.5 g/L | [PMS] = 0.1 mM | [carbamazepine] = 42.3 μM | T = 25 °C, pH = 3 | 90 | Zr(IV)-PMS* complex | 0.71 | ^[98]^ |
| [CNT] = 0.1 g/L | [PMS] = 1 mM | [furfuryl alcohol] = [4-chlorophenol] = 50 μM | T = 25 °C, [phosphate buffer] = 1 mM; pHi = 7.0 | 100 | electron-transfer | 1 (furfuryl alcohol); 0.85 (4-chlorophenol) | ^[99]^ |

**Table S10.** Summary of the reaction information and the KSIE in benchmark ^1^O_2_-involved system.

| Catalysts | Oxidants | Contaminants | Other conditions | D_2_O concentration (%) | Dominant ROS | KSIE(*k*(D_2_O)/*k*(H_2_O)) | Reference |
| --- | --- | --- | --- | --- | --- | --- | --- |
| [Rose Bengal] = 50 μM | O_2_ | [furfuryl alcohol] = 50 μM | T = 25 °C, [phosphate buffer] = 1 mM; pHi = 7.0, light irradiation | 100 | ^1^O_2_ | 3.4 | ^[99]^ |
| [C_60_/polyvinylpyrrolidone] = [C_60_/TX surfactant] = 0.005 g/L | O_2_ | [furfuryl alcohol] = 850 μM | T = 25 °C, [phosphate buffer] = 10 mM; pH = 7.0, UV irradiation | 70 | ^1^O_2_ | 2.5 (C_60_/polyvinylpyrrolidone); 1.8 (C_60_/TX surfactant) | ^[100]^ |
| Fluka humic  acid (DOC = 16 mg/L) | O_2_ | [furfuryl alcohol] = 100 μM | pH = 7, irradiated in the merry-go-round reactor with  a high-pressure Hg lamp filtered through  solidex borosilicate glass | 40 and 60 | ^1^O_2_ | 1.72 (40% D_2_O) and 2.34 (60% D_2_O) | ^[101]^ |
| Rose Bengal | O_2_ | [acid organic 7] = 53.7 μM | T = 25 °C, [phosphate buffer] = 1 mM; pH = 6.0 | 100 | ^1^O_2_ | 3.69 | ^[79]^ |
| [perinaphthenone] = 0.8 μM | O_2_ | Natural cyanopeptide | T = 12 °C, pH = 11.6, UVA irradiation | 93-100 | ^1^O_2_ | 2.85 (Anabaenopeptin A); 5.80 (Anabaenopeptin 871); 2.22 (Oscillamide Y); 1.90 (MC-YR) | ^[102]^ |
| [perinaphthenone] = 0.77 μM | O_2_ | [diarylamine  pharmaceutical] = 5μM | [phosphate buffer] = 5 mM; pH = 8, UVA light (365 nm) | 81-94 | ^1^O_2_ | 1.51 (flufenamic acid); 1.49 (mefenamic acid) | ^[103]^ |

**References**

[1] E. M. Simmons, J. F. Hartwig, *Angew. Chem. Int. Ed.* **2012**, *51*, 3066-3072.

[2] a) V. L. Lobachev, E. S. Rudakov, Y. V. Matvienko, L. K. Volkova, *Theor. Exp. Chem.* **2008**, *44*, 37-41; b) J. J. Pignatello, D. Liu, P. Huston, *Environ. Sci. Technol.* **1999**, *33*, 1832-1839.

[3] A. T. Droege, F. P. Tully, *J. Phys. Chem.* **1987**, *91*, 1222-1225.

[4] J. Kim, R. G. Harrison, C. Kim, L. Que, *J. Am. Chem. Soc.* **1996**, *118*, 4373-4379.

[5] F.-D. Kopinke, A. Georgi, *J. Phys. Chem. A* **2017**, *121*, 7947-7955.

[6] T. Gierczak, M. K. Gilles, S. Bauerle, A. R. Ravishankara, *J. Phys. Chem. A* **2003**, *107*, 5014-5020.

[7] T. Guarr, E. Buhks, G. McLendon, *J. Am. Chem. Soc.* **1983**, *105*, 3763-3767.

[8] M. T. Bender, R. E. Warburton, S. Hammes-Schiffer, K.-S. Choi, *ACS Catal.* **2021**, *11*, 15110-15124.

[9] a) E. C. M. Tse, T. T. H. Hoang, J. A. Varnell, A. A. Gewirth, *ACS Catal.* **2016**, *6*, 5706-5714; b) Y. Lin, K.-H. Wu, Q. Lu, Q. Gu, L. Zhang, B. Zhang, D. Su, M. Plodinec, R. Schlögl, S. Heumann, *J. Am. Chem. Soc.* **2018**, *140*, 14717-14724.

[10] Y. Li, B. Wen, C. Yu, C. Chen, H. Ji, W. Ma, J. Zhao, *Chem. Eur. J.* **2012**, *18*, 2030-2039.

[11] A. Okumura, M. Kitani, Y. Toyomi, N. Okazaki, *Bulletin of the Chemical Society of Japan* **1980**, *53*, 3143-3148.

[12] M. Anbar, S. Guttmann, *J. Am. Chem. Soc.* **1961**, *83*, 781-783.

[13] H. Goff, R. K. Murmann, *J. Am. Chem. Soc.* **1971**, *93*, 6058-6065.

[14] D. A. Horner, R. E. Connick, *Inorg. Chem.* **2003**, *42*, 1884-1894.

[15] D. J. Poulton, H. W. Baldwin, *Can. J. Chem.* **1967**, *45*, 1045-1050.

[16] O. Pestovsky, A. Bakac, *Inorg. Chem.* **2006**, *45*, 814-820.

[17] T. C. Hoering, R. C. Butler, H. O. McDonald, *J. Am. Chem. Soc.* **1956**, *78*, 4829-4831.

[18] T. C. Hoering, F. T. Ishimori, H. O. McDonald, *J. Am. Chem. Soc.* **1958**, *80*, 3876-3879.

[19] S. Osipenko, A. Zherebker, L. Rumiantseva, O. Kovaleva, E. N. Nikolaev, Y. Kostyukevich, *J. Am. Soc. Mass Spectrom.* **2022**, *33*, 390-398.

[20] M. Bonifačić, D. A. Armstrong, I. Štefanić, K.-D. Asmus, *J. Phys. Chem. B* **2003**, *107*, 7268-7276.

[21] a) W. Ren, C. Cheng, P. Shao, X. Luo, H. Zhang, S. Wang, X. Duan, *Environ. Sci. Technol.* **2022**, *56*, 78-97; b) X. Lu, W. Qiu, J. Ma, H. Xu, D. Wang, H. Cheng, W. Zhang, X. He, *Chem. Eng. J.* **2020**, *401*, 126128.

[22] Y. Qin, H. Li, J. Ma, *Chem. Eng. J.* **2023**, *451*, 138814.

[23] L. Shen, Z. Chen, J. Kang, P. Yan, J. Shen, B. Wang, S. Zhao, L. Bi, S. Wang, Y. Cheng, *J. Hazard. Mater.* **2022**, *428*, 128191.

[24] S. Shao, X. Li, Z. Gong, B. Fan, J. Hu, J. Peng, K. Lu, S. Gao, *Chem. Eng. J.* **2022**, *438*, 135474.

[25] L. Zhang, B. Zhang, Y. Liu, Z. Wang, J. Hussain Shah, R. Ge, W. Zhou, S. Kubuki, J. Wang, *Chem. Eng. J.* **2023**, *454*, 140103.

[26] N. Zheng, X. He, R. Hu, R. Wang, Q. Zhou, Y. Lian, Z. Hu, *Appl. Catal. B: Environ.* **2022**, *307*, 121157.

[27] W. Zhang, Z. Li, R. Luo, Q. Guo, F. Xu, F. Yang, M. Zhang, L. Jia, S. Yuan, *Sep. Purif. Technol.* **2023**, *306*, 122548.

[28] W. Zhang, M. Yang, H. Zhang, X. Yu, W. Zhang, A. T. S. Wee, X. Yan, J. Qi, J. Li, *Chem. Eng. J.* **2022**, *428*, 131080.

[29] S. Zhu, X. Li, J. Kang, X. Duan, S. Wang, *Environ. Sci. Technol.* **2019**, *53*, 307-315.

[30] Y. Yin, Y. Ren, J. Lu, W. Zhang, C. Shan, M. Hua, L. Lv, B. Pan, *Appl. Catal. B: Environ.* **2021**, *286*, 119943.

[31] J. You, C. Zhang, Z. Wu, Z. Ao, W. Sun, Z. Xiong, S. Su, G. Yao, B. Lai, *Chem. Eng. J.* **2021**, *415*, 128890.

[32] J. Zheng, Q. Lin, Y. Liu, X. Fan, K. Xu, Y. Ma, J. He, H. Fu, *Chem. Eng. J.* **2023**, *452*, 139233.

[33] X. Li, L. Wang, Y. Guo, W. Song, Y. Li, L. Yan, *Chem. Eng. J.* **2022**, *450*, 138104.

[34] Z. Lyu, M. Xu, J. Wang, A. Li, P. François-Xavier Corvini, *Chem. Eng. J.* **2022**, *433*, 133581.

[35] H. Wang, W. Guo, Q. Si, B. Liu, Q. Zhao, H. Luo, N. Ren, *Chem. Eng. J.* **2021**, *418*, 129504.

[36] T. Liu, D. Zhang, K. Yin, C. Yang, S. Luo, J. C. Crittenden, *Chem. Eng. J.* **2020**, *388*, 124264.

[37] Q. Yang, X. Niu, Y. Zhu, Y. Cui, Y. Chao, P. Liang, C. Zhang, S. Wang, *J. Hazard. Mater.* **2022**, *432*, 128686.

[38] F. Liu, Z. Wang, S. You, Y. Liu, *Appl. Catal. B: Environ.* **2023**, *320*, 121980.

[39] F. Liu, Y. Zhang, S. Wang, T. Gong, M. Hua, J. Qian, B. Pan, *Chem. Eng. J.* **2022**, *430*, 132767.

[40] B. Zhang, X. Li, P. A. Bingham, K. Akiyama, S. Kubuki, *Chem. Eng. J.* **2023**, *451*, 138574.

[41] S. Liu, S. Yin, Z. Zhang, L. Feng, Y. Liu, L. Zhang, *J. Hazard. Mater.* **2023**, *441*, 129905.

[42] W. Peng, J. Liao, Y. Yan, L. Chen, C. Ge, S. Lin, *Chem. Eng. J.* **2022**, *446*, 137407.

[43] Y. Wang, H. Shen, Z. Shi, Q. Xing, Y. Pi, *Chem. Eng. J.* **2023**, *455*, 140671.

[44] J. Li, J. Zou, S. Zhang, H. Cai, Y. Huang, J. Lin, Q. Li, B. Yuan, J. Ma, *Water Res.* **2022**, *224*, 119095.

[45] H. Zhu, A. Guo, S. Wang, Y. Long, G. Fan, X. Yu, *Chem. Eng. J.* **2022**, *450*, 138428.

[46] D. Ding, S. Yang, L. Chen, T. Cai, *Chem. Eng. J.* **2020**, *392*, 123725.

[47] H. Cai, J. Zou, J. Lin, Q. Li, J. Li, Y. Huang, H. Yang, B. Yuan, J. Ma, *Chem. Eng. J.* **2022**, *449*, 137765.

[48] Y. Wan, W. Zhang, X. Han, L. Zhou, H. Zhen, C. Wu, Q. Yu, G. Xiu, *J. Hazard. Mater.* **2022**, *430*, 127832.

[49] W. Huang, X. Wang, W. Zhang, S. Zhang, Y. Tian, Z. Chen, W. Fang, J. Ma, *Appl. Catal. B: Environ.* **2020**, *273*, 119087.

[50] M. Li, S. You, X. Duan, Y. Liu, *Appl. Catal. B: Environ.* **2022**, *312*, 121419.

[51] R. Luo, M. Li, C. Wang, M. Zhang, M. A. Nasir Khan, X. Sun, J. Shen, W. Han, L. Wang, J. Li, *Water Res.* **2019**, *148*, 416-424.

[52] M. Jiang, J. Lu, Y. Ji, D. Kong, *Water Res.* **2017**, *116*, 324-331.

[53] Y. Kim, H. Lee, H. Oh, Z. Haider, J. Choi, Y.-U. Shin, H.-i. Kim, J. Lee, *Environ. Sci. Technol.* **2022**, *56*, 5763-5774.

[54] Y. Wen, C.-H. Huang, D. C. Ashley, D. Meyerstein, D. D. Dionysiou, V. K. Sharma, X. Ma, *Environ. Sci. Technol.* **2022**, *56*, 2626-2636.

[55] Y. Yao, C. Wang, X. Yan, H. Zhang, C. Xiao, J. Qi, Z. Zhu, Y. Zhou, X. Sun, X. Duan, J. Li, *Environ. Sci. Technol.* **2022**, *56*, 8833-8843.

[56] Y. Gao, Z. Chen, Y. Zhu, T. Li, C. Hu, *Environ. Sci. Technol.* **2020**, *54*, 1232-1241.

[57] P. Shao, Y. Jing, X. Duan, H. Lin, L. Yang, W. Ren, F. Deng, B. Li, X. Luo, S. Wang, *Environ. Sci. Technol.* **2021**, *55*, 16078-16087.

[58] K. Z. Huang, H. Zhang, *Environ. Sci. Technol.* **2019**, *53*, 12610-12620.

[59] T. Ren, M. Yin, S. Chen, C. Ouyang, X. Huang, X. Zhang, *Environ. Sci. Technol.* **2023**.

[60] R. Huang, P. Gao, J. Zhu, Y. Zhang, Y. Chen, S. Huang, G. Wang, Z. Yu, S. Zhao, S. Zhou, *Appl. Catal. B: Environ.* **2022**, *317*, 121753.

[61] C. Zhu, Y. Nie, F. Cun, Y. Wang, Z. Tian, F. Liu, *Appl. Catal. B: Environ.* **2022**, *319*, 121900.

[62] Y. Yin, M. Li, X. Li, W. Zhang, L. Lv, J. Wan, Y. Wang, *Chem. Eng. J.* **2023**, *454*, 140516.

[63] J. You, W. Sun, S. Su, Z. Ao, C. Liu, G. Yao, B. Lai, *Chem. Eng. J.* **2020**, *400*, 125915.

[64] J.-C. E. Yang, Y. Lin, H.-H. Peng, B. Yuan, D. D. Dionysiou, X.-D. Huang, D.-D. Zhang, M.-L. Fu, *Appl. Catal. B: Environ.* **2020**, *268*, 118549.

[65] A. Q. K. Nguyen, Y.-Y. Ahn, G. Shin, Y. Cho, J. Lim, K. Kim, J. Kim, *Appl. Catal. B: Environ.* **2023**, *324*, 122266.

[66] H. Kang, D. Lee, K.-M. Lee, H.-H. Kim, H. Lee, M. Sik Kim, C. Lee, *Chem. Eng. J.* **2021**, *426*, 130743.

[67] Y. Feng, Y. Li, B. Yang, Z. Yang, Y. Fan, K. Shih, H. Li, D. Wu, L. Zhang, *Chem. Eng. J.* **2021**, *420*, 130477.

[68] C. Dong, Z. Wang, Z. Ye, J. He, Z. Zheng, X. Gong, J. Zhang, I. M. C. Lo, *Appl. Catal. B: Environ.* **2021**, *296*, 120223.

[69] W. Han, D. Li, M. Zhang, H. Ximin, X. Duan, S. Liu, S. Wang, *J. Hazard. Mater.* **2020**, *395*, 122695.

[70] Y. Yang, P. Zhang, K. Hu, P. Zhou, Y. Wang, A. H. Asif, X. Duan, H. Sun, S. Wang, *Appl. Catal. B: Environ.* **2022**, *315*, 121593.

[71] Y. Zhou, H. Zhang, L. Wu, Y. Zhang, X. Wang, Z. Wu, *Chem. Eng. J.* **2023**, *457*, 141234.

[72] H. Chen, R. Chen, S. Yang, D. Ding, X. Li, X. Long, T. Zhao, Y. Du, M. Liu, J. Tan, Y. Chen, *Chem. Eng. J.* **2022**, *446*, 137257.

[73] W. Liu, C. Nie, W. Li, Z. Ao, S. Wang, T. An, *J. Hazard. Mater.* **2021**, *414*, 125552.

[74] Q. Si, W. Guo, B. Liu, H. Wang, S. Zheng, Q. Zhao, H. Luo, N. Ren, T. Yu, *Chem. Eng. J.* **2022**, *443*, 136399.

[75] H. Li, J. Tian, F. Xiao, R. Huang, S. Gao, F. Cui, S. Wang, X. Duan, *J. Hazard. Mater.* **2020**, *385*, 121518.

[76] S. Zhu, P. Xiao, X. Wang, Y. Liu, X. Yi, H. Zhou, *J. Hazard. Mater.* **2022**, *427*, 127938.

[77] B. Gao, S. Zhu, J. Gu, Y. Liu, X. Yi, H. Zhou, *J. Hazard. Mater.* **2022**, *431*, 128549.

[78] L. Wang, H. Xu, N. Jiang, S. Pang, J. Jiang, T. Zhang, *J. Hazard. Mater.* **2021**, *417*, 126152.

[79] M. M. Mian, G. Liu, B. Fu, Y. Song, *Appl. Catal. B: Environ.* **2019**, *255*, 117765.

[80] G. Gim, Z. Haider, S.-I. Suh, Y.-Y. Ahn, K. Kim, E.-J. Kim, H. Lee, H.-i. Kim, J. Lee, *Appl. Catal. B: Environ.* **2022**, *316*, 121589.

[81] S.-N. Zhuo, H. Sun, Z.-Y. Wang, H.-Y. Ren, D.-F. Xing, N.-Q. Ren, B.-F. Liu, *Chem. Eng. J.* **2023**, *455*, 140702.

[82] J. Dai, Z. Wang, K. Chen, D. Ding, S. Yang, T. Cai, *Chem. Eng. J.* **2023**, *453*, 139889.

[83] Y. Wen, V. K. Sharma, X. Ma, *ACS ES&T Water* **2022**, *2*, 635-643.

[84] J. Wang, B. Li, Y. Li, X. Fan, F. Zhang, G. Zhang, W. Peng, *Advanced Science* **2021**, *n/a*, 2101824.

[85] H.-C. Zhang, Z.-X. Kang, J.-J. Han, P. Wang, J.-T. Fan, G.-P. Sheng, *Angew. Chem. Int. Ed.* **2022**, *61*, e202200093.

[86] W. Ren, G. Nie, P. Zhou, H. Zhang, X. Duan, S. Wang, *Environ. Sci. Technol.* **2020**, *54*, 6438-6447.

[87] W. Wang, Y. Liu, Y. Yue, H. Wang, G. Cheng, C. Gao, C. Chen, Y. Ai, Z. Chen, X. Wang, *ACS Catal.* **2021**, *11*, 11256-11265.

[88] J. Miao, Y. Zhu, J. Lang, J. Zhang, S. Cheng, B. Zhou, L. Zhang, P. J. J. Alvarez, M. Long, *ACS Catal.* **2021**, *11*, 9569-9577.

[89] Y. Wei, J. Miao, J. Ge, J. Lang, C. Yu, L. Zhang, P. J. J. Alvarez, M. Long, *Environ. Sci. Technol.* **2022**, *56*, 8984-8992.

[90] Y. Si, Z.-Y. Guo, Y. Meng, H.-H. Li, L. Chen, A.-Y. Zhang, C.-H. Gu, W.-W. Li, H.-Q. Yu, *Environ. Sci. Technol.* **2021**, *56*, 564-574.

[91] M. Yang, Z. Hou, X. Zhang, B. Gao, Y. Li, Y. Shang, Q. Yue, X. Duan, X. Xu, *Environ. Sci. Technol.* **2022**, *56*, 11635-11645.

[92] E.-T. Yun, H.-Y. Yoo, H. Bae, H.-I. Kim, J. Lee, *Environ. Sci. Technol.* **2017**, *51*, 10090-10099.

[93] R. Su, N. Li, Z. Liu, X. Song, W. Liu, B. Gao, W. Zhou, Q. Yue, Q. Li, *Environ. Sci. Technol.* **2023**, *57*, 1882-1893.

[94] F. Chen, L.-L. Liu, J.-H. Wu, X.-H. Rui, J.-J. Chen, Y. Yu, *Adv. Mater.* **2022**, *34*, 2202891.

[95] Y. Zong, X. Guan, J. Xu, Y. Feng, Y. Mao, L. Xu, H. Chu, D. Wu, *Environ. Sci. Technol.* **2020**, *54*, 16231–16239.

[96] Y. Zong, Y. Shao, Y. Zeng, B. Shao, L. Xu, Z. Zhao, W. Liu, D. Wu, *Environ. Sci. Technol.* **2021**, *55*, 7634-7642.

[97] Y. Gong, Y. Wu, J. Shen, S. Zhao, X. Xu, J. Kang, L. Shen, Y. Zhou, Y. Zhao, Z. Chen, *Appl. Catal. B: Environ.* **2022**, *316*, 121671.

[98] X. Li, R. Lv, W. Zhang, M. Li, J. Lu, Y. Ren, Y. Yin, J. Liu, *Water Res.* **2023**, *228*, 119363.

[99] E. T. Yun, J. H. Lee, J. Kim, H. D. Park, J. Lee, *Environ. Sci. Technol.* **2018**, *52*, 7032-7042.

[100] J. Lee, J. D. Fortner, J. B. Hughes, J.-H. Kim, *Environ. Sci. Technol.* **2007**, *41*, 2529-2535.

[101] W. R. Haag, J. Hoigne, *Environ. Sci. Technol.* **1986**, *20*, 341-348.

[102] R. Natumi, C. Dieziger, E. M. L. Janssen, *Environ. Sci. Technol.* **2021**, *55*, 15196-15205.

[103] C. A. Davis, K. McNeill, E. M. L. Janssen, *Environ. Sci. Technol.* **2018**, *52*, 9908-9916.
